# Receptor tyrosine kinase C-kit promotes a destructive phenotype of FLS in osteoarthritis via intracellular EMT signaling

**DOI:** 10.1186/s10020-023-00633-6

**Published:** 2023-03-23

**Authors:** Xu Cao, Song Wu, Xinxing Wang, Junjie Huang, Wenxiu Zhang, Chi Liang

**Affiliations:** 1 Department of Orthopaedics of the 3rd Xiangya Hospital, Central South University, 138 Tongzipo Road, Changsha, 410013 China; 2 Institute of Basic Medicine and Cancer (IBMC), Chinese Academy of Sciences, Beijing, China

**Keywords:** C-kit (CD117), Epithelial-mesenchymal transition, Fibroblast-like synoviocytes, Osteoarthritis

## Abstract

**Background:**

Chronic inflammation, mainly derived from fibroblast-like synoviocytes (FLSs), plays a central role in the pathomechanism of osteoarthritis (OA). Recently, epithelial-mesenchymal transition (EMT) signaling was found to be activated in OA-derived FLSs with a pro-inflammatory phenotype. However, the role of EMT signaling in regulating FLS function and OA-related inflammation remains unknown.

**Methods:**

The synovium of OA patients were evaluated for EMT and inflammation markers. The FLSs with activated EMT signaling were co-cultured with chondrocytes (chond). Gene expression of OA synovial samples were analyzed. The role of receptor tyrosine kinase C-kit was investigated in OA-FLSs and an OA rat model. The downstream pathways driven by C-kit were explored in OA-FLSs.

**Results:**

EMT marker N-cadherin (N-CDH) was upregulated in 40.0% of the OA samples. These N-CDH^**+**^ OA samples showed higher expression of pro-inflammatory factors. In co-culture, FLSs derived from N-CDH^**+**^ OA samples induced a typical degenerative phenotype of chonds and stimulated their production of matrix degrading enzymes. C-kit was significantly upregulated and spatially co-localized with N-CDH in N-CDH^**+**^ OA samples. In OA-FLSs, C-kit activated intracellular EMT signaling and induced destructive features of OA-FLSs. In OA rat model, C-kit largely promoted synovial inflammation and cartilage destruction, whereas knocking-down C-kit significantly restored the health of OA joints. Using GSK3β S9A mutant, we demonstrated that C-kit drives EMT signaling in OA-FLS by promoting phosphorylation of GSK3β and nuclear retention of the EMT transcription factor Snail.

**Conclusion:**

C-kit drives EMT signaling in OA-FLSs and promotes a destructive FLS phenotype, leading to synovial inflammation and cartilage destruction.

**Supplementary Information:**

The online version contains supplementary material available at 10.1186/s10020-023-00633-6.

## Introduction

Osteoarthritis (OA) is a whole joint disease caused by multifactorial disorders. Chronic low-grade inflammation, which is the most frequent finding in OA patients, has been found to play a central role in the pathomechanism of OA (Robinson et al. 2016). Low-grade inflammation, mainly derived from fibroblast-like synoviocytes (FLSs), causes damage to intra-articular chondrocytes and the cartilage matrix by continually upregulating inflammatory mediators (For example IL-1β, 6 and TNFα) and matrix degrading enzymes (For example MMP1, 3 and 13), leading to progression of OA. Although several mechanisms have been found to potentially play a role in the induction of this OA-related inflammation, such as damage associated molecular patterns (DAMPs), senescence associated secretory phenotype (SASP) and lipid metabolism disorders (Sokolove and Lepus 2013; Coryell et al. 2021; Jeon et al. 2017; Collins et al. 2021; Cao et al. 2022). However, the predominant pro-inflammatory mechanisms in OA still remains largely unknown and controversial.

Epithelial-mesenchymal transition (EMT) is a process in which cells acquire mesenchymal phenotype. Intracellular EMT signaling, in addition to inducing the mesenchymal characteristics, also promotes the pro-inflammatory and invasive (activated cell migration and matrix degrading activity) phenotype of the cells (Lamouille et al. 2014). In rheumatoid arthritis (RA), EMT signaling was found to be a hallmark of FLSs with a tumor-like invasive phenotype (Lauzier et al. 2016; Steenvoorden et al. 2006). Recently, multiple bioinformatics studies have found that differentially expressed genes in OA synovium are enriched in several pathways, including EMT signaling (Ye et al. 2021; Todhunter et al. 2019; Cao et al. 2021). Similarly, EMT signaling was found to be activated in OA-derived FLSs with a pro-inflammatory phenotype (Cao et al. 2022). However, the role of EMT signaling in regulating FLS function and OA-related inflammation remains unknown. In theory, EMT signaling may promote the production of pro-inflammatory factors by FLS and the paracrine effects on chondrocytes. In addition, EMT signaling may enhance the invasive and matrix degrading activities of FLS and damage cartilage directly (Suarez-Carmona et al. 2017; Nygaard and Firestein 2020). In this study, we investigated the hypothesis that the activated EMT signaling of OA FLS could damage cartilage with the enhanced invasion activity and mass production of proinflammatory factors by an in vitro co-culture system and an OA rat model.

## Materials and methods

### Data collection and analysis

Raw data from 70 OA samples were extracted from 7 GEO databases (https://www.ncbi.nlm.nih.gov/geo/) (Supplementary Table S1) and preprocessed with RMA algorithm normalization using the “affy” R package. Potential batch effects were removed using “removeBatchEffect” from the “limma” R package. A total differentially expressed genes (DEGs) were identified between N-CDH^**+**^ and N-CDH^**−**^ OA synovial samples (with stricter screening criteria P ​< ​0.05, |LogFC| ​> ​0.5). Then the intersection of these DEGs and receptors involved in EMT signaling were analyzed with Venn diagrams and PPI network. The list of receptors involved in EMT was taken from a review of EMT molecular mechanisms (Lamouille et al. 2014) (Supplementary Table S2).

### Tissue collection and cell isolation

Human synovium and cartilage samples were collected during total knee arthroplasty (advanced OA, n = 10), and amputation (non-OA, n = 10) (Supplementary Table S3). The cartilage was obtained from the tibial plateau and distal femur of the knee, and the harvested FLSs and chondrocytes were isolated and cultured by a previously described method (Cao et al. 2022).

### Co-culture assay

A co-culture system was established using six-well Transwell plates (3428, Corning, NY, USA) in which 3 × 10^5^ chondrocytes in the same batch were cultured in the lower compartments and 3 × 10^5^ FLSs derived from N-CDH^**+**^ and N-CDH^**−**^ OA groups (n = 3) were cultured in the upper compartments in DMEM with 10% FBS. Chondrocytes were cultured alone as a control, and all co-cultures were maintained for 7 days before evaluation. The co-cultures were conducted in biological triplicate for each assay.

### Cell transfection and infection

The plasmids (pCDNA-GSK3β wild type and pCDNA-GSK3β S9A mutant) and lenti-virus (pCDNA-Ctrl, pCDNA-C-kit, plko-EGFP-shCtrl, and plko-EGFP-shC-kit) used in this experiment were purchased from Shanghai Tsingke Biotechnology Co., Ltd. All plasmids were constructed with a puromycin resistance. For cell transfection, FLSs from the same batch were transfected with various plasmids using Lipofectamine 3000 (L3000-015, Invitrogen, CA, USA) reagent according to the manufacturer’s protocol. For cell infection, FLSs from the same batch were infected with the lentivirus in the presence of polybrene (5 µg/ml) with centrifugation at 1,800 rpm for 40 min at 30 °C. Finally, the knockdown efficiency of C-kit was detected by immunoblotting with anti-C-kit antibody (18696-1-AP, Proteintech, Wuhan, China). The specific shRNA sequences are shown in Supplementary Table S4.

### Animal experiments

All Sprague Dawley (SD) rats used in animal experiments were provided by the Department of Laboratory Animals of Central South University. The OA rat model was established by performing left knee joint surgery using a Hulth method (Ma et al. 2017). 12-week-old female rats were divided into operated sham group + sh Ctrl injection (Sham), Hulth’s model + sh Ctrl injection (Control), Hulth’s model + sh C-kit injection. Briefly, after administering anaesthesia, rat’s left knee was skin perpetrated and sterilized, and then an anteromedial incision was made to expose the articular cavity. After joint-space opening, anterior cruciate ligament transection (ACLT) was performed to cut off two-thirds of the medial meniscus. Sham surgery was performed by making a skin incision at the same location in the left knee. After this procedure, we injected 20 µl 1 × 10^8^ TU/ml lenti-virus packaged empty vector (Sham group and Control group, n = 6 for each group) or EGFP-shC-kit (Hulth + shC-kit group, n = 6) intra-articularly once a week until 14 days after surgery. After 3 and 6 weeks, the rats were randomly selected for sacrifice, and cartilage and synovial samples were collected from each group for cell culture, RT-qPCR, western blot, and histological evaluation (see the corresponding sections of Material and Methods below).

### Histological evaluation

For the histological evaluation, the joints samples derived from rats were scanned by magnetic resonance imaging (7.0T MRI Biospoin GmbH, BRUKER, USA) and graded by their MOAKS score (Hunter et al. 2011). All the samples were then decalcified in 0.5 M EDTA ( G1105, Servicebio, Wuhan, China) for 4 weeks, and cut into sagittal Sect. (5 μm). Immunohistochemistry and immunofluorescence were performed using anti-N-cadherin (66219-1-lg, Proteintech, Wuhan, China), anti-MMP13 (#41,154, Signalway antibody, CA, USA), or anti-C-kit antibodies. Slices of rat knee joints were also stained with safranin O/fast green (G1371, Solarbio, Beijing, China). The Osteoarthritis Research Society International (OARSI) scoring system including cartilage, subchondral bone, osteophyte and synovitis was used to evaluate the OA cartilage pathology (Glasson et al. 2010).

### Western blot

Total proteins obtained from cells and tissues were subjected to SDS-PAGE, then transferred and blocked in 5% skimmed milk for 30 min. The membranes were incubated overnight at 4 °C with primary antibodies against IL-6 (#53,904, Signalway antibody, CA, USA), IL-8 (27095-1-AP, Proteintech, Wuhan, China), MMP-13, N-cadherin, E-cadherin (20874-1-AP, Proteintech, Wuhan, China), Vimentin (10366-1-AP, Proteintech, Wuhan, China), Col I (bs-0578R, Bioss, Beijing, China), Col II (GB11021, Servicebio, Wuhan, China), Col X (DF13214, Affinity, NJ, USA), C-kit, p-AKT (66444-1-AP, Proteintech, Wuhan, China), AKT (51077-1-AP, Proteintech, Wuhan, China), p-STAT3 (#11,045, Signalway antibody, CA, USA), STAT3 (#41,464, Signalway antibody, CA, USA), p-Erk1/2 (#12,082, Signalway antibody, CA, USA), Erk1/2 (#29,162, Signalway antibody, CA, USA), p-GSK3β (67558-1-Ig, Proteintech, Wuhan, China), GSK3β (22104-1-AP, Proteintech, Wuhan, China), p-Snail (63,568, Abcam, Cambs, UK), Snail (13099-1-AP, Proteintech, Wuhan, China) and GAPDH (10494-1-AP, Proteintech, Wuhan, China). Afterwards, they were incubated with HRP-conjugated secondary antibody (SA00001-1 or SA00001-2, Proteintech, China) at room temperature for 1 h and developed in electrochemiluminescence (ECL) Western blot detection reagents (BL520A, Biosharp, Beijing, China). The band was analyzed by UVP Chem studio PLUS 815 (Analytik jena, Germany). There are three biological replicates for Western blot.

### RT-qPCR

Total RNA was isolated from synovium or cells by TRIzol reagent (15,596,026, Thermofisher, MA, USA) and tested by NanoPhotometer® spectrophotometer (IMPLEN, CA, USA). Next, the RNA was converted to cDNA following the manufacturer’s instructions (R223-01, Vazyme, Nanjing, China). ChamQ Universal SYBR qPCR Master Mix (Q711-02, Vazyme, Nanjing, China) was used for qPCR testing according to the manufacturer’s protocol, and gene transcription levels (N-cadherin, E-cadherin, MMP1, MMP3, MMP13, IL-1β, IL-6, IL-8, IL-32, CDK1, TIMP1, TNFa, SAA1, S100A8, S100A9 and Vimentin) were normalized to those of GAPDH or β-actin. The primer design is shown in Supplementary Table S4.

### ELISA

The collected culture medium of OA-FLSs was analyzed by using the Human IL-6/IL-8 ELISA Kit (JM-03204H2, Jingmei Biological Technology, Shenzhen, China), according to the instructions given in the manual.

### Statistical analysis

All experiments were repeated at least three times and the data were presented mean with ± SD by individual dot plots unless otherwise noted. We performed all our statistical analysis with GraphPad Prism 8. Statistical significance was determined by t tests (two-tailed) for two groups. For the observational experiment in vivo, one-way ANOVA was used for comparisons across multiple groups, and Dunnett’s test was used for post-hoc multiple comparisons. The time dependent experiments were calculated with Two-Way ANOVA followed by Tukey’s multiple comparisons test. Bioinformatic analysis and visualization was carried out using R version 4.0.3 (https://www.r-project.org/). Specifically, DEGs between two subclusters were calculated with the R package limma”, and heatmaps were produced with the “pheatmap” R package. Finally, we analyzed the PPI network STRING version 11.5 (https://cn.string-db.org/) and visualized it with Cytoscape version 3.6.0.

## Results

### EMT signaling indicates an enhanced pro-inflammatory activity of OA synovium

**Fig. 1:**
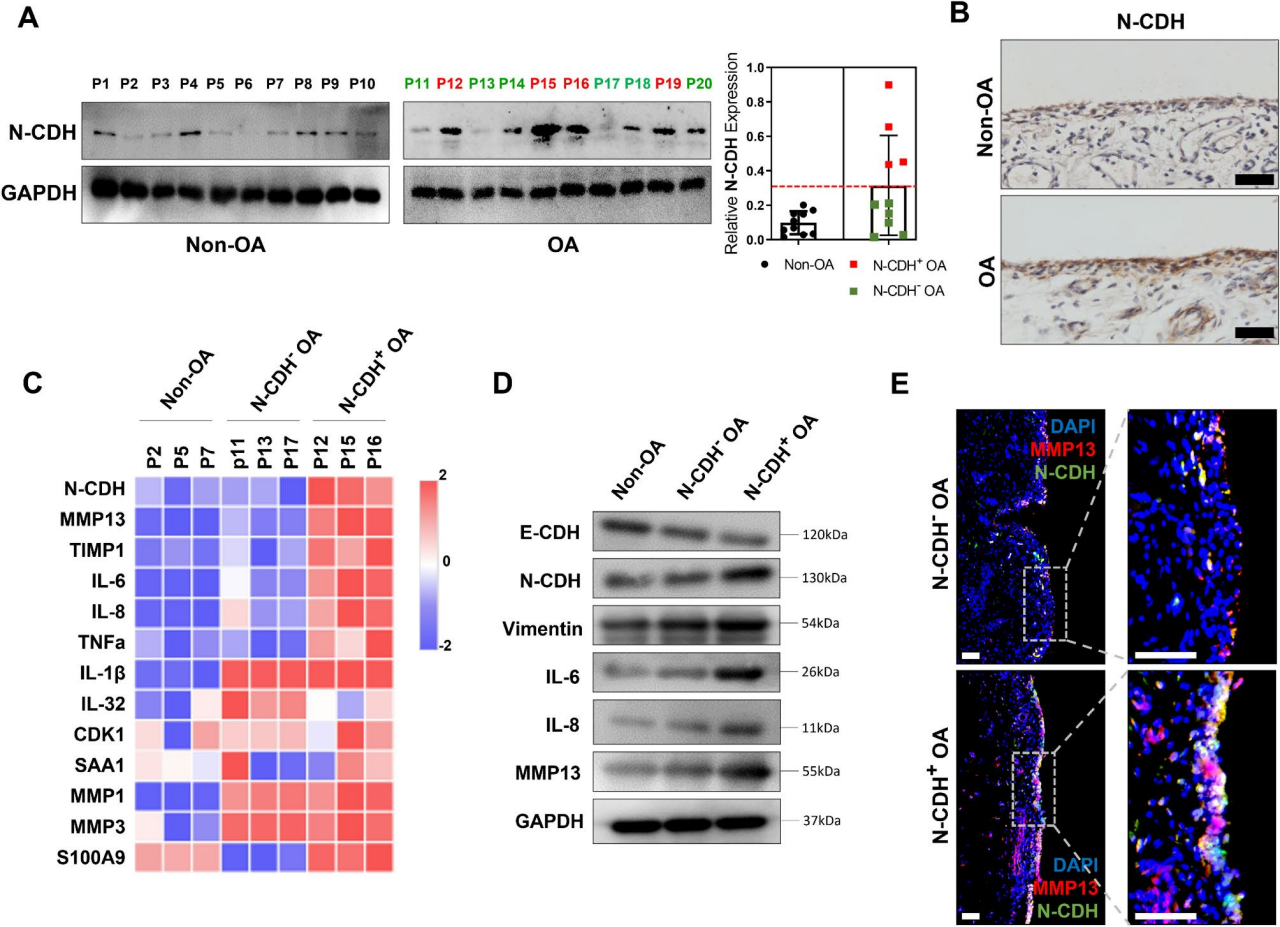
**EMT Signaling is closely related to chronic low-grade inflammation in OA synovium** (A-B) Western blot (A) and immunohistochemical staining (B) of N-cadherin in OA and Non-OA synovium. Red dashed lines (A, right) divide the patients into high (red) and low (black and green) subpopulations according to their N-CDH levels (n = 3, bar = 100 μm). (C) A heatmap for the mRNA expression of several common inflammatory factors in the non-OA, N-CDH^−^ and N-CDH^+^ OA subpopulations (n = 3). (D) Western blot of EMT markers and several positive inflammatory factors in (C). (E) The expression level and position of N-cadherin (green) and MMP13 (red) in the synovium of the N-CDH^−^ and N-CDH^+^ OA subpopulations (bar = 100 μm)

To explore the potential role of EMT signaling in regulating OA-related inflammation, we examined the EMT marker N-cadherin (N-CDH) in the synovial samples of 10 non-OA, and 10 OA patients. N-cadherin was upregulated (N-CDH^**+**^) in samples derived from OA patient 12, 15, 16 and 19 (Fig. 1A), and was primarily expressed in the hyperplastic synovial lining layer that composed mainly of pro-inflammatory FLSs (Fig. 1B). Next, we examined the expression of pro-inflammatory factors in these synovial samples. N-CDH^**+**^ OA samples (P12, 15 and 16) exhibited significantly higher levels of pro-inflammatory factors such as IL-6, IL-8, TNF-α and MMP13, compared to samples with low levels of N-CDH (including non-OA and N-CDH^**−**^ OA samples) (Fig. 1C and D). Histologically, fluorescence staining showed that the N-CDH and matrix degrading MMP13 were spatially co-localized in the FLSs of synovial lining layer (Fig. 1E). These all indicated a close association between EMT signaling and enhanced pro-inflammatory activity of OA-FLSs.

### EMT signaling promotes the degeneration of chondrocytes by paracrine effect

**Fig. 2:**
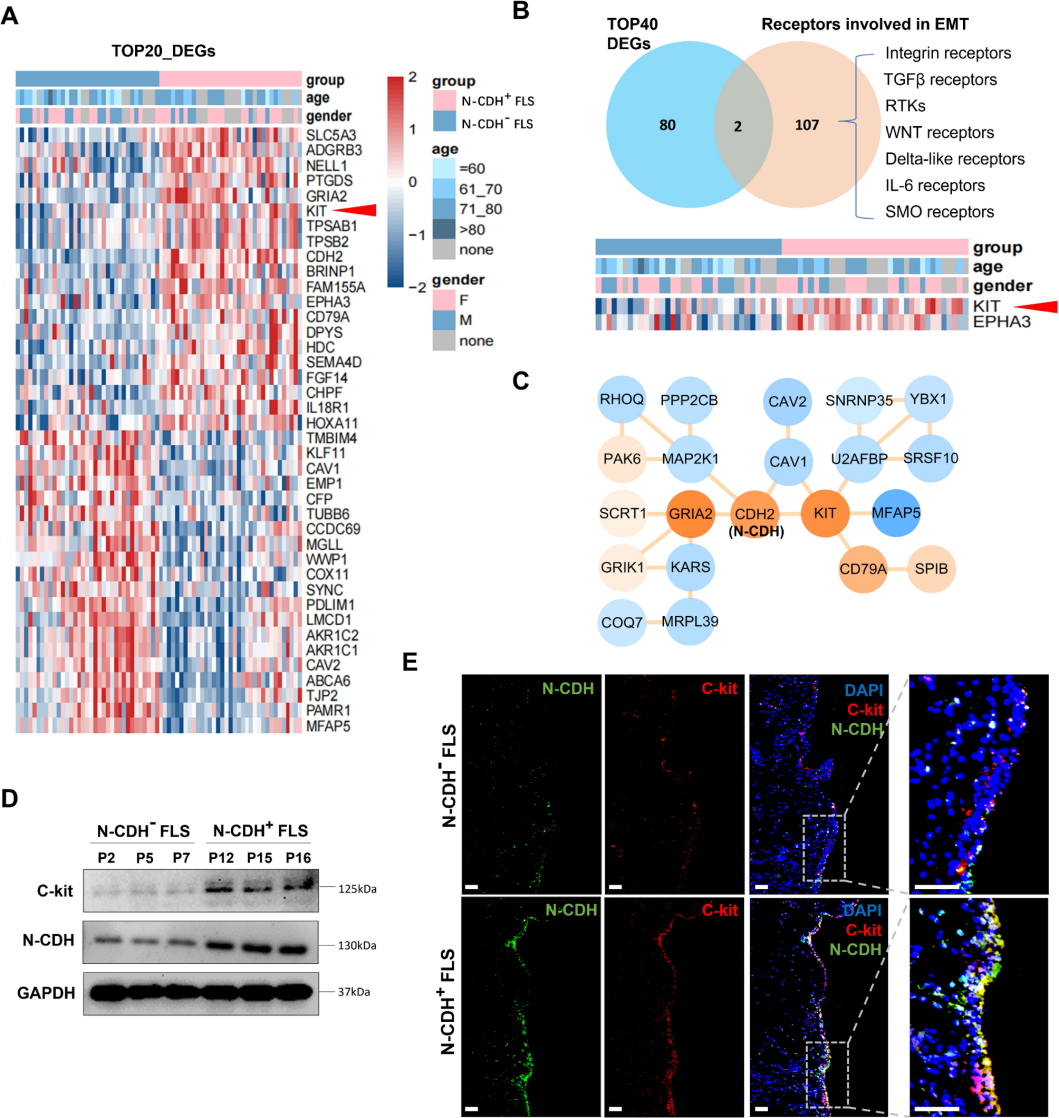
** N-CDH**+**FLS induces chondrocyte degeneration by paracrine** (A) Assessment of EMT markers in the FLSs derived from different patients with N-CDH^−^ and N-CDH^+^ OA by Western blot (n = 3). (B) Illustration of co-culture of OA-FLS and chondrocytes. (C-D) Expression of OA chondrocyte inflammation and degradation mechanism genes (MMP1, MMP3, MMP13, and COMP) after co-culture with N-CDH^−^ or N-CDH^+^ FLSs for 0, 1, 3, and 5 days or not. (n = 3, bar = 100 μm). (E-F) After co-culture for 7 days, assessment of cell hypertrophy (E, bar = 100 μm) and collagen synthesis ability (F) of the chondrocytes. ∗p < 0.05; ∗∗p < 0.01; ∗∗∗p < 0.001

Next, we explore the role of EMT signaling in regulating paracrine effect of FLS on chondrocytes (chonds). FLSs derived from the N-CDH+ or N-CDH- OA synovial samples were examined for EMT markers (Fig. 2A) and co-cultured with OA-chonds using a transwell system (Fig. 2B). FLSs with activated EMT signaling (N-CDH+ FLS) significantly stimulated the production of matrix degrading enzyme MMP1, 3 and 13 by OA-chonds, compared with N-CDH- FLSs and OA-chonds alone (Fig. 2C and D). Moreover, after co-culture for 7 days, N-CDH+ FLS promoted a typical degenerative phenotype of OA-chonds that characterized by reduced hyaline cartilage marker collagen type II (Col II), elevated fibrocartilage marker Col I and hypertrophic markers Col X and Runx2, whereas N-CDH- FLSs only reduced the expression of Col II of OA-chonds (Fig. 2E-F).

### Receptor tyrosine kinase C-kit drives EMT signaling in OA-FLS and promotes a destructive FLS phenotype

**Fig. 3:**
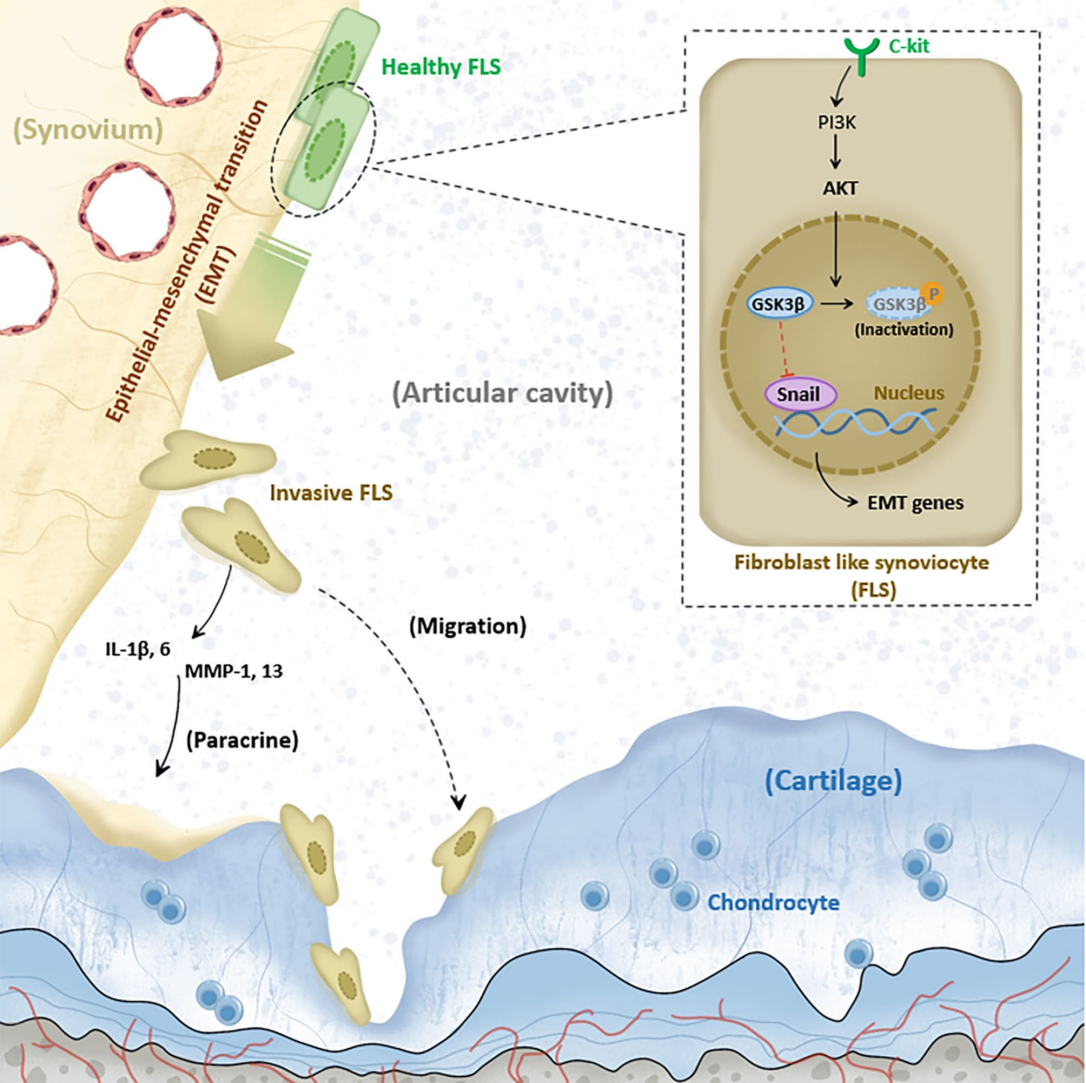
** C-kit increases in N-CDH**+**OA synovium** (A) The top 20 upregulated (or downregulated) differential genes in the cluster based on their expression level of N- cadherin in 70 OA synovial samples. (B) Venn diagrams and heatmaps based on the intersection between the top 40 differential genes and EMT-related receptors involved in EMT. (C) The PPI network of N-cadherin- and C-kit- (A-B, red arrowhead) related DEGs between the N-CDH^−^ or N-CDH^+^ subclusters. Orange and blue circles represent genes with high and low expression in the N-CDH^+^ subcluster, respectively. (D) C-kit expression in N-CDH^−^ or N-CDH^+^ OA synovium derived from different patients (n = 3). (E) The expression and location of C-kit (red) and N-cadherin (green) in two subpopulations of OA synovium by immunofluorescence (bar = 100 μm). N-CDH: N-cadherin; RTK: receptor tyrosine kinase; SMO: smoothed

To identify the mechanisms driving EMT signaling in OA-FLS, we analyzed the differentially expressed genes (DEGs) between N-CDH+ and N-CDH- OA synovial samples. A total of 243 DEGs were identified (with stricter screening criteria P ​< ​0.05, |LogFC| ​> ​0.5), and the top 20 upregulated or downregulated DEGs are shown in Fig. 3A. We examined the receptors that drive intracellular EMT signaling (including 107 genes) in these top 40 DEGs (Fig. 3B), and found that the receptor tyrosine kinase C-kit (KIT) was significantly upregulated in N-CDH+ OA synovial samples (Fig. 3A, B and D). Figure 3 C illustrates the PPI network centered on C-kit and N-CDH. Fluorescence staining showed that the C-kit and N-CDH were spatially co-localized in the OA-FLSs of synovial lining layer (Fig. 3E).

**Fig. 4:**
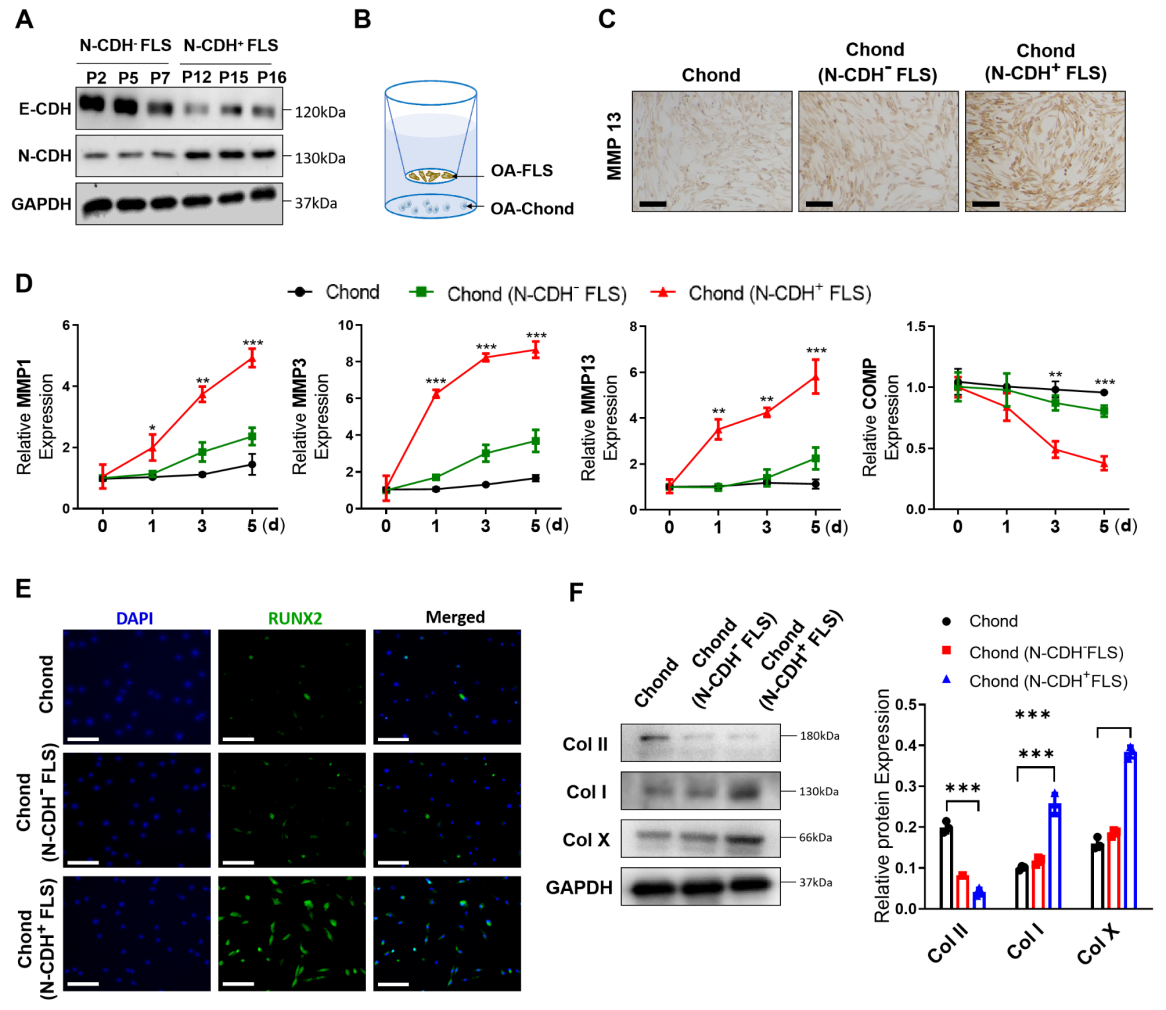
** C-kit drives FLS EMT signaling** (A) Validation of several shC-kit efficiency by Western blot (n = 3). (B-C) The expression of EMT makers (B) and Transwell invasive assay (C) in OA FLS with pCDNA-Ctrl, pCDNA-C-kit, shCtrl, or shC-kit transfection (n = 3, bar = 100 μm). (D) Morphological observation of DAPI (blue) and vimentin staining (green). Pseudopodia are marked by white arrowheads (bar = 20 μm). (E) The expression of inflammation-related markers (IL-6, IL-8, and MMP13) in FLSs (n = 3). (F) Illustration of co-culture of lenti-virus infected OA-FLS and chondrocytes. (G-H) After co-culture for 7 days, assessment of MMP13 (G) and Runx2 (H) of the chondrocytes (n = 5, bar = 100 μm)

To confirm the role of C-kit in the OA-FLS above, we overexpressed C-kit by pCDNA-C-kit or knock-down C-kit by shC-kit in OA-FLS (Fig. 4A and B). Overall, we found that C-kit promoted the EMT signaling and destructive features of OA-FLS, whereas knockdown of C-kit attenuated the EMT signaling and restored the features of a resting FLS. The EMT markers N-cadherin/E-cadherin and Vimentin were upregulated in C-kit overexpressed FLSs (Fig. 4B). C-kit promoted the expression of pro-inflammatory IL-8, IL-6 and matrix degrading MMP13 by FLSs (Fig. 4E). Moreover, with C-kit overexpression, FLSs exhibited increased invasive activity (Fig. 4C) and pseudopodia formation, whereas C-kit-silenced FLSs regained their resting morphology (Fig. 4D). In co-culture system (Fig. 4F), C-kit overexpressed FLSs stimulated the expression of matrix degrading factor MMP13 and degenerative marker Runx2 in OA-chonds, whereas C-kit silencing ameliorated this effect (Fig. 4G H).

### C-kit promotes synovial inflammation and cartilage destruction in an OA rat model

**Fig. 5:**
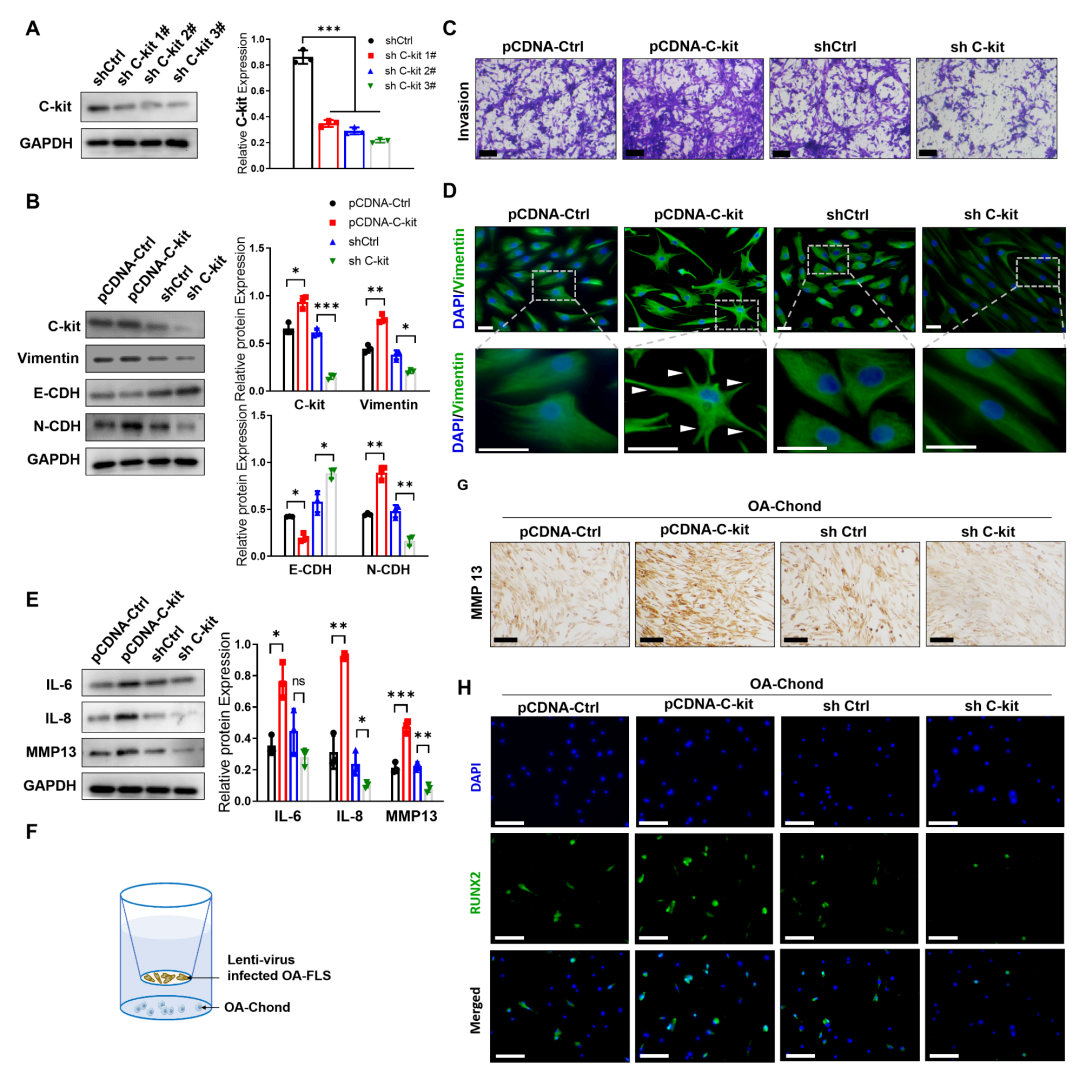
** C-kit inhibition protects rats from mechanical instability induced OA model** (A) Tracing of lenti-virus packaged EGFP-shC-kit (green) with intra-articular injection. (B) Expression of C-kit in synovium and cartilage derived from control, Hulth, and Hulth + shC-kit rats. (C) HE staining (above, bar = 500 μm) and amplified C-kit staining (below, bar = 100 μm) focused on synovium and cartilage (F: femur; T: tibia; M: meniscus; S: synovium) (n = 3). (D-E) EMT-related genes’ (D, E) and inflammatory factors’ (D) expression in rats synovium (n = 3). (F) Safranin O/fast green staining of cartilage (up) and OARSI semi-quantitative scoring system. including cartilage, subchondral bone, osteophyte and synovitis (bottom) (n = 3, bar = 800 μm). (G) The medial joint space (white arrow, between two dotted lines) in coronal position of total knee and MOAKS score by magnetic resonance imaging (n = 3, bar = 1000 μm). ∗p < 0.05; ∗∗p < 0.01; ∗∗∗p < 0.001. MFC: medial femoral condyle; MTP: medial tibial plateau

We utilized Hulth’s method to establish an OA model in SD rats. Lenti-virus of shC-kit was injected intra-articularly to knock-down C-kit (Fig. 5A-C). Six weeks after surgery, we observed that C-kit was upregulated in the lining layer of synovium (Fig. 5B C). With the upregulation of C-kit, EMT markers (N-cadherin/E-cadherin and Vimentin) and pro-inflammatory factors (IL-6 and MMP13) was increased in the synovium of OA rats (Fig. 5D and E), histological features of cartilage destruction (cartilage thinning and proteoglycan loss) were observed (Fig. 5F) and radiographic features of OA (joint space narrowing, bone marrow lesions, and synovitis) appeared in magnetic resonance imaging (MRI) (Fig. 5G). Notably, intra-articular shC-kit injection largely inhibited the expression of EMT markers and pro-inflammatory factors in synovium (Fig. 5D and E), restored the health and integrity of the OA cartilage (Fig. 5F) and improved the OA features in MRI (Fig. 5G) after 6 weeks, which was not obvious at 3 weeks after the operation (Fig. S1A).

### C-kit drives the EMT signaling in OA-FLS through the PI3K-GSK3β-Snail pathway

**Fig. 6:**
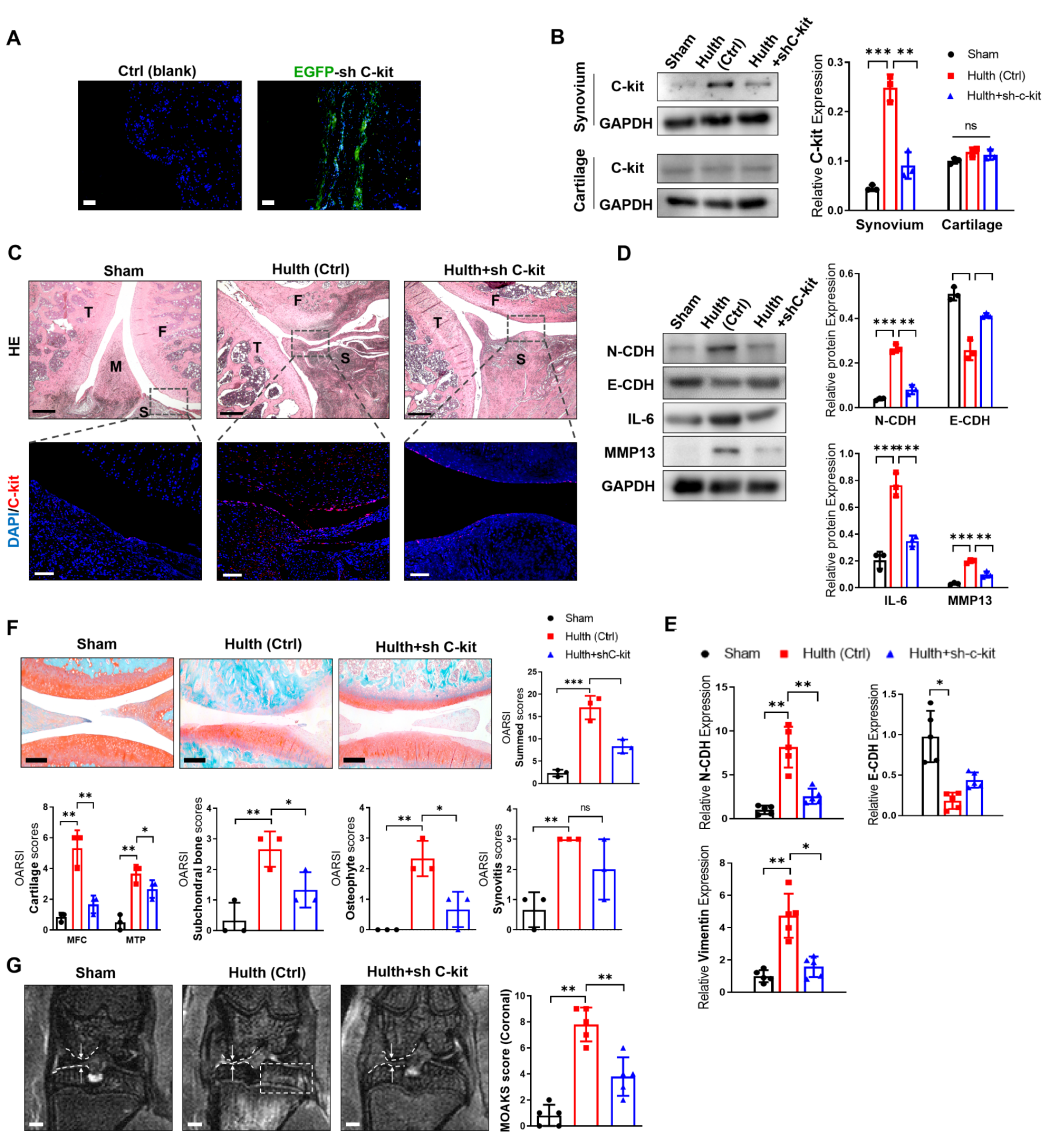
** C-kit promotes EMT signaling in OA-FLS by activating the AKT-GSK3β-Snail pathway** (A) The expression of EMT-related genes in FLSs derived from rat synovium (n = 5). (B) The effect of C-kit on the expression and phosphorylation of Akt, STAT3, and ERK1/2 in FLSs as determined by western blot (n = 3). (C) Nuclear translocation (white arrow) of Snail (green) and p-Snail (red) immunofluorescence (n = 3, bar = 50 μm). (D) Partial sequence of GSK3β WT and GSK3β S9A mutant. (E-F) Expression of phosphorylation of GSK3β (E), Snail (F), and EMT-related genes (E) in GSK3β WT- and GSK3β S9A-overexpressed FLSs treated with C-kit overexpression (or untreated) (n = 3, bar = 100 μm). ∗p < 0.05; ∗∗p < 0.01; ∗∗∗p < 0.001

We examined the four intracellular downstream pathways of receptor tyrosine kinase in OA-FLS, the MAPK, JAK/STAT, PI3K/AKT, and Src kinase pathways, and found that C-kit mainly activated the PI3K/AKT pathway and promoted AKT phosphorylation (Fig. 6A and B). Further, C-kit promoted phosphorylation of GSK3β, which is a substrate of AKT and a major negative regulator of the key EMT transcription factor Snail (Fig. 6B). Along with the phosphorylation of GSK3β (inactivation), we observed the dephosphorylation, stabilization, and nuclear retention of Snail in OA-FLSs (Fig. 6C). To examine the role of GSK3β, we transferred pCDNA-GSK3β WT and pCDNA-GSK3β S9A mutant (phosphorylation site by AKT) plasmids to OA-FLSs (Fig. 6D). Compared to GSK3β WT, S9A mutant stabilized GSK3β in C-kit induction by blocking AKT-induced phosphorylation (Fig. 6E). This GSK3β stabilization blocked C-kit induced EMT signaling in OA-FLS, including dephosphorylation of Snail (Fig. 6F) and expression of EMT markers N-cadherin and Vimentin (Fig. 6E).

**Fig. 7:**
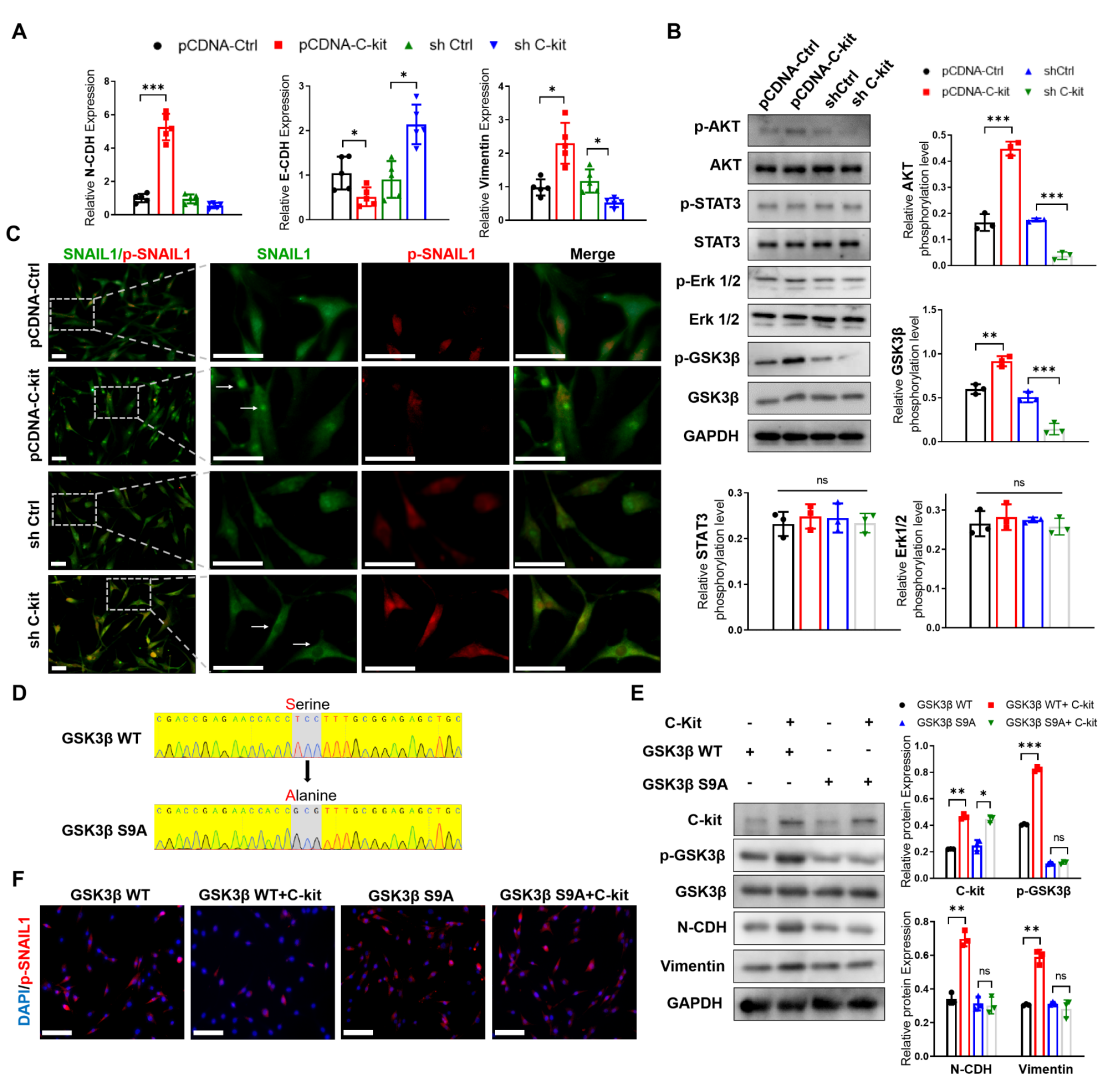
**Mechanism diagram** C-kit drives EMT signaling in OA-FLSs and promotes a destructive FLS phenotype, leading to synovial inflammation and cartilage destruction

The mechanism diagram of C-kit driving EMT signaling in OA-FLSs was showed in Fig. 7.

## Discussion

In this study, we demonstrated that EMT signaling serves as a key pro-inflammatory pathway in OA joint through inducing a destructive FLS phenotype. EMT marker N-CDH was upregulated in the OA samples and was primarily expressed in the hyperplastic synovial lining layer that composed mainly of FLS (Fig. 1B). In the past, more studies consider that EMT mainly plays a role in rheumatoid arthritis (RA), rather than in OA. These studies have found that EMT signaling mediates the activation of FLS in RA and plays a critical role in the formation of synovitis and pannus (Lauzier et al. 2016; Steenvoorden et al. 2006; Zhu et al. 2019; Chen et al. 2021; Li et al. 2013). In contrast, the EMT markers were found to be significantly lower in OA-FLSs than in RA-FLSs (Zhu et al. 2019). However, recently, multiple bioinformatics studies detected differentially expressed genes related to EMT signaling in OA synovial samples (Ye et al. 2021; Todhunter et al. 2019; Cao et al. 2021). For example, Ye T et al. identified top 4 hallmark “TNFA_SIGNALING VIA NFKB”, “EPITHELIAL MESENCHYMAL TRANSITION (EMT)”, “INFLAMMATORY RESPONSE” and “HYPOXIA” from enrichment of differentially expressed genes in OA synovium (Ye et al. 2021). This discrepancy is most likely attributable to the heterogeneity of OA pathomechanism. Yuan et al. (2020). Similarly, using unsupervised clustering of expression array profiles, OA synovial samples were categorized into 3 distinct subclusters with discrepant functional annotations, of which only the Cluster 3 (34.3%) exhibited tendency of synovial EMT (Cao et al. 2022). In this study, we also detected upregulation of EMT markers in only about 40% of OA synovial samples (Fig. 1A). We subsequently focused on this subset of samples to explore the role of EMT signaling for OA-related synovial inflammation and cartilage destruction.

The role of EMT signaling in regulating OA-related pathomechanism also remains largely unknown. EMT is known to be a process closely associated with chronic inflammation (Dominguez et al. 2017). We found that OA synovial samples with activated EMT signaling exhibited higher levels of pro-inflammatory factors compared to that with low EMT markers (Fig. 1C and D). Further, we found that this EMT process may occur in the FLS located in synovial lining layer. OA-FLS with higher levels of EMT markers generated more matrix degrading MMP13 (Fig. 1E). The pro-inflammatory property of EMT signaling has received extensive concern in tumor-driven inflammation (Dominguez et al. 2017). In one study, analysis of the “secretory phenotype” of tumor cells with activated EMT signaling revealed a distinct set of pro-inflammatory soluble factors, including IL-6, IL-8, GRO, GM-CSF, VEGF, and angiogenin (Fernando et al. 2011). Similarly, Suarez-Carmona et al. (2015). EMT transcription factors, such as Snail, upregulate the expression of extracellular-matrix-degrading MMPs to favor the migration of cells (Scheau et al. 2019). These secreted MMPs cause local tissue damage, such as destruction of articular cartilage, and the released matrix degradation products, namely DAMPs, activate inflammatory responses in the microenvironment, which is an important pathomechanism of OA (Sokolove and Lepus 2013). In addition to directly promoting inflammation and tissue damage, we found that EMT can also induce cartilage degeneration through paracrine effects. In transwell co-culture, FLS with activated EMT signaling stimulated the production of MMP1, 3 and 13 by OA-chondrocytes, and induced a typical degenerative chondrocyte phenotype characterized by reduced Col II, elevated Col I, Col X and Runx2 expression (Fig. 2C F). The exact mechanism underlying this paracrine effect is currently unknown. We speculate that it may be attributable to cytokines released by FLS such as IL-1β and IL-6.

Intracellular EMT signaling is highly regulated by a variety of mechanisms, including transmembrane receptors that act on Wnt signaling, TGF-β signaling, receptor tyrosine kinases (RTKs) signaling, and Notch signaling (Lamouille et al. 2014; Zhang et al. 2021). In this study, we found that C-kit, a transmembrane type III RTK, was significantly upregulated in OA-FLSs with higher EMT marker expression (Fig. 3). Subsequent experiments demonstrated that C-kit signaling drives the EMT process in OA-FLS through the PI3K-GSK3β-Snail axis (Figs. 4, 5 and 6). Studies have observed that 72–98% of FLSs derived from OA synovium are positive for C-kit (Hermida-Gómez et al. 2011; Gimeno et al. 2005). But in the context of different diseases, the main mechanism that drives EMT signaling are differential. In RA-affected joints, transforming growth factor β1 (TGF-β1) signaling is considered the most prominent pathway contributing to the EMT process of FLS (Lauzier et al. 2016; Zhu et al. 2019). Hypoxia induced Notch-1 signaling has also been shown to play a key role in driving EMT in RA-FLS (Chen et al. 2021). During active phase, infiltration of leukocytes and proliferation of synoviocytes resulted in increased oxygen consumption and local hypoxia. Therefore, most studies consider a hypoxic environment and excessive inflammatory cytokines as the main triggers of EMT signaling in RA-FLSs. Different from the highly activated inflammation in RA, an abnormal biomechanical environment is one of the most prominent pathophysiological features of OA (Egloff et al. 2012). Biomechanical environments, such as substrate stiffness, have been found to be critical factors in driving the progression of EMT. Biomechanical signals regulate multiple intracellular mechanical sensors that activate EMT-driving pathways (Broders-Bondon et al. 2018). In this study, The trigger that drives the upregulation of C-kit in OA-FLS is currently undetermined. Therefore, our further research may focus on the upstream mechanism of C-kit. According to the existing research data (unpublished), we have observed that abnormal tensile stress induced the upregulation of C-kit in OA-FLS in vitro. But more experiments are still needed to confirm this.

## Conclusion

In this study, we found that C-kit, a receptor tyrosine kinase, drives EMT signaling in OA-FLSs and promotes a destructive FLS phenotype, leading to synovial inflammation and cartilage destruction, which demonstrates its translational potential as a new target in clinical treatments for patients with OA.

## Electronic supplementary material

Below is the link to the electronic supplementary material.

Supplementary Material 1

Supplementary Material 2

Supplementary Material 3

Visualization for supplementary table 3?K/L grade: Kellgren & Lawrence grade (0-4 grade).

Supplementary Material 5

**Supplementary Figure legendsSupplementary Figure S1**. (A) Safranin O/fast green staining of cartilage (up), MRI images (bottom) and OARSI semi-quantitative scores of 3 weeks after operation (right). **Supplementary Table legendsSupplementary Table S1**. Basic information (gender, age, source, public date and clinical status) and data sources of 70 OA patients **Supplementary Table S2**. 107 EMT-related receptors. **Supplementary Table S3**. Basic information (gender, age, K/L grade) of 10 non-OA and 10 OA patient. K/L grade: Kellgren & Lawrence grade. **Supplementary Table S4**. The human and rat primer sequence for qPCR and knocking down.

**Supplementary Table S4**. The human and rat primer sequence.

## Data Availability

All data generated or analysed during this study are included in this published article [and its supplementary information files].
