# Supplementary figures and images for: Receptor tyrosine kinase C-kit promotes a destructive phenotype of FLS in osteoarthritis via intracellular EMT signaling
Source: Mol Med. 2023 Mar 23;29:38. doi: 10.1186/s10020-023-00633-6 (PMC10037859; doi:10.1186/s10020-023-00633-6)

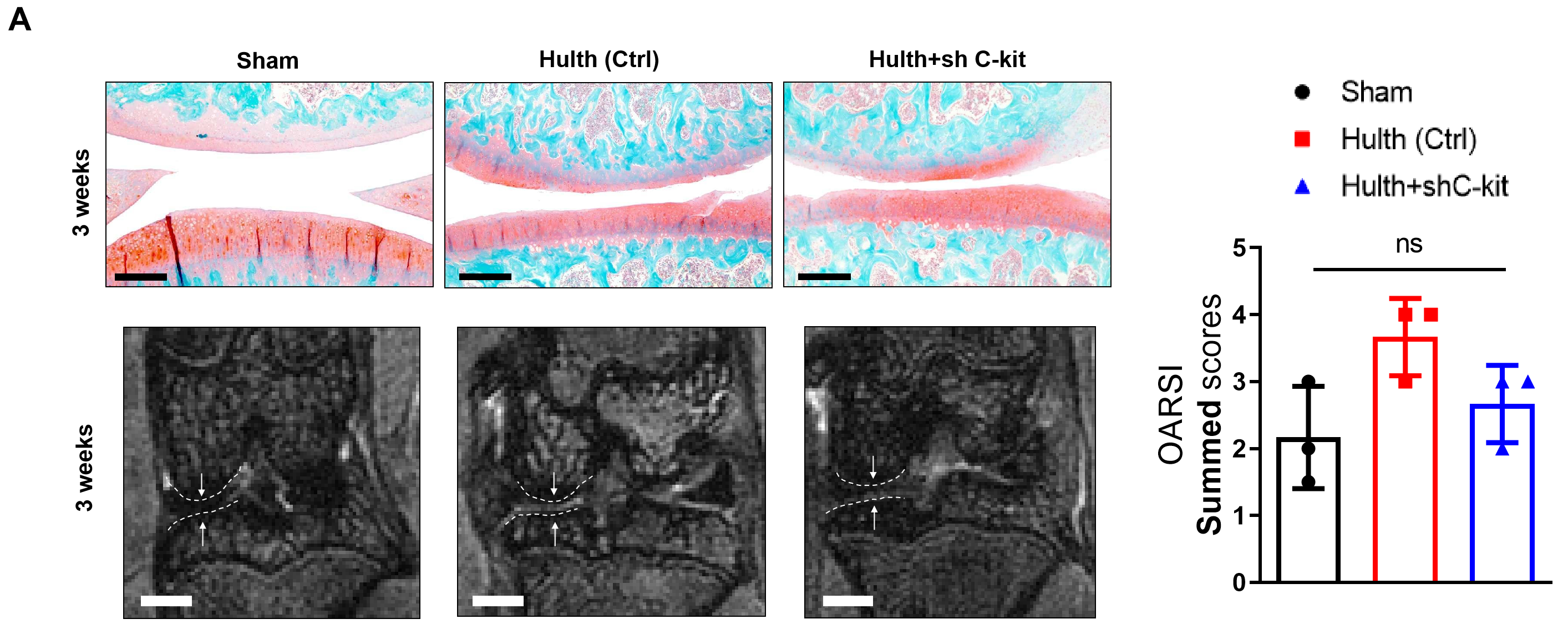

Supplement: Supplementary file 1 — Supplementary Material 1 [file 10020_2023_633_MOESM1_ESM.png]

Fig 1A:


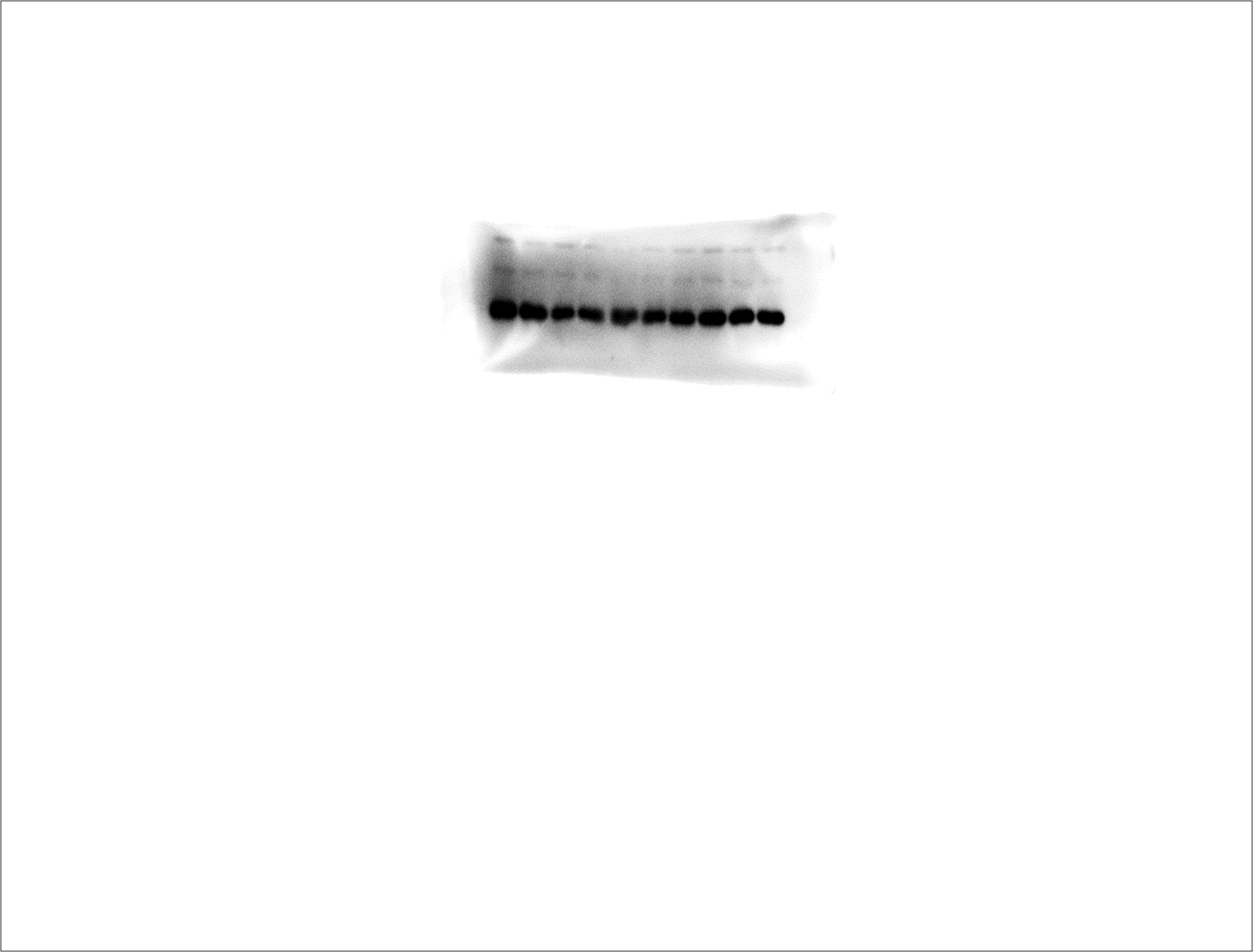

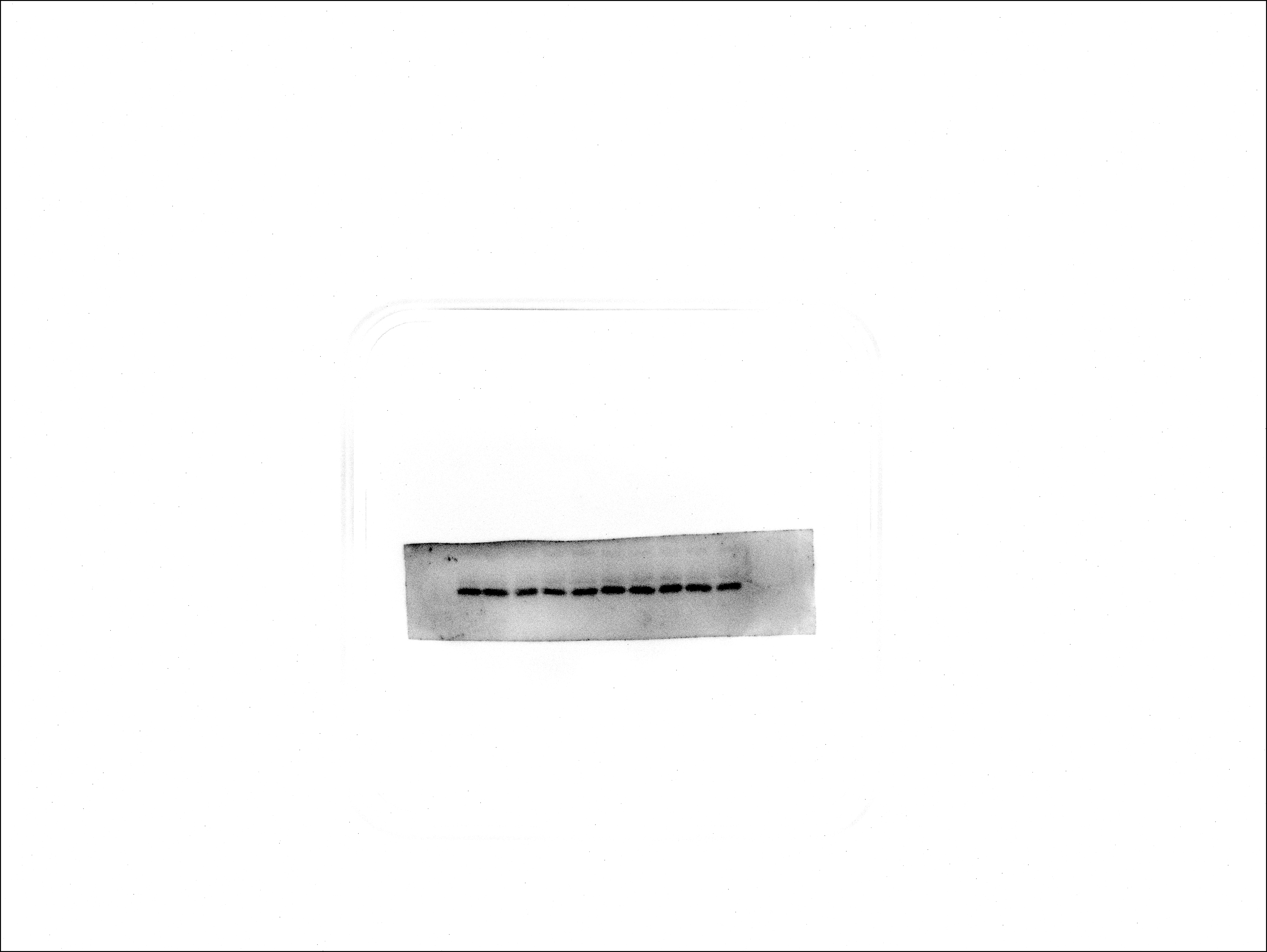

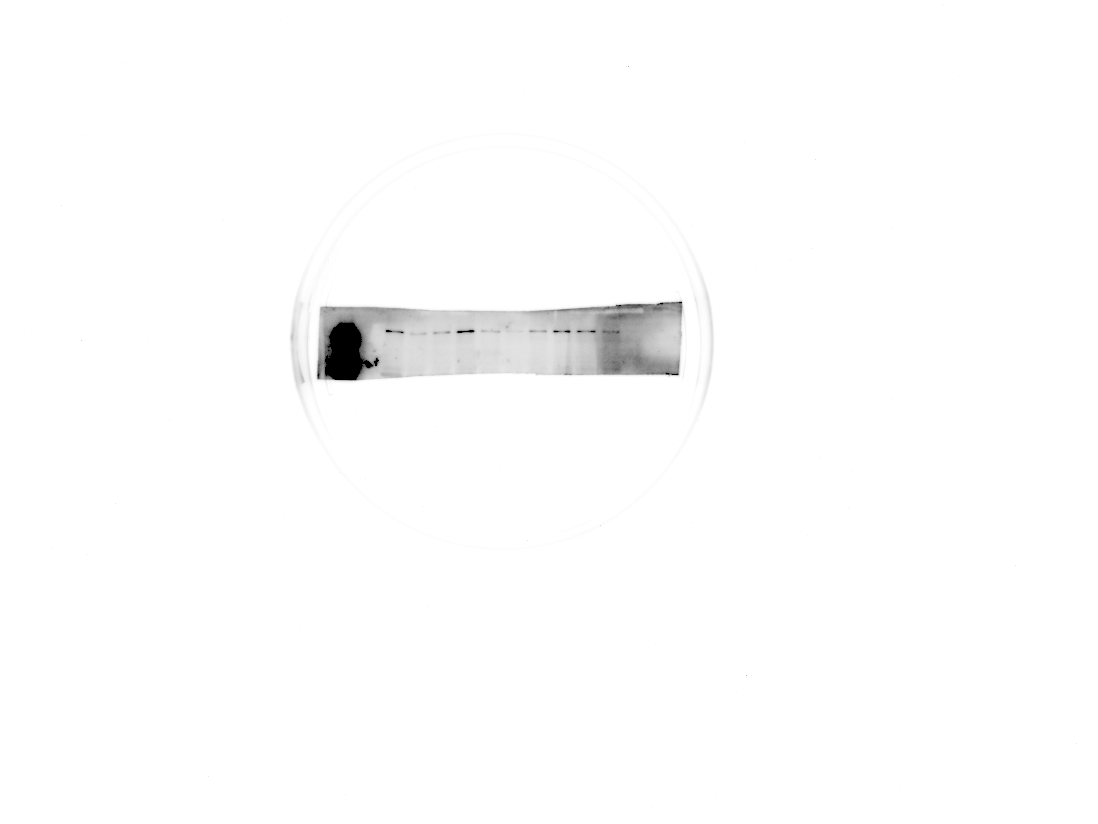

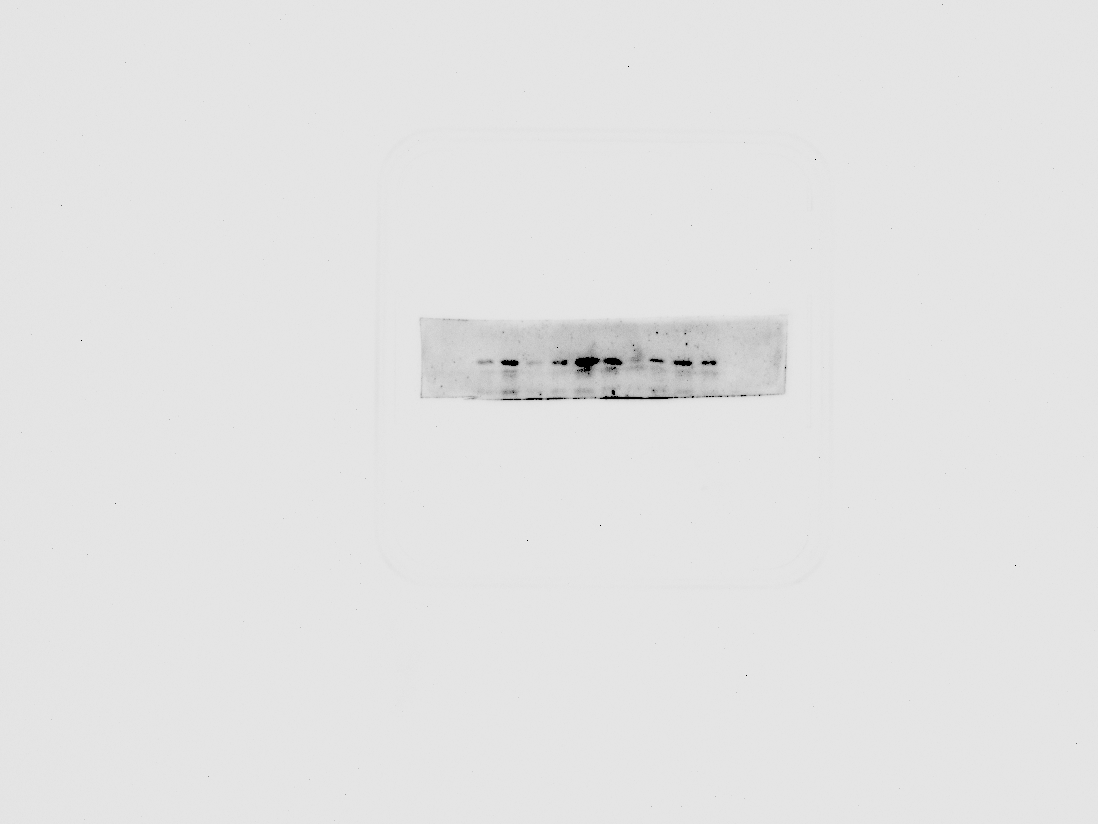


Fig1D:
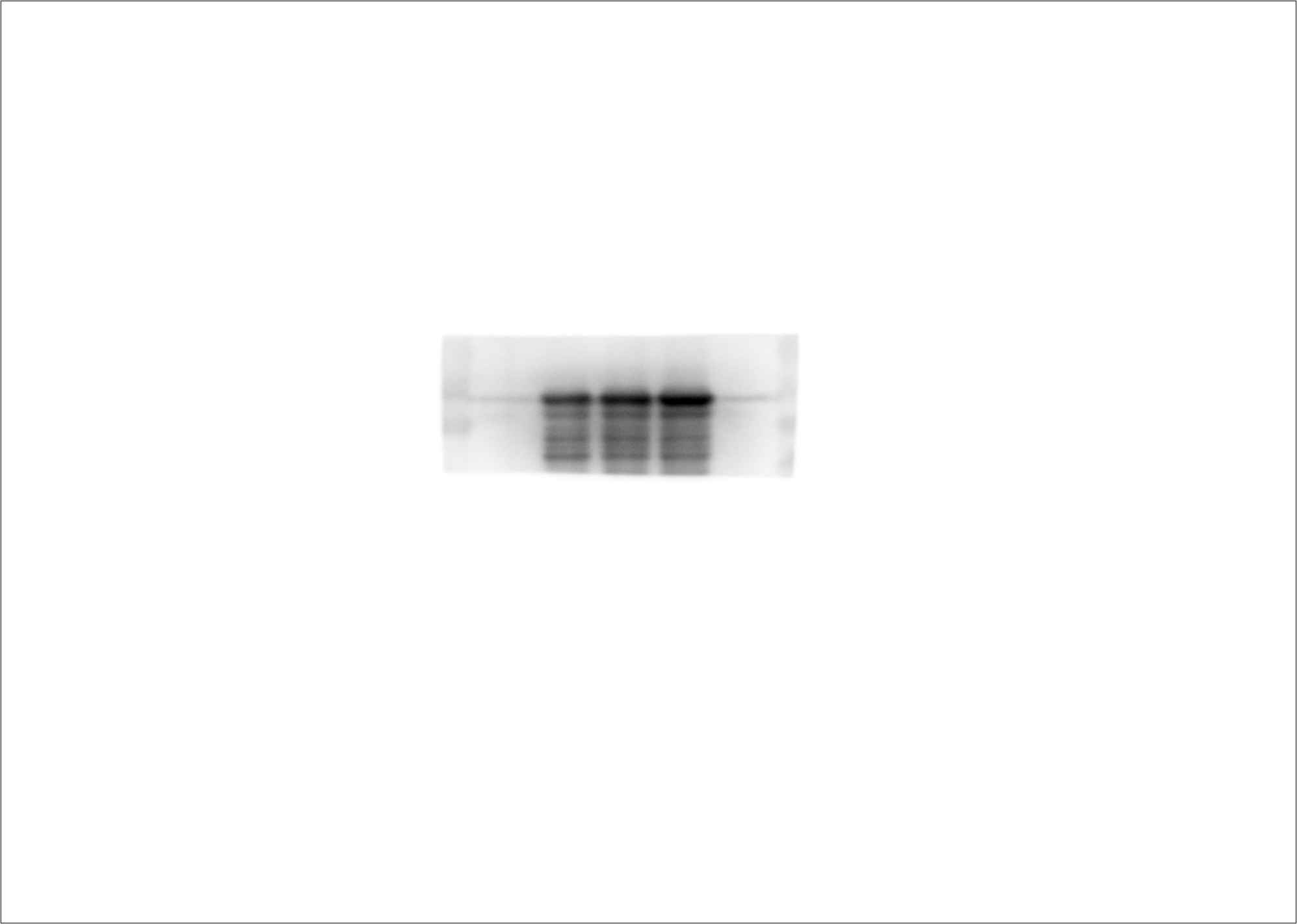

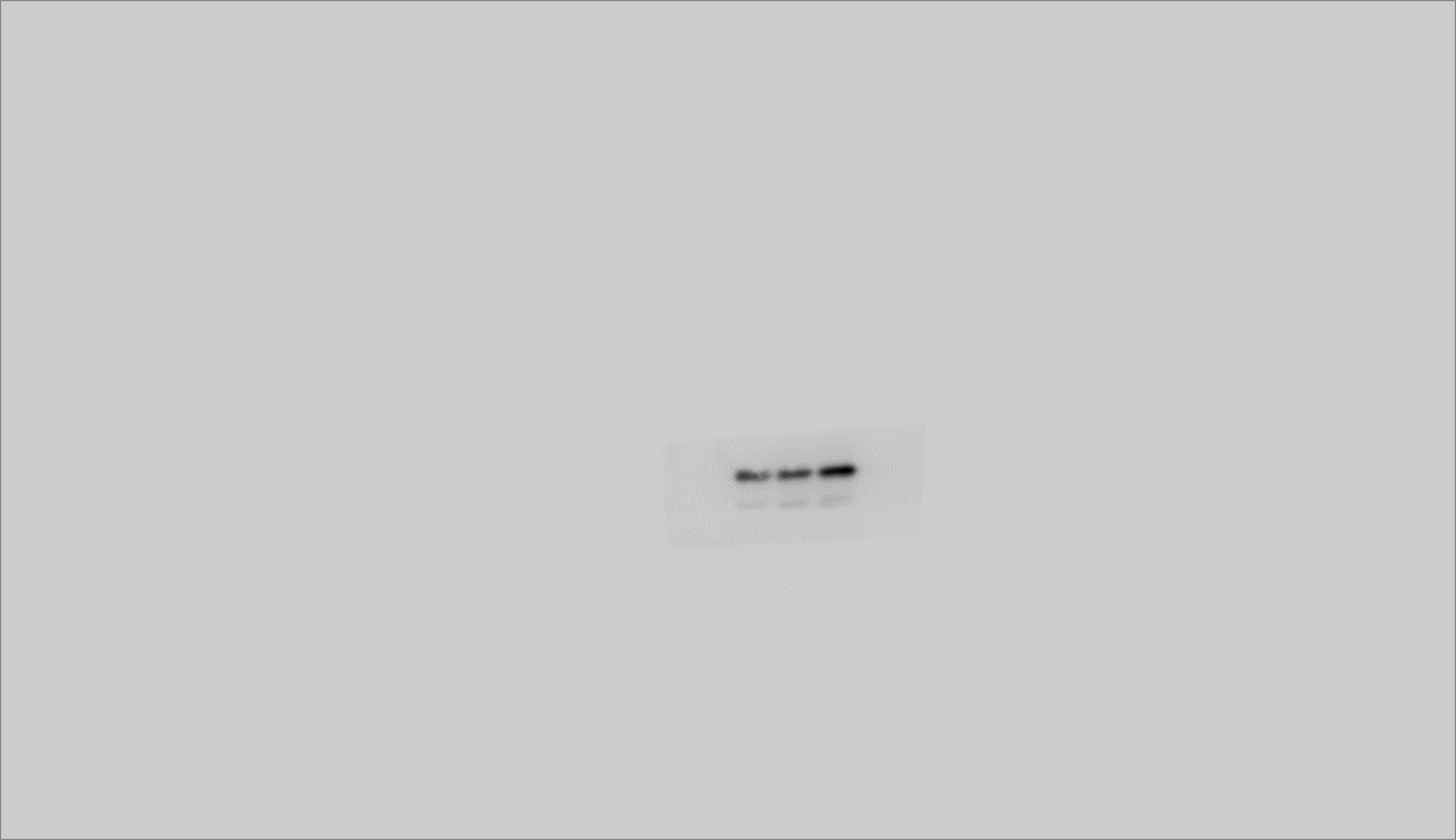

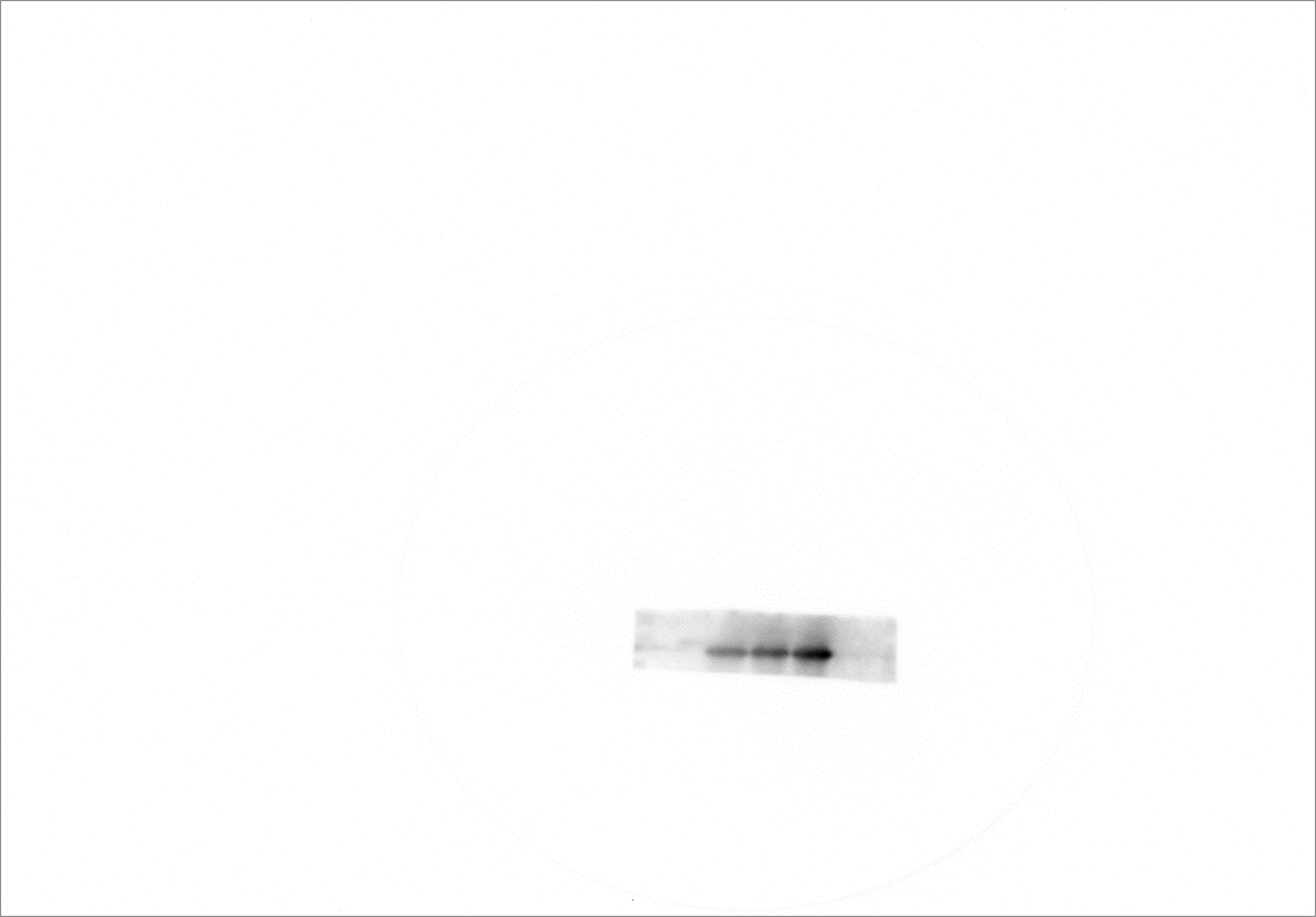

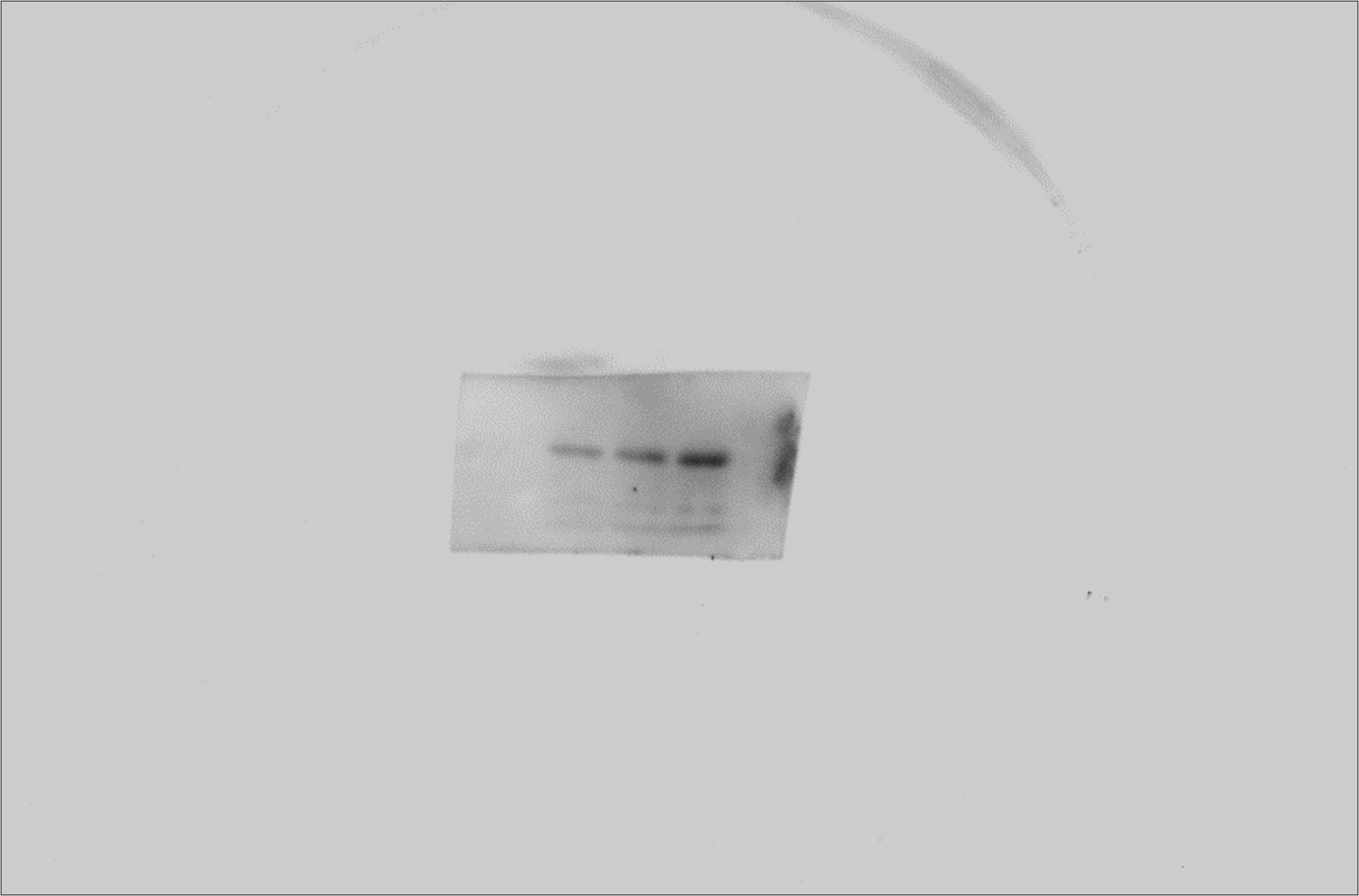

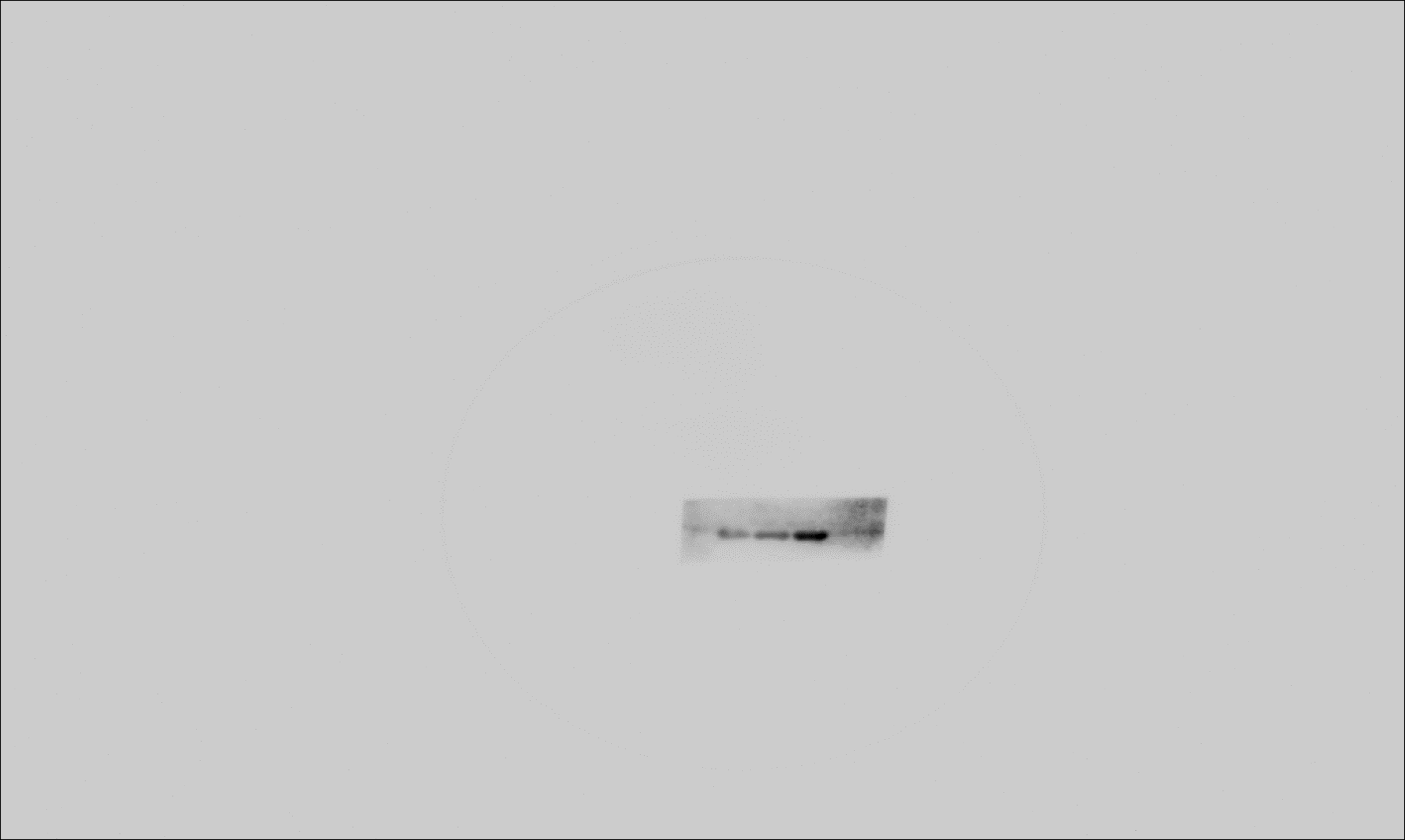

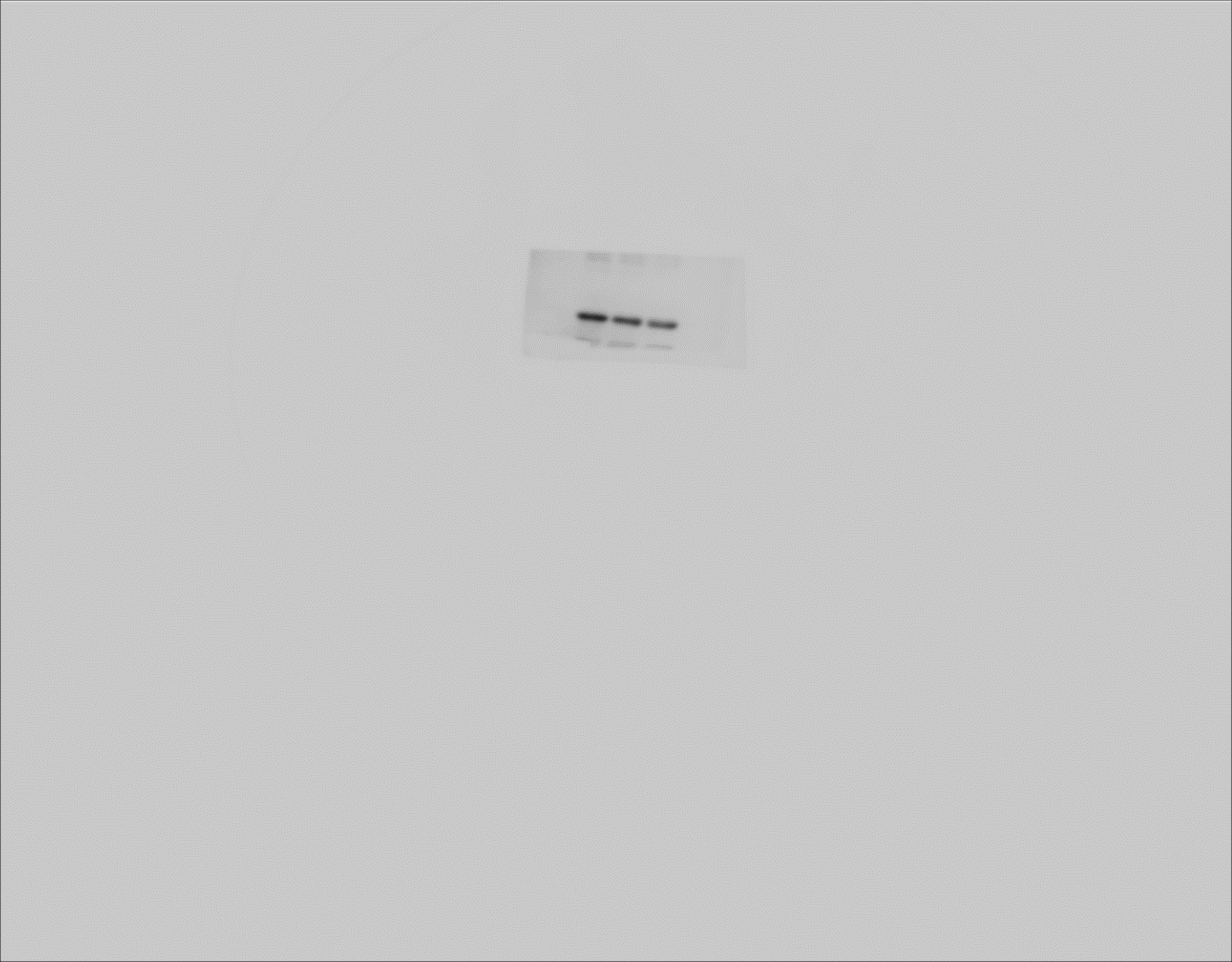

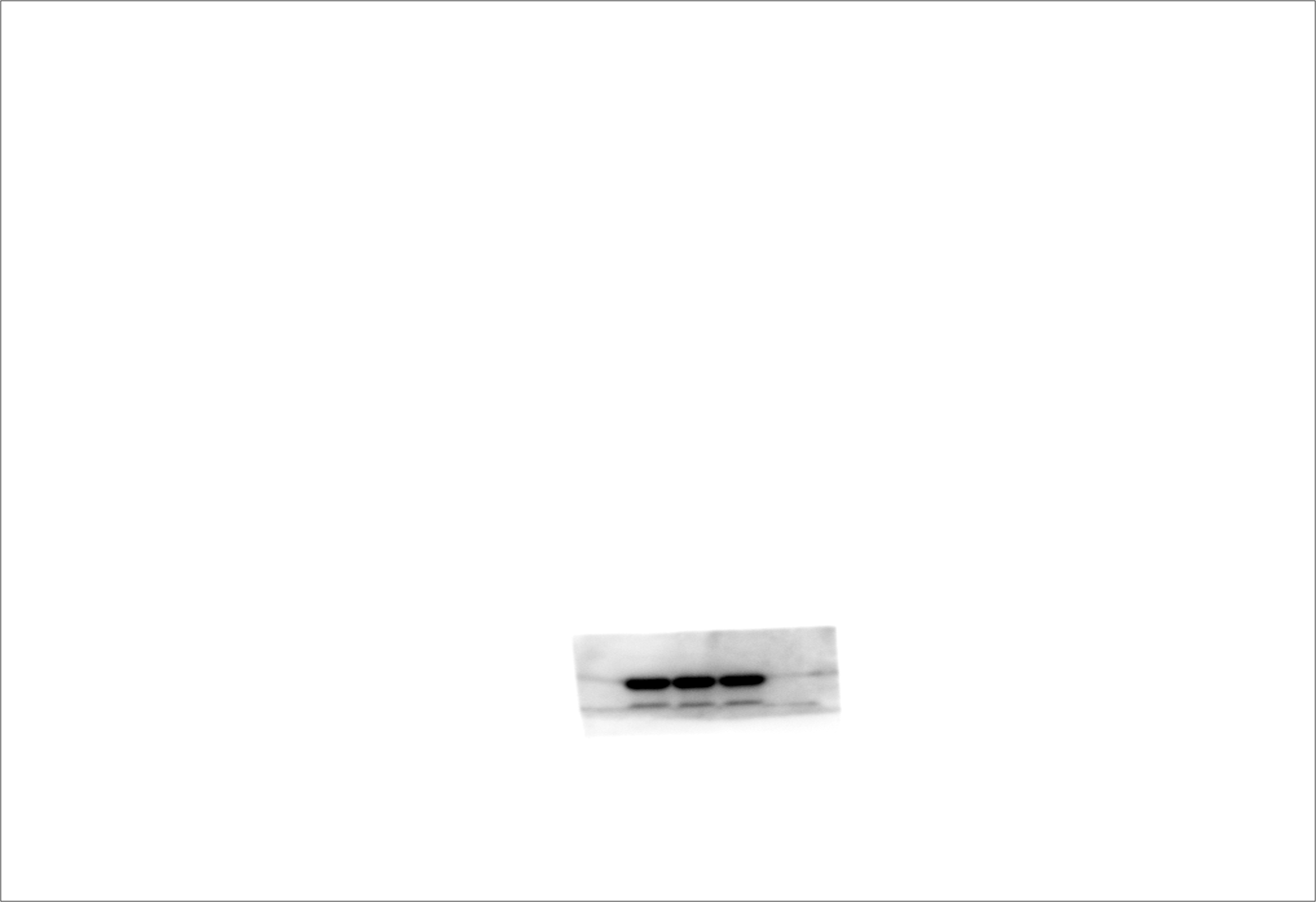


Fig 2A:


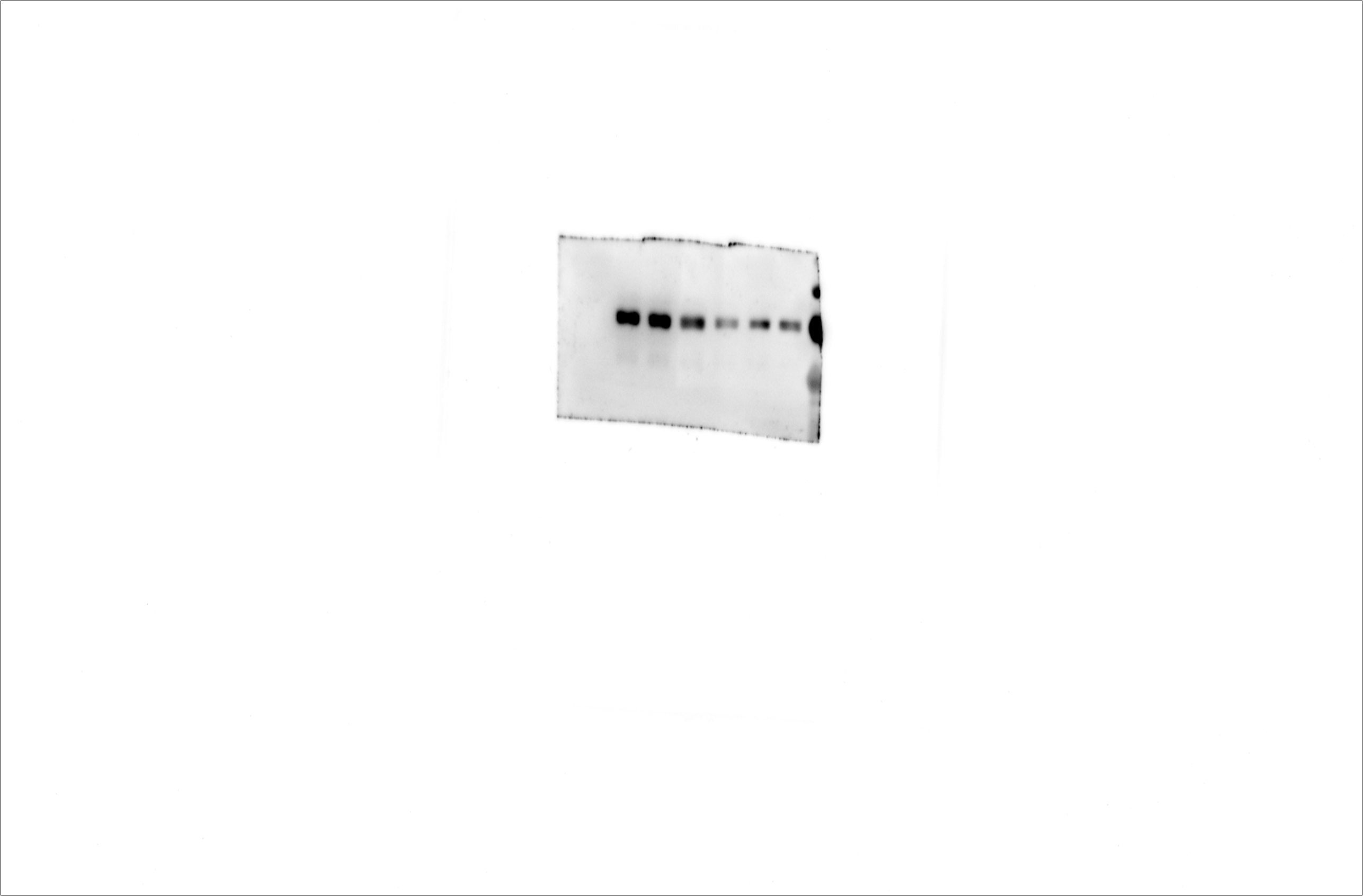

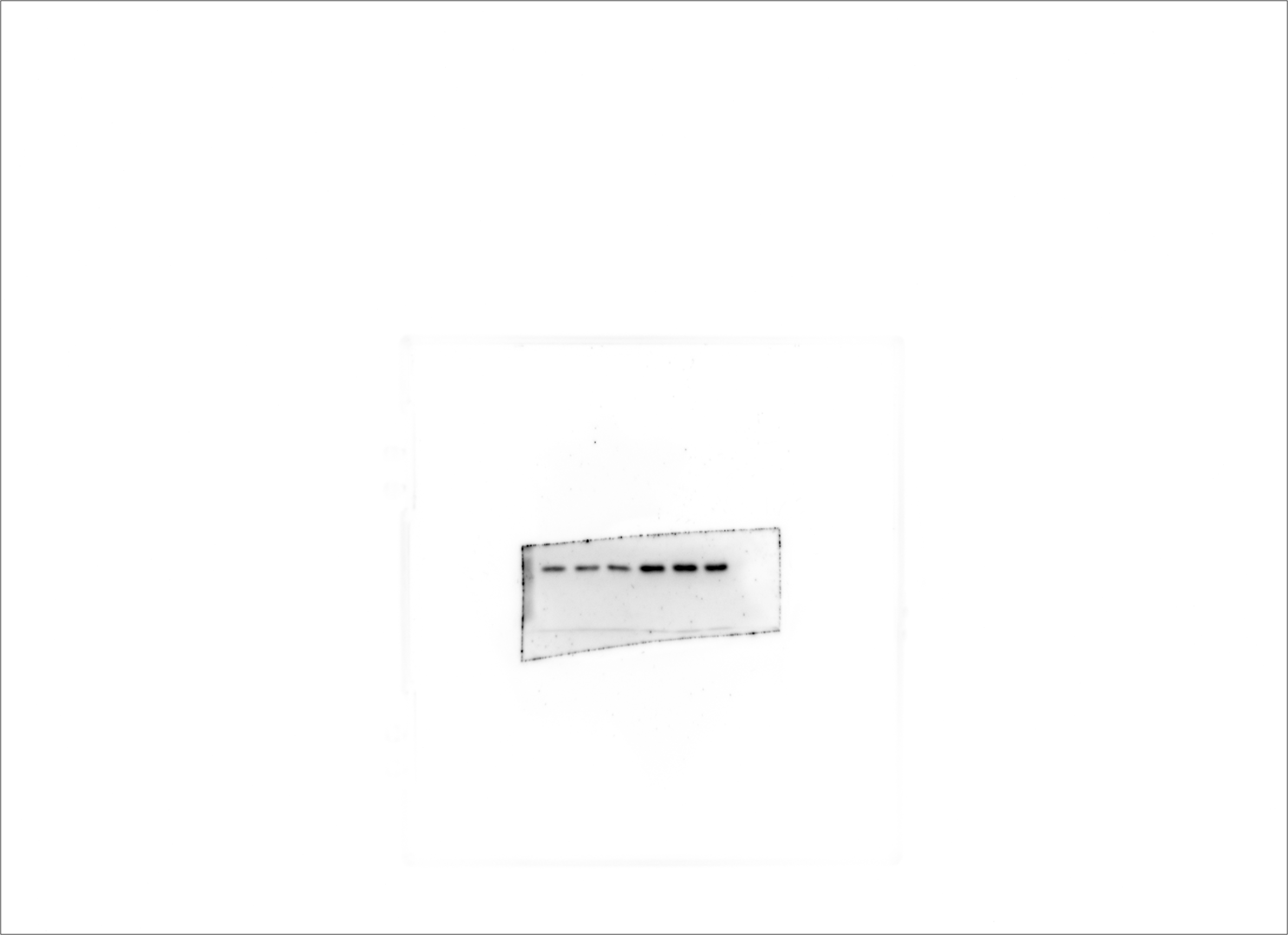

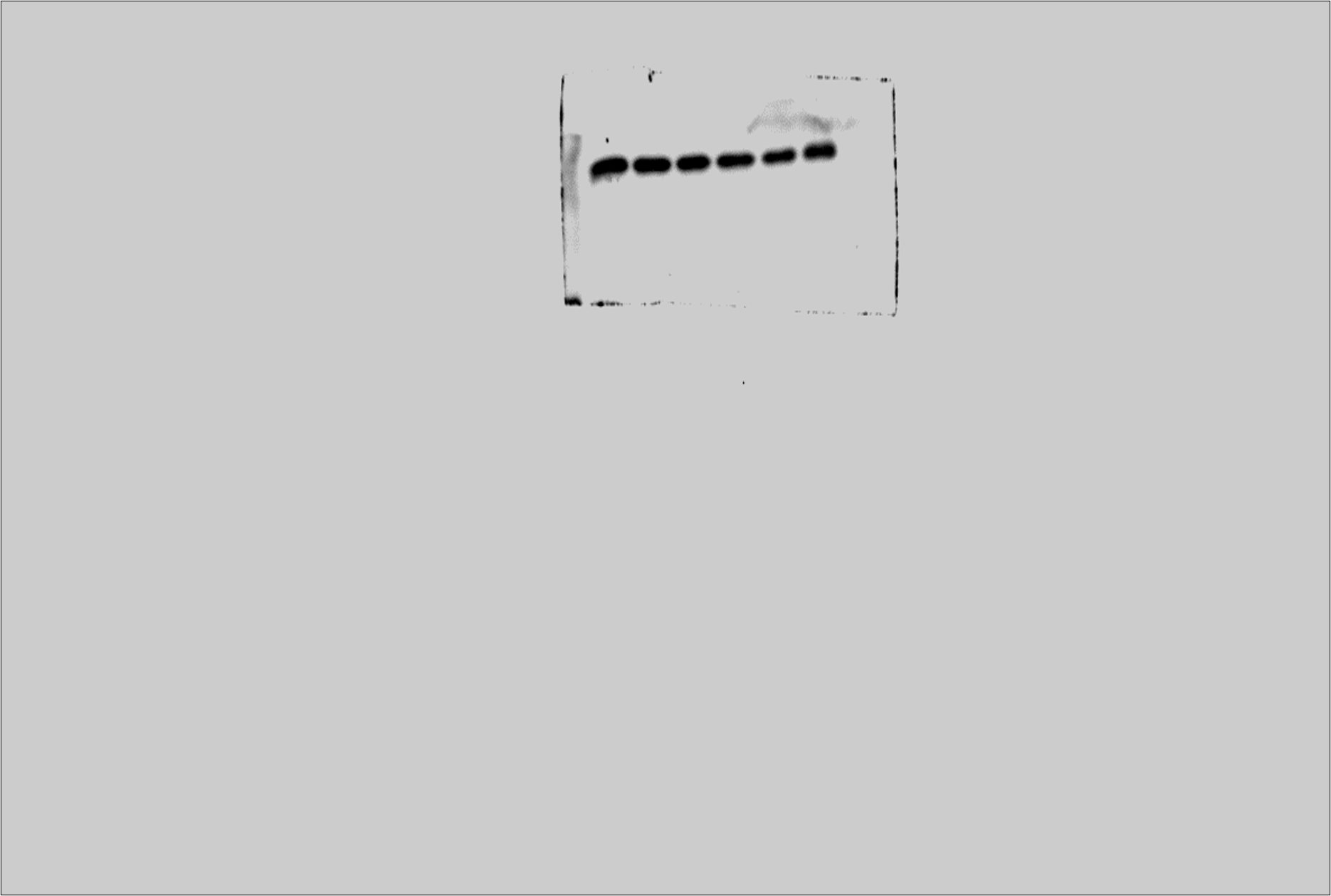


Fig 2H:


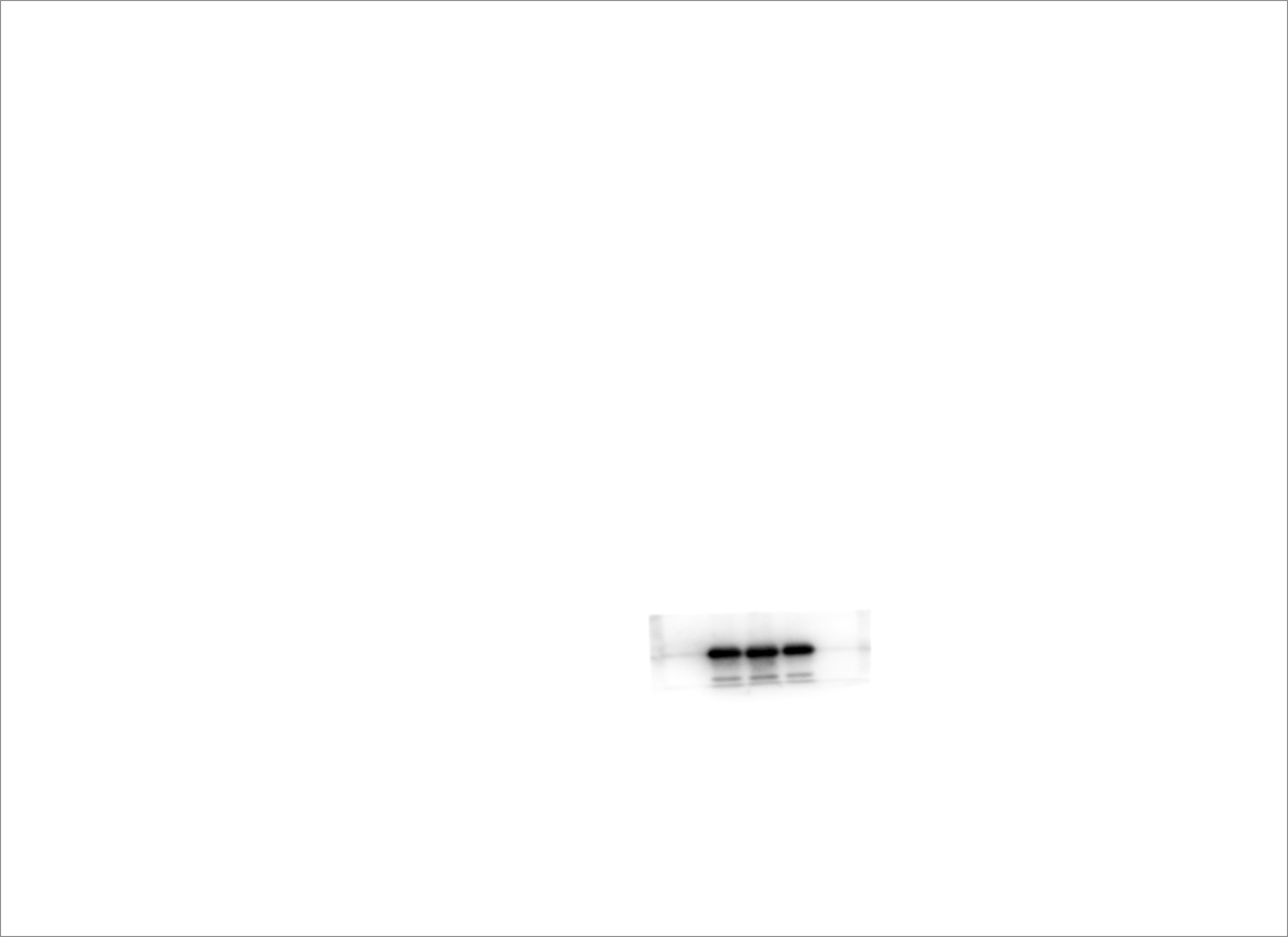

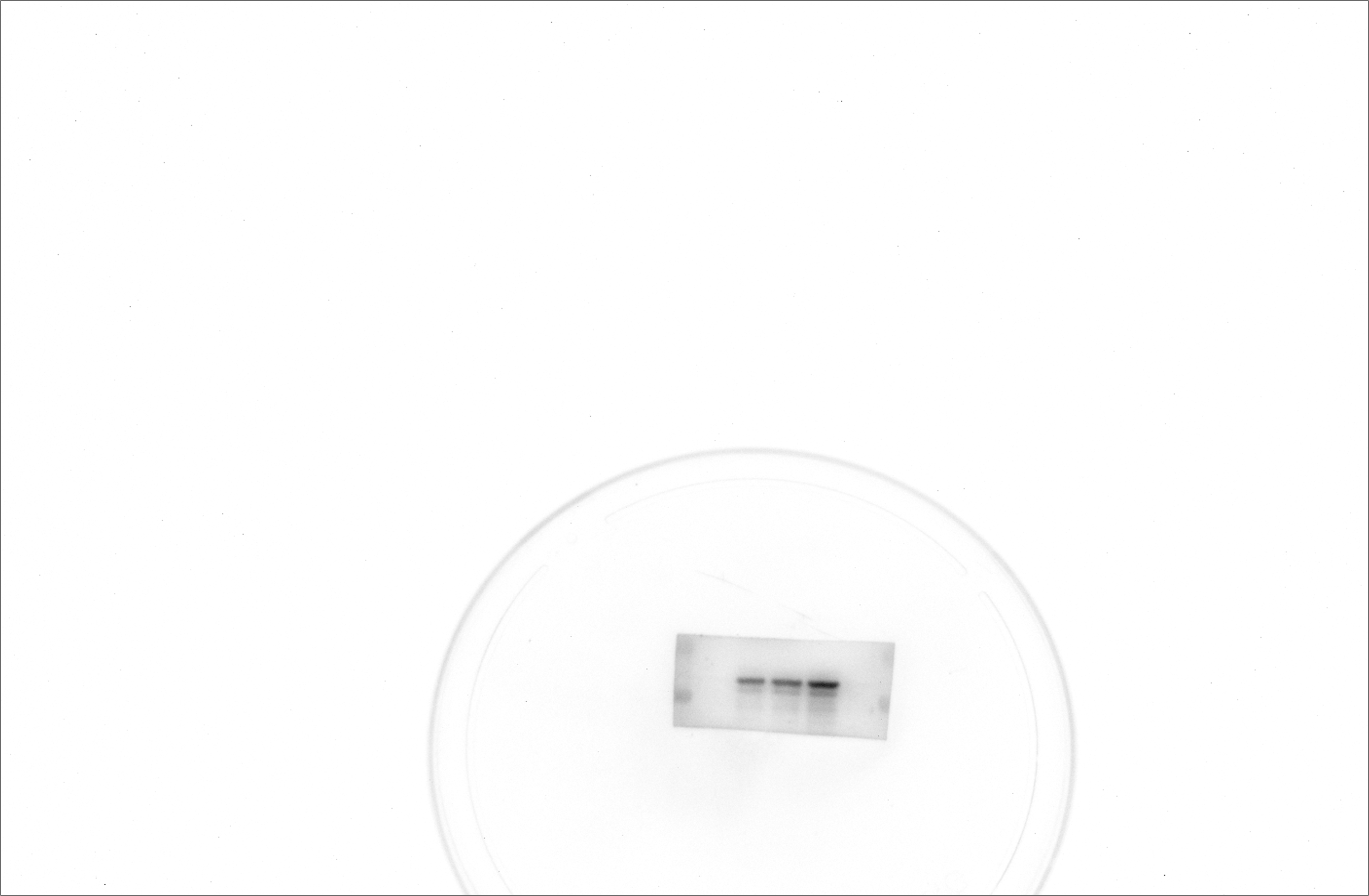

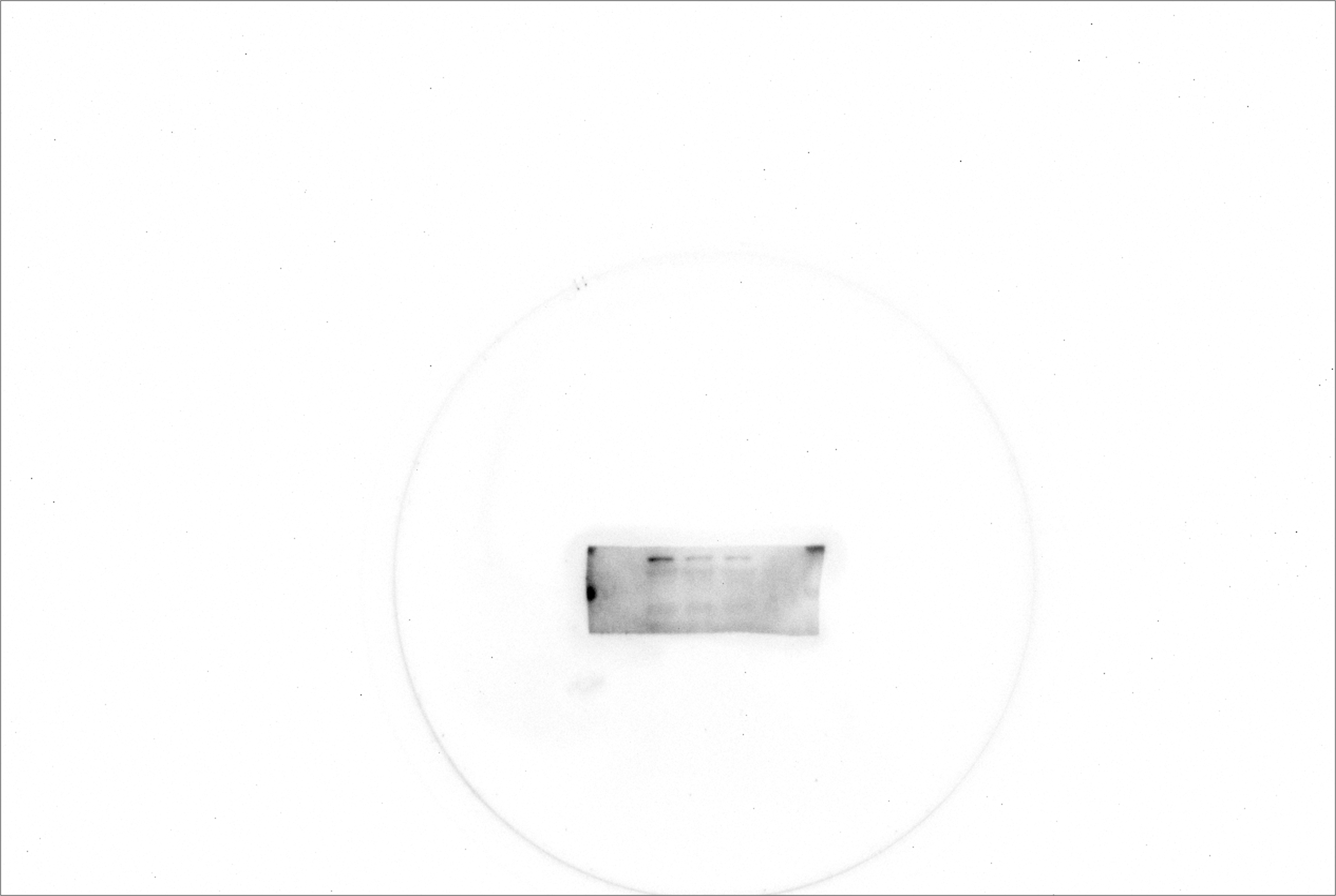

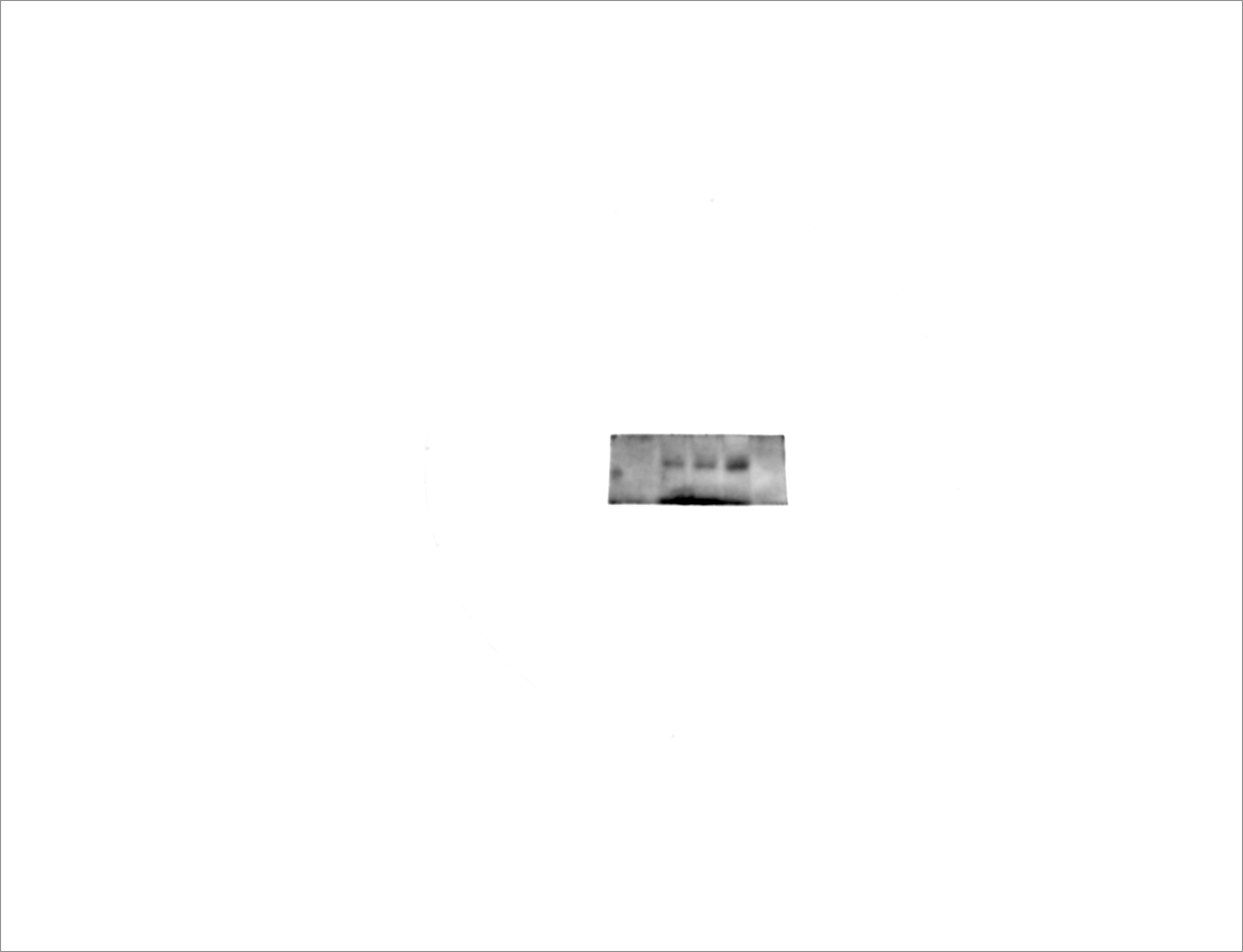


Fig 3E:


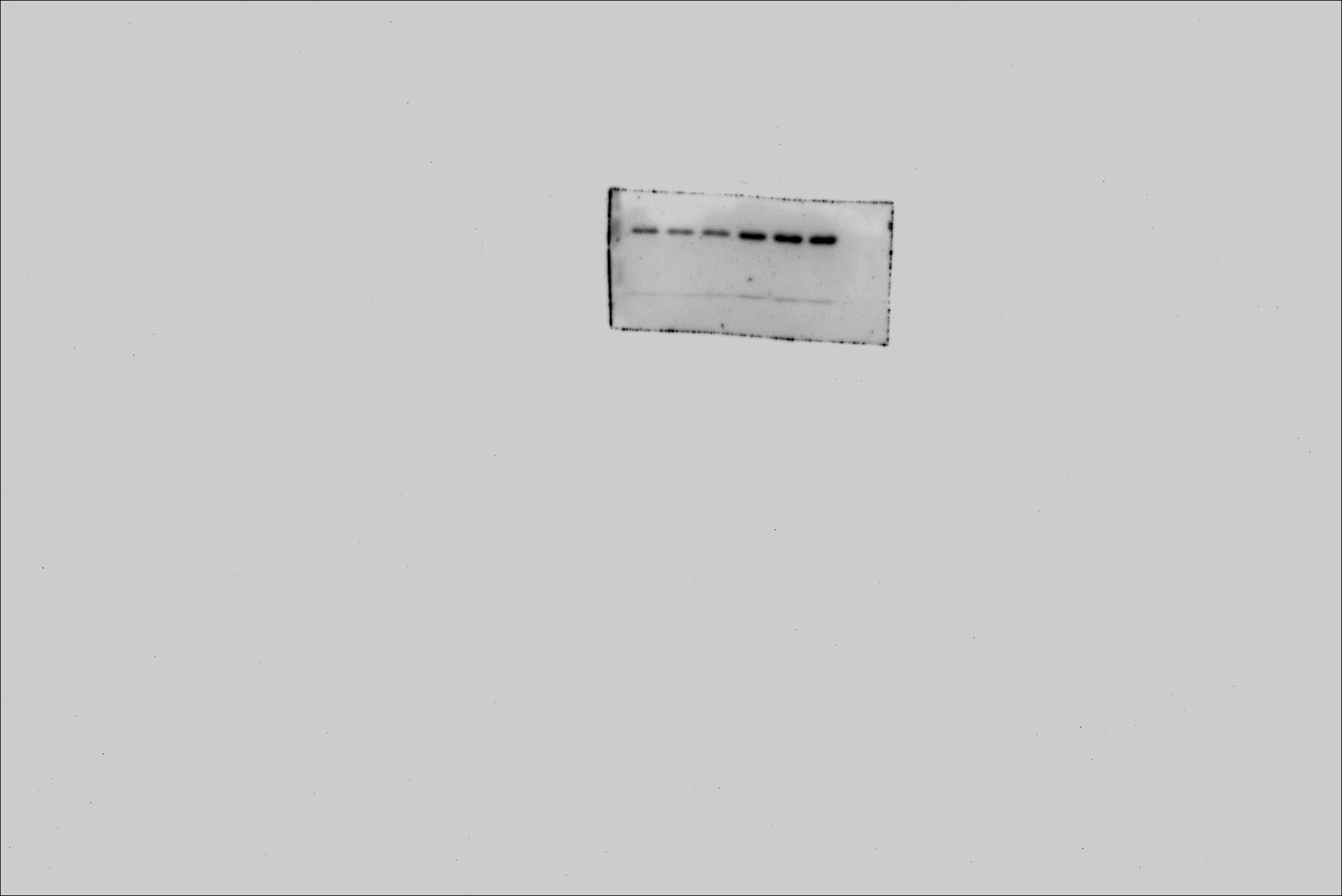

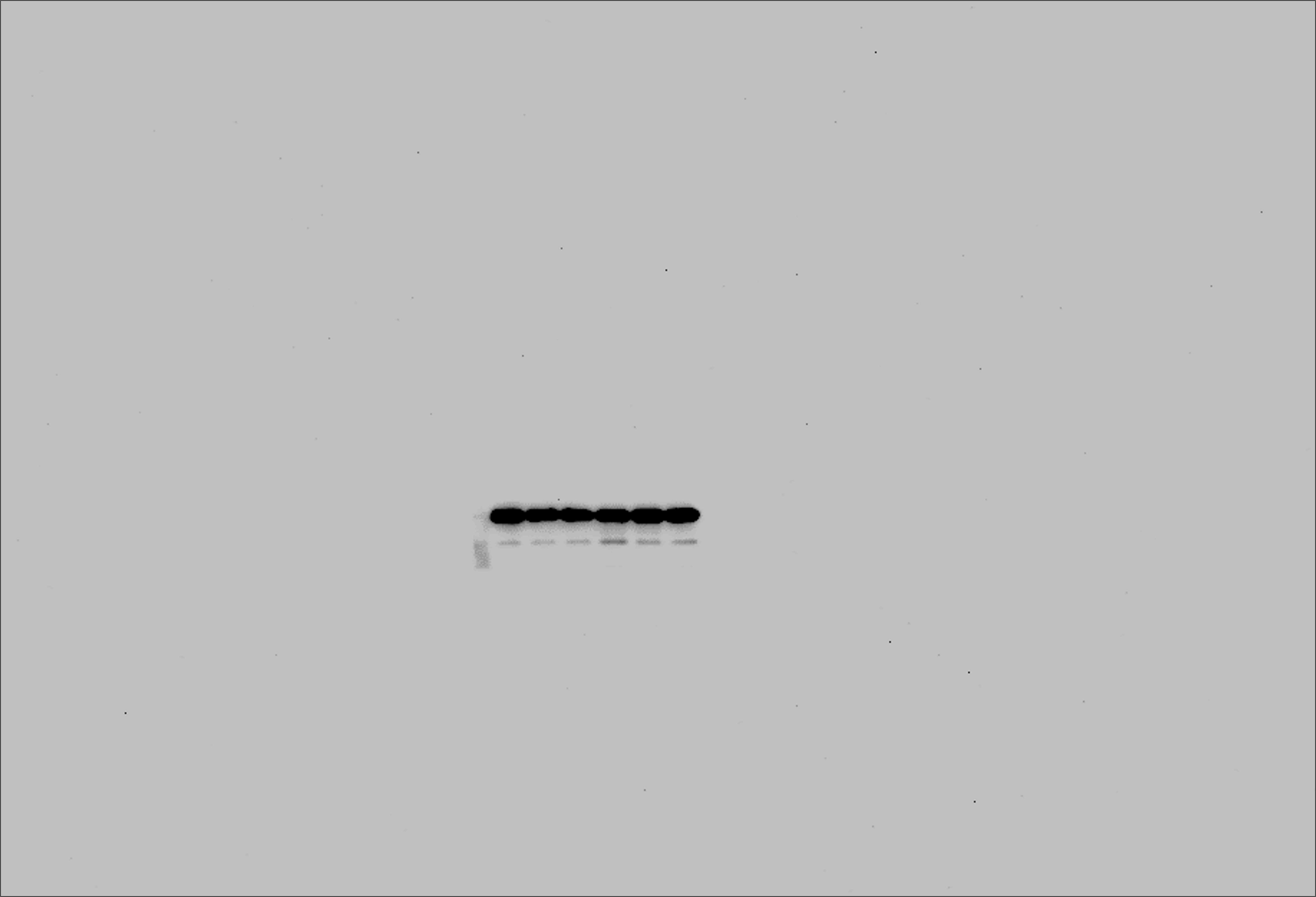

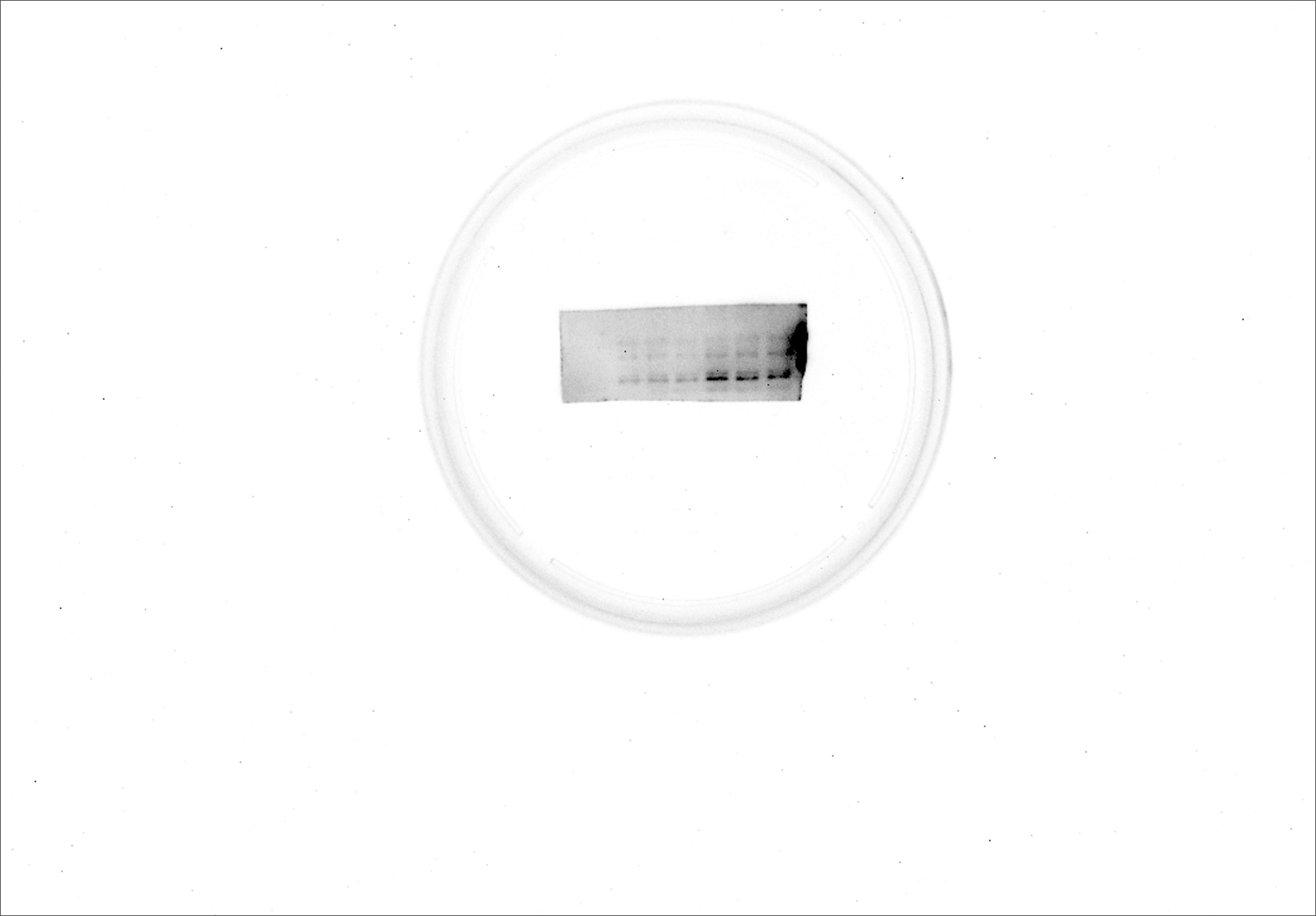


Fig 4A:


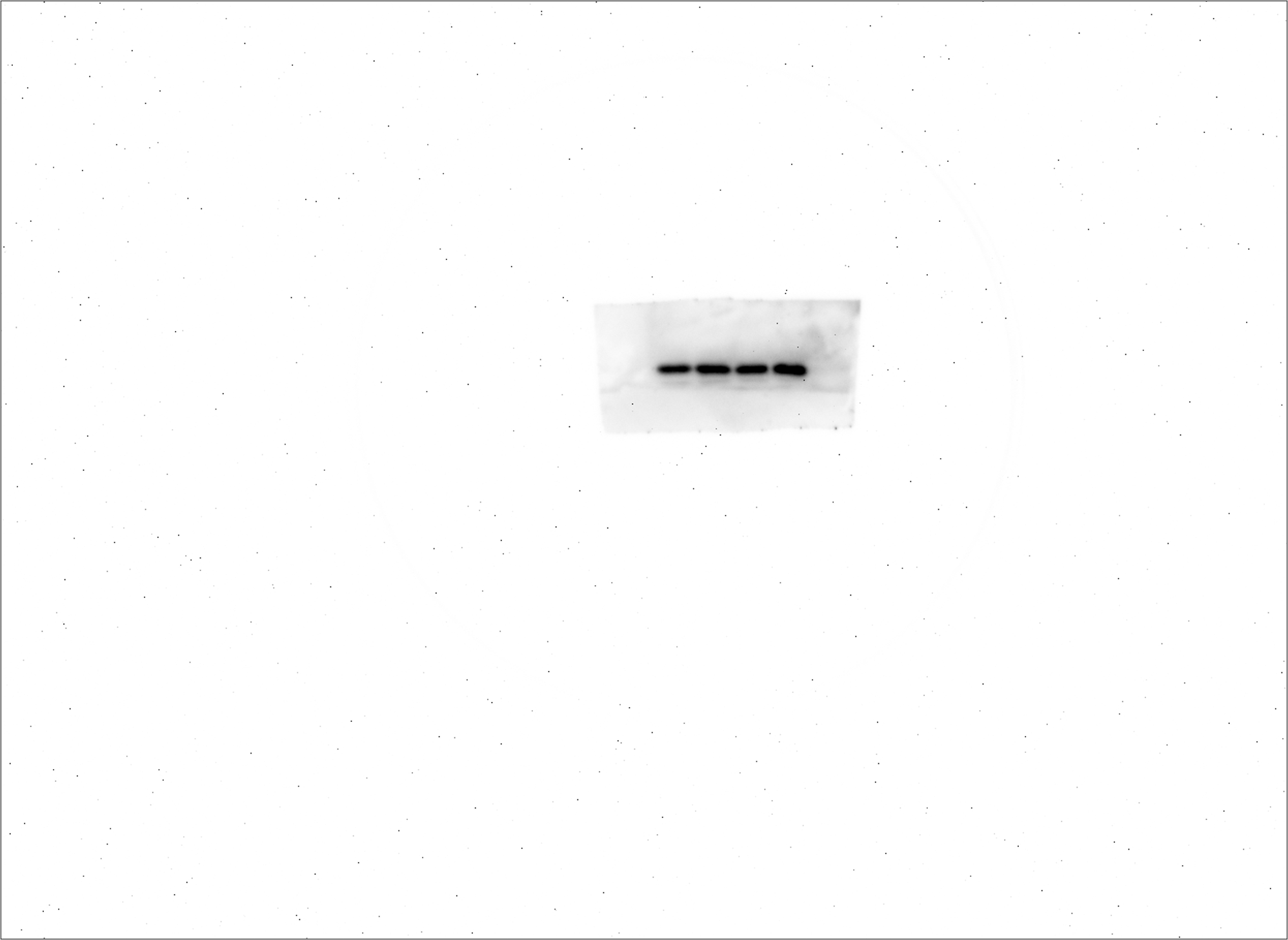

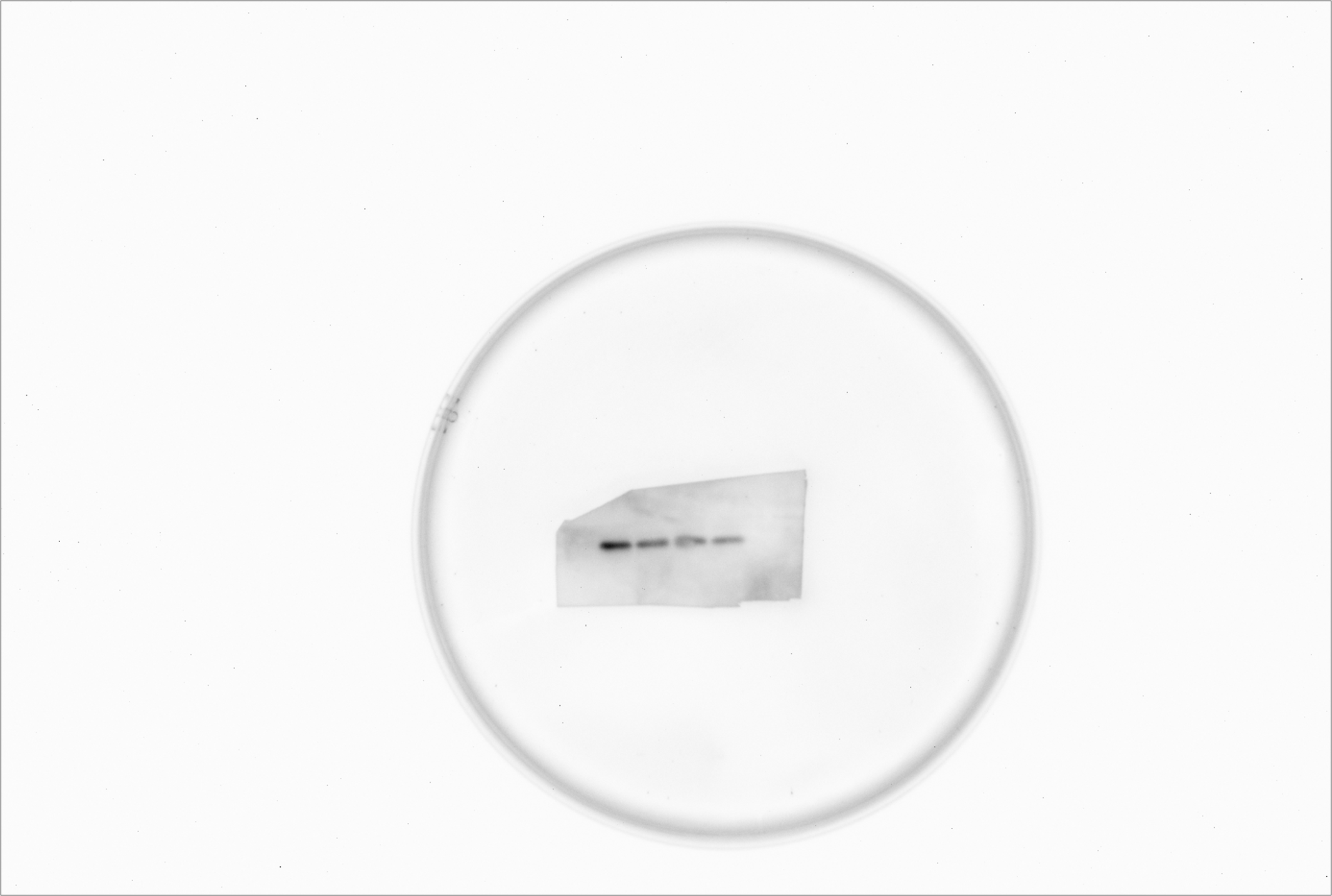


Fig 4D:


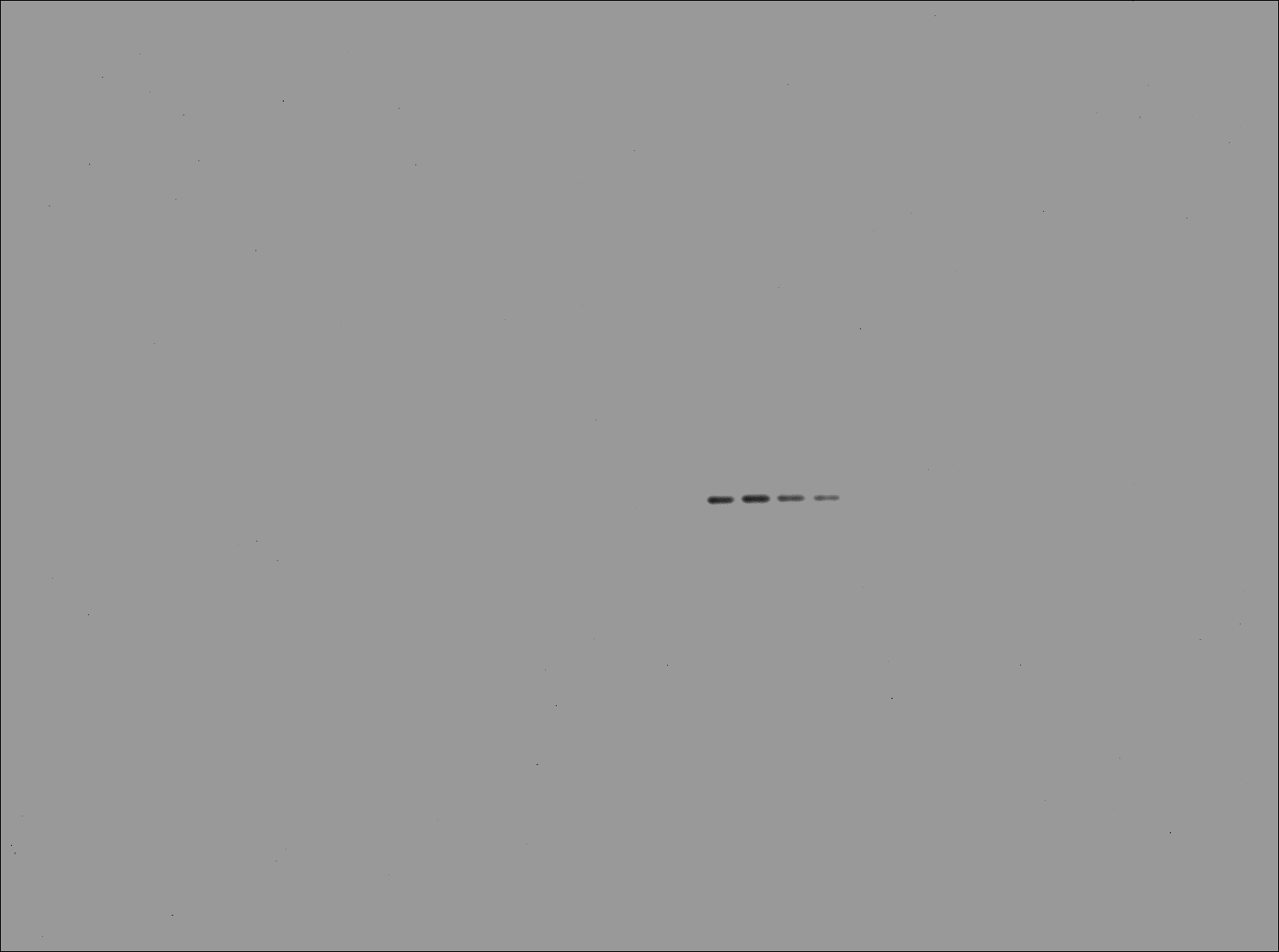

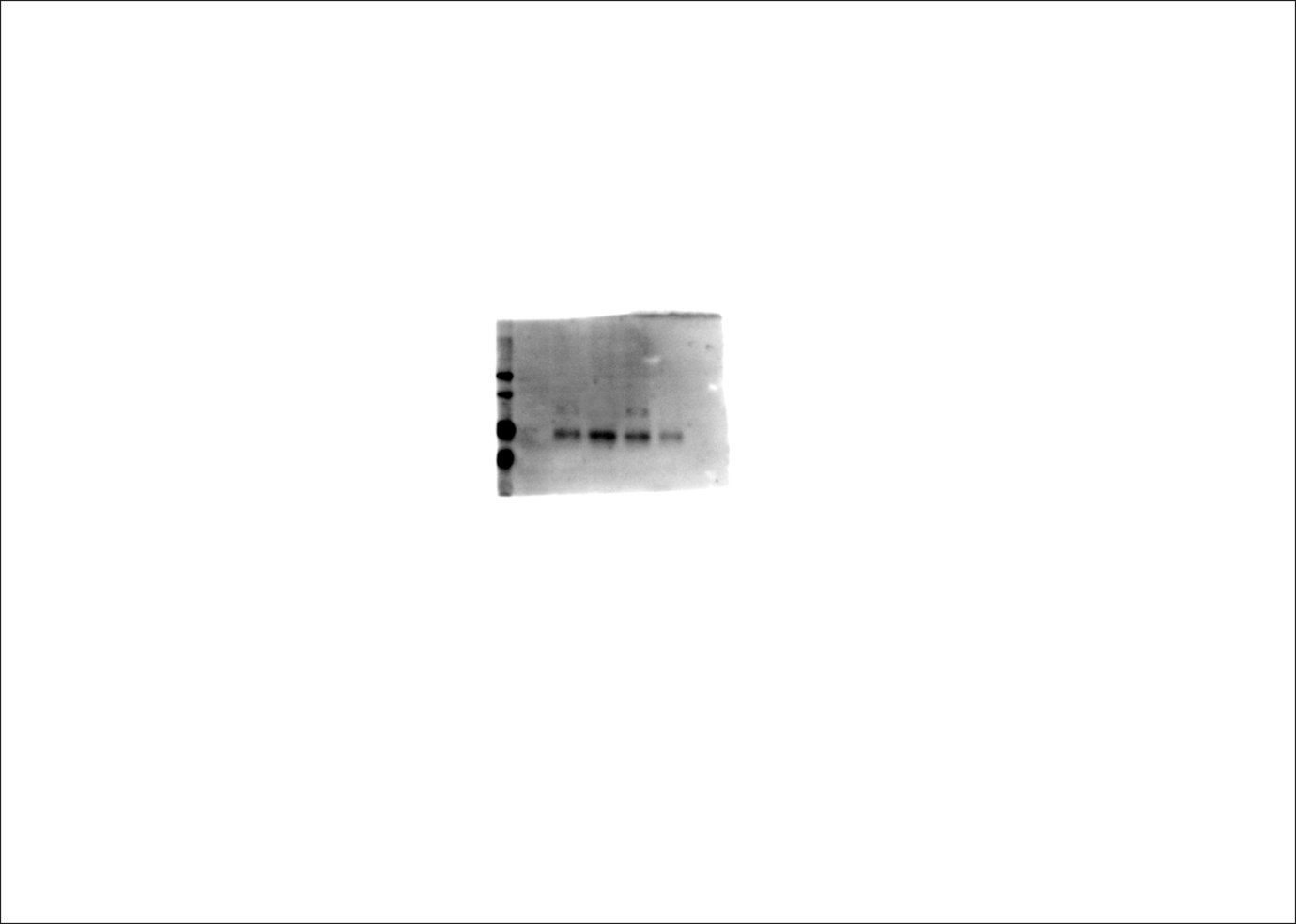

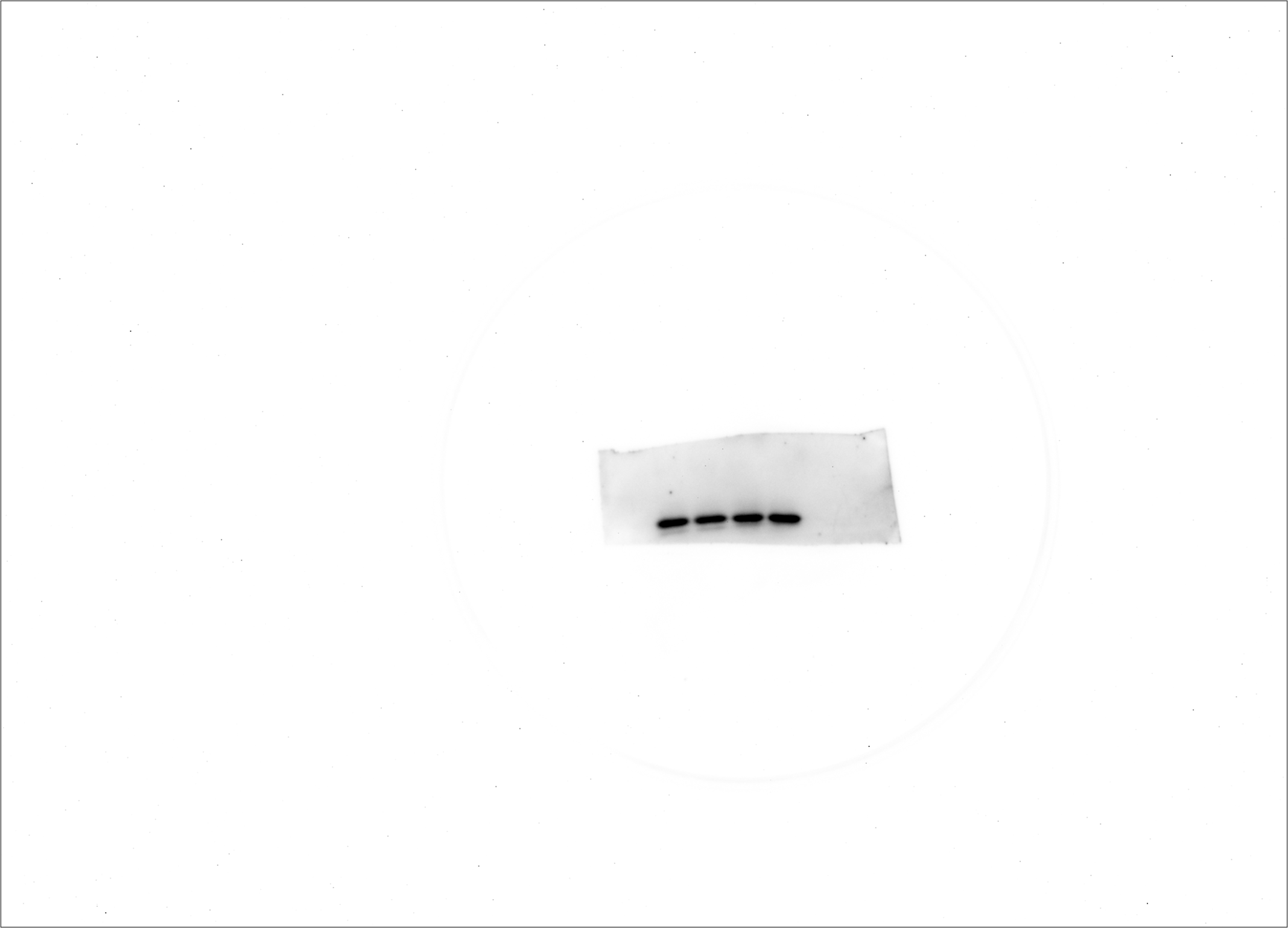

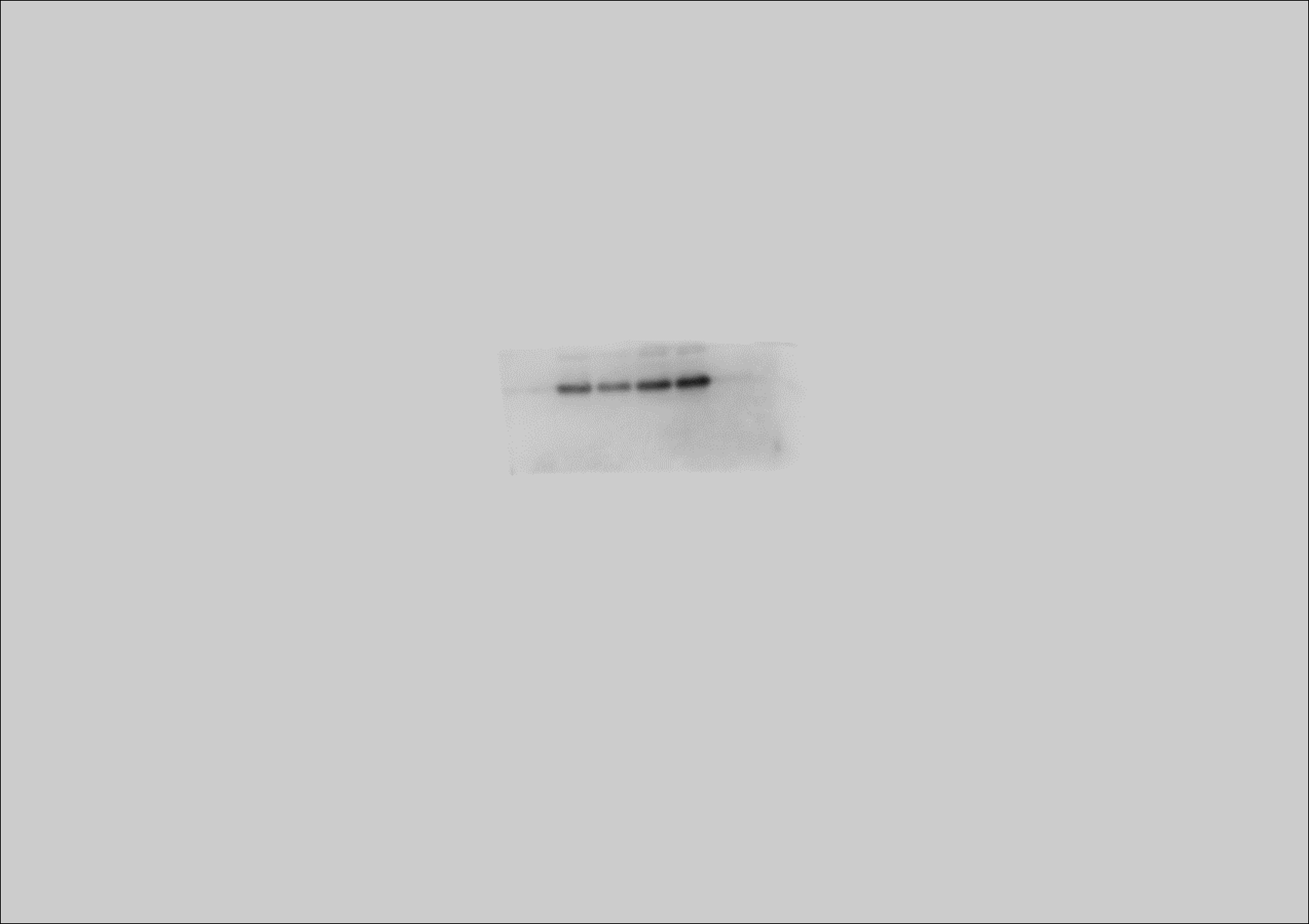

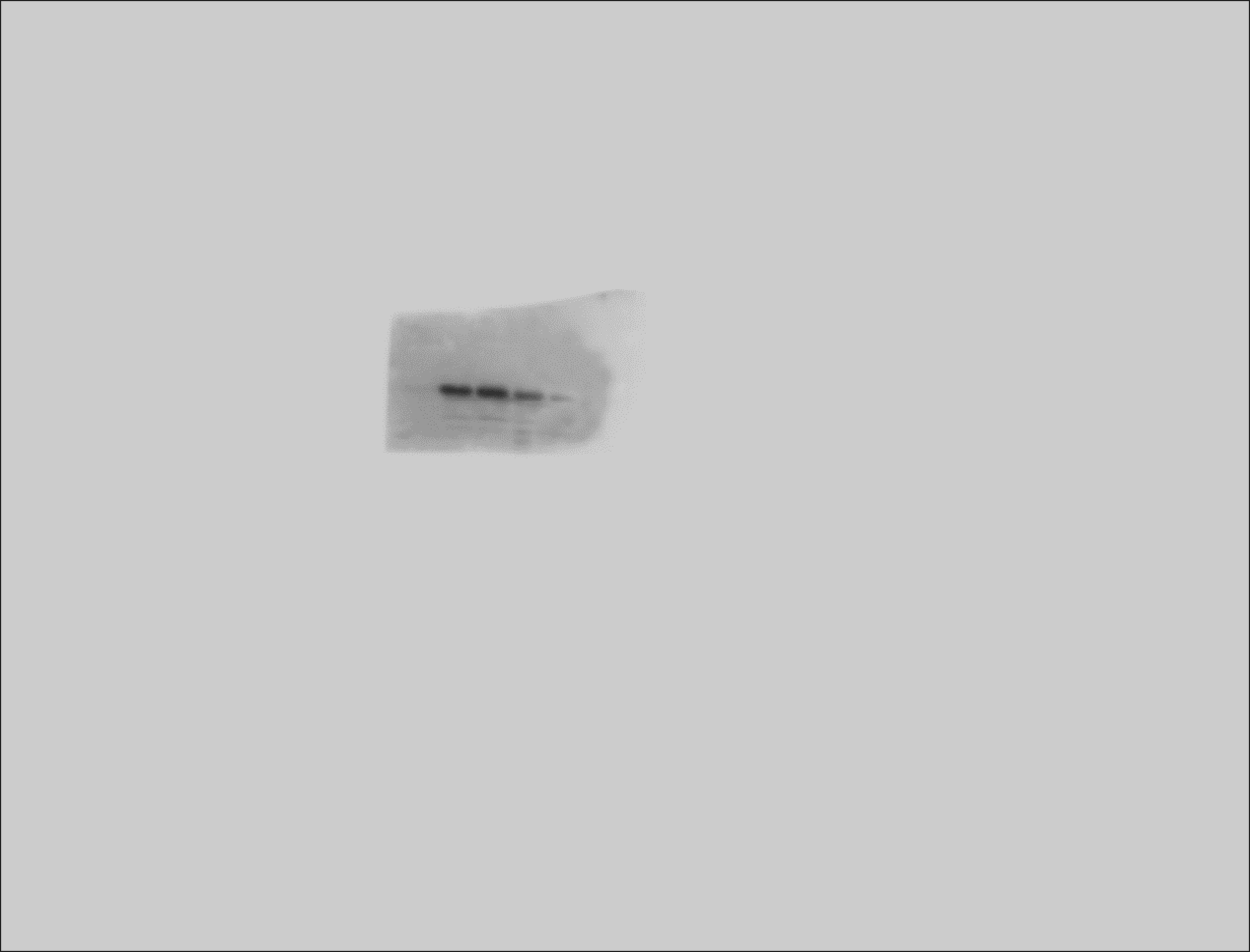


Fig 4E:


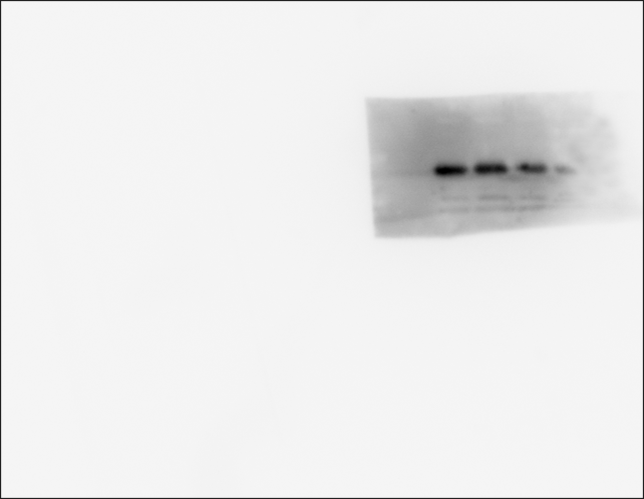

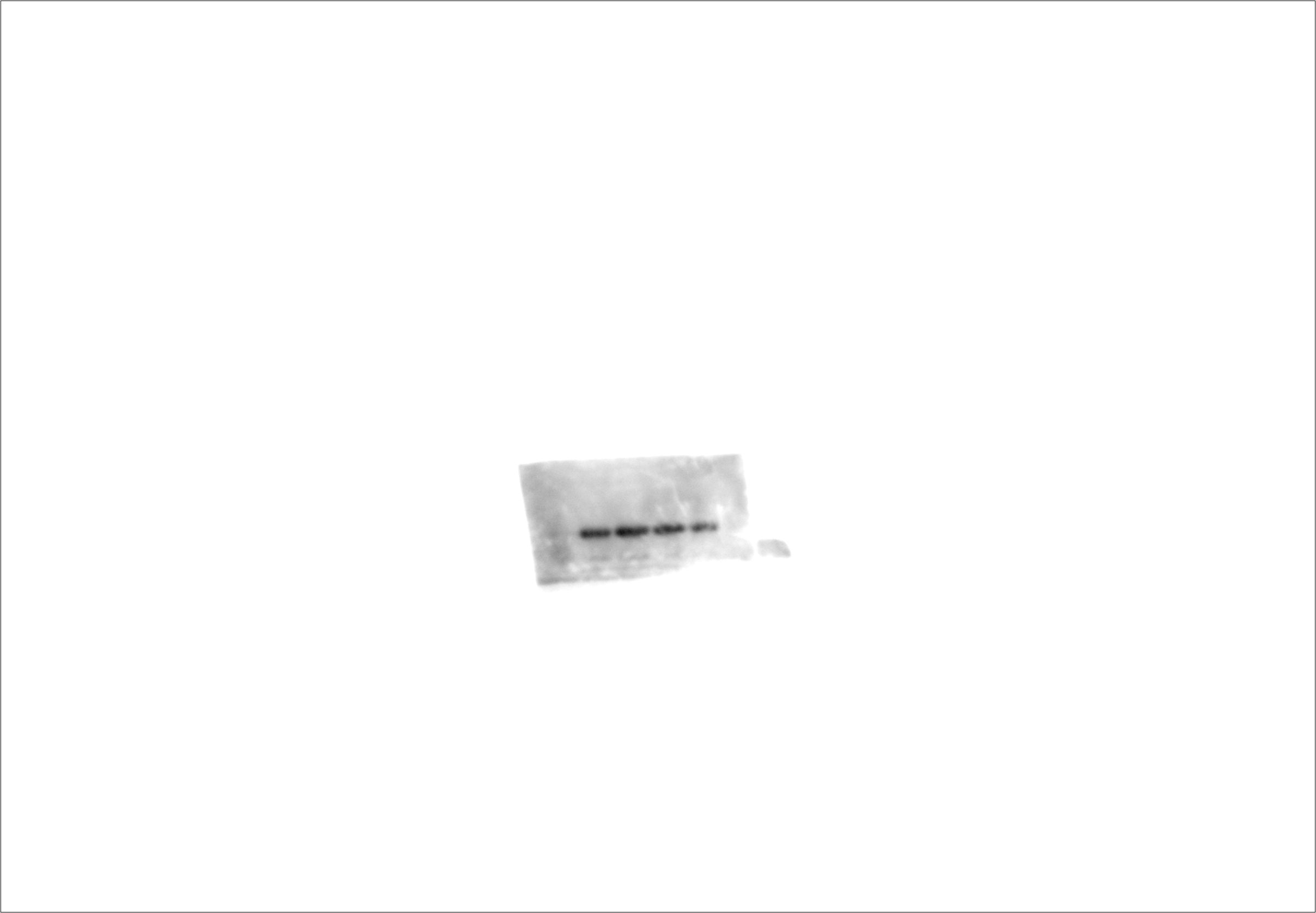

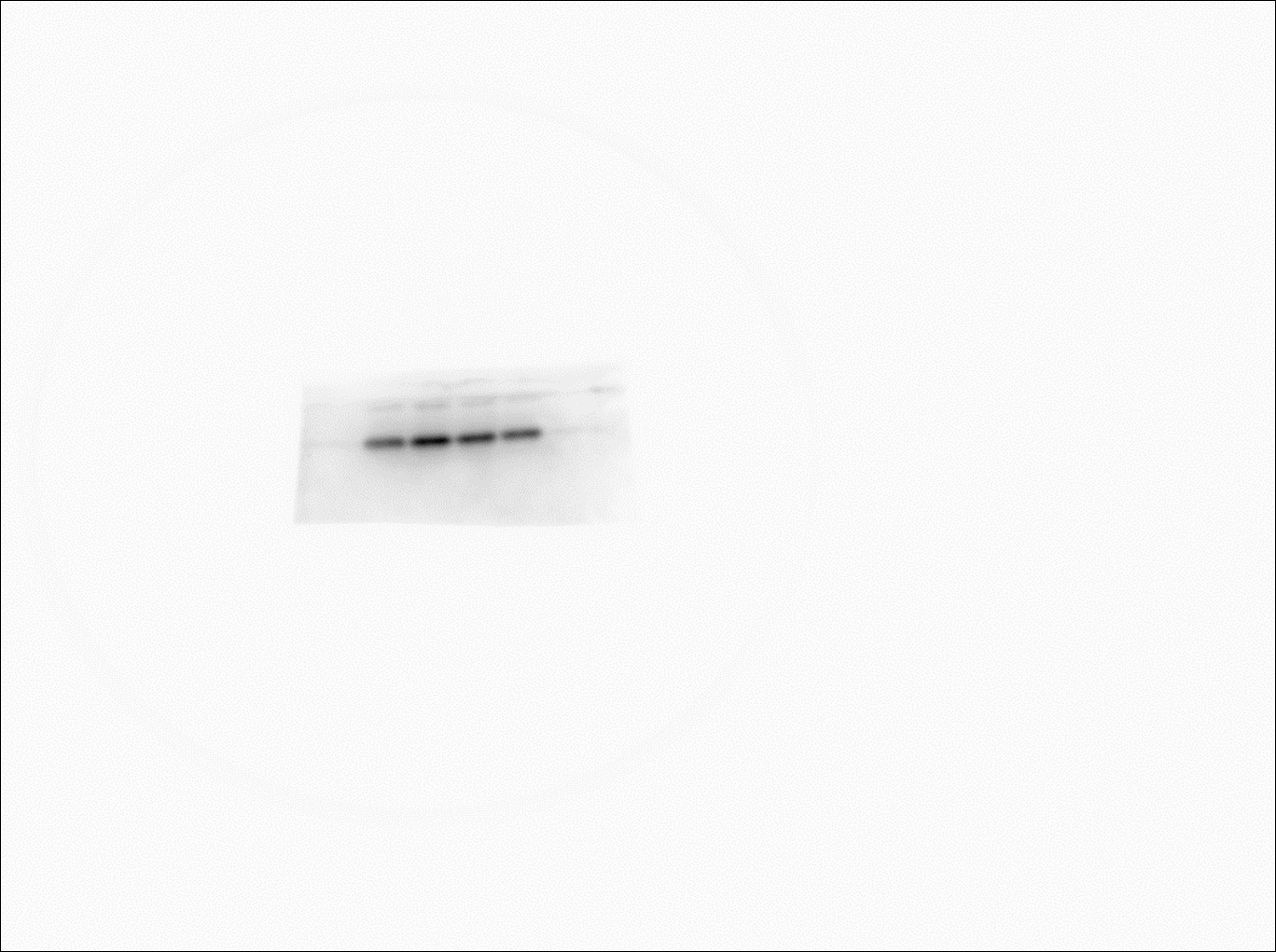

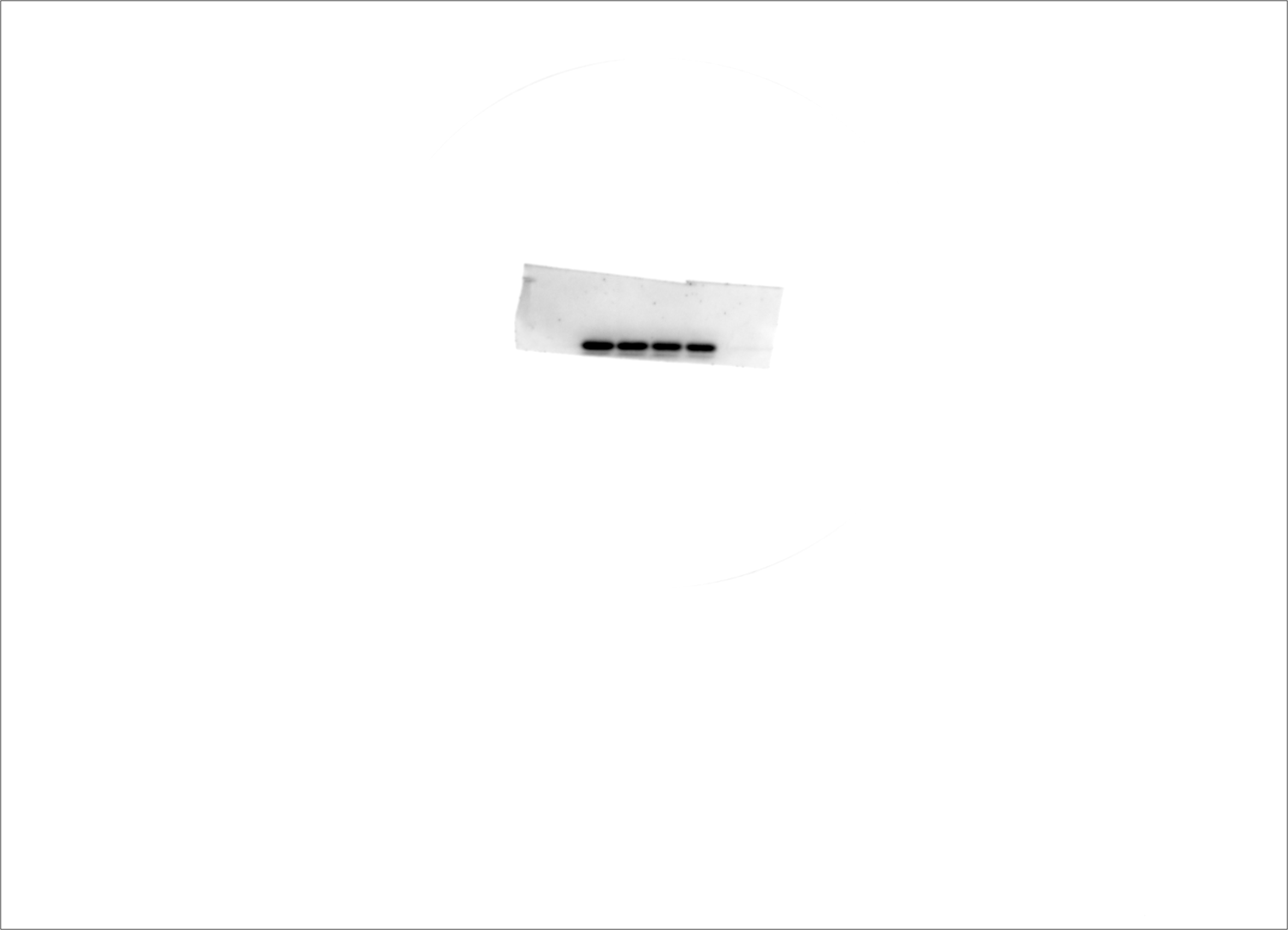


Fig 5B:


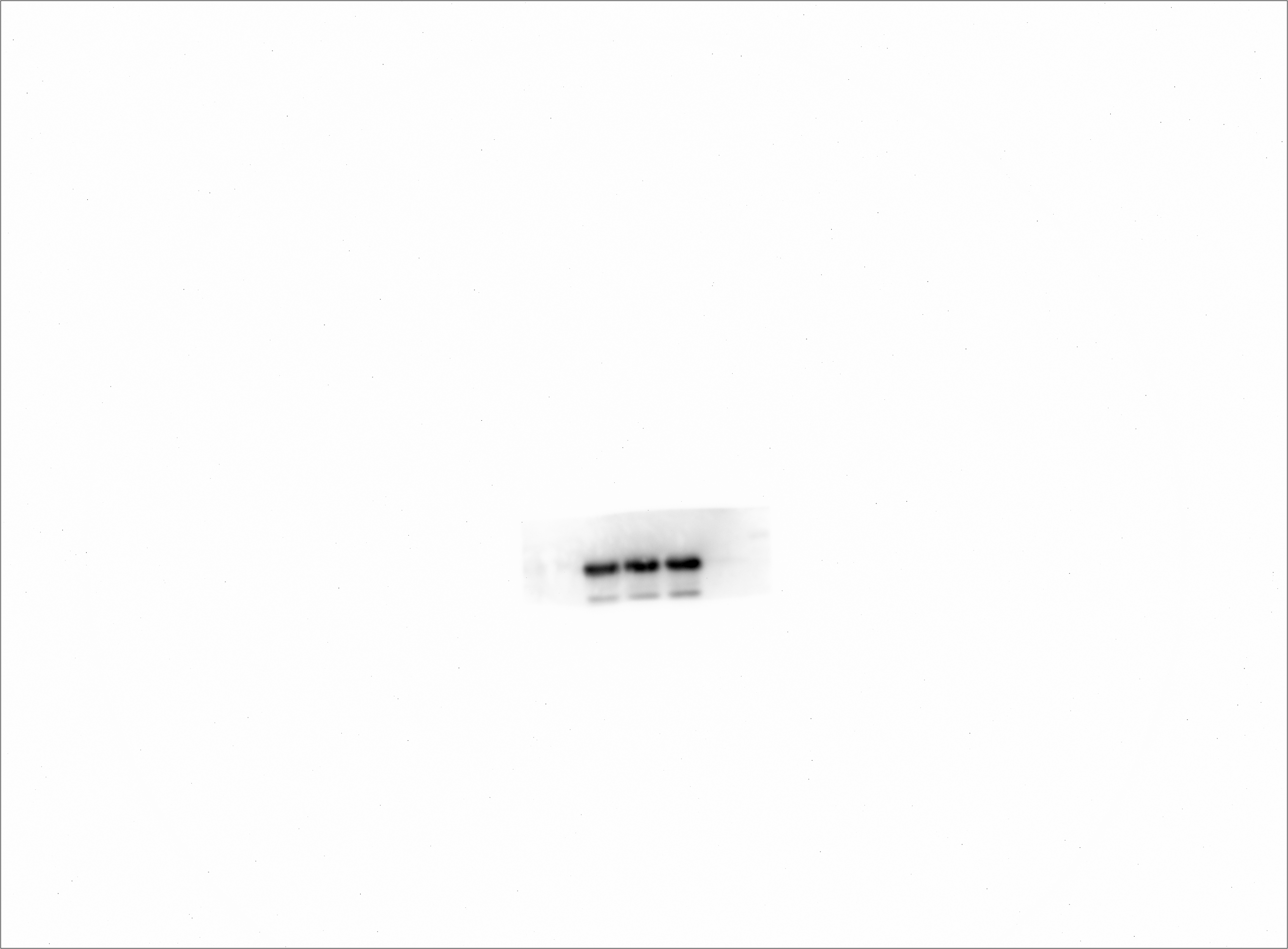

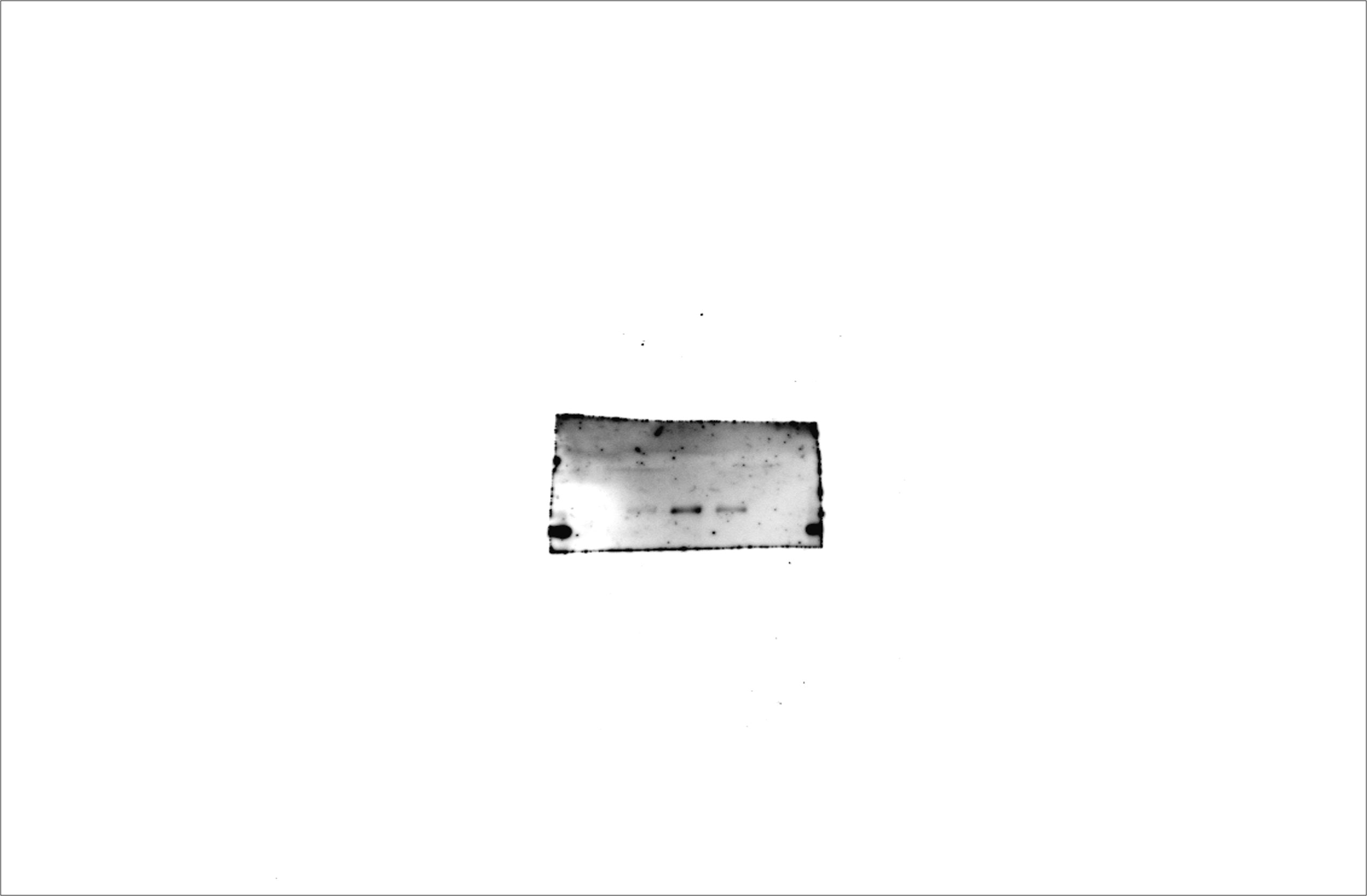

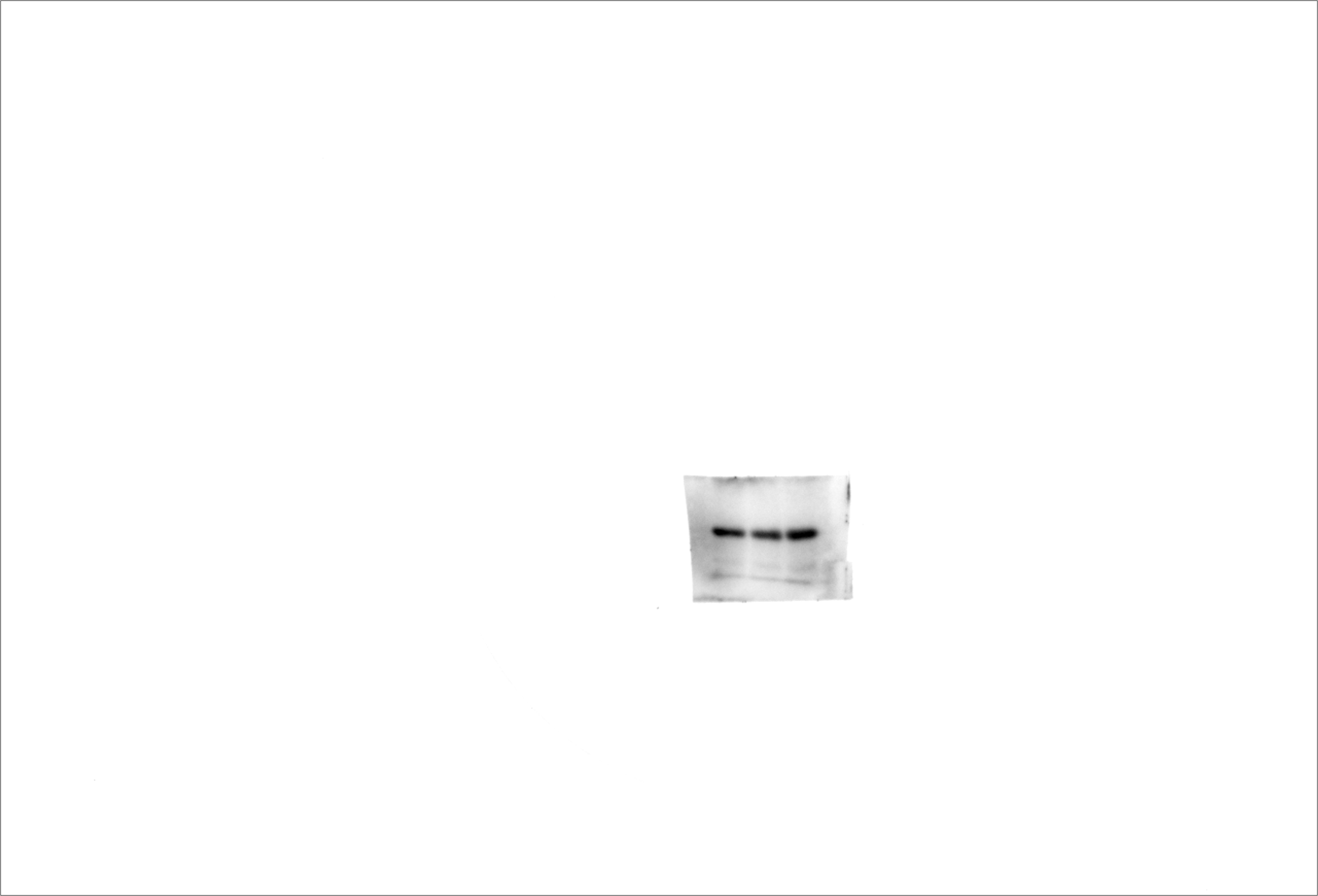

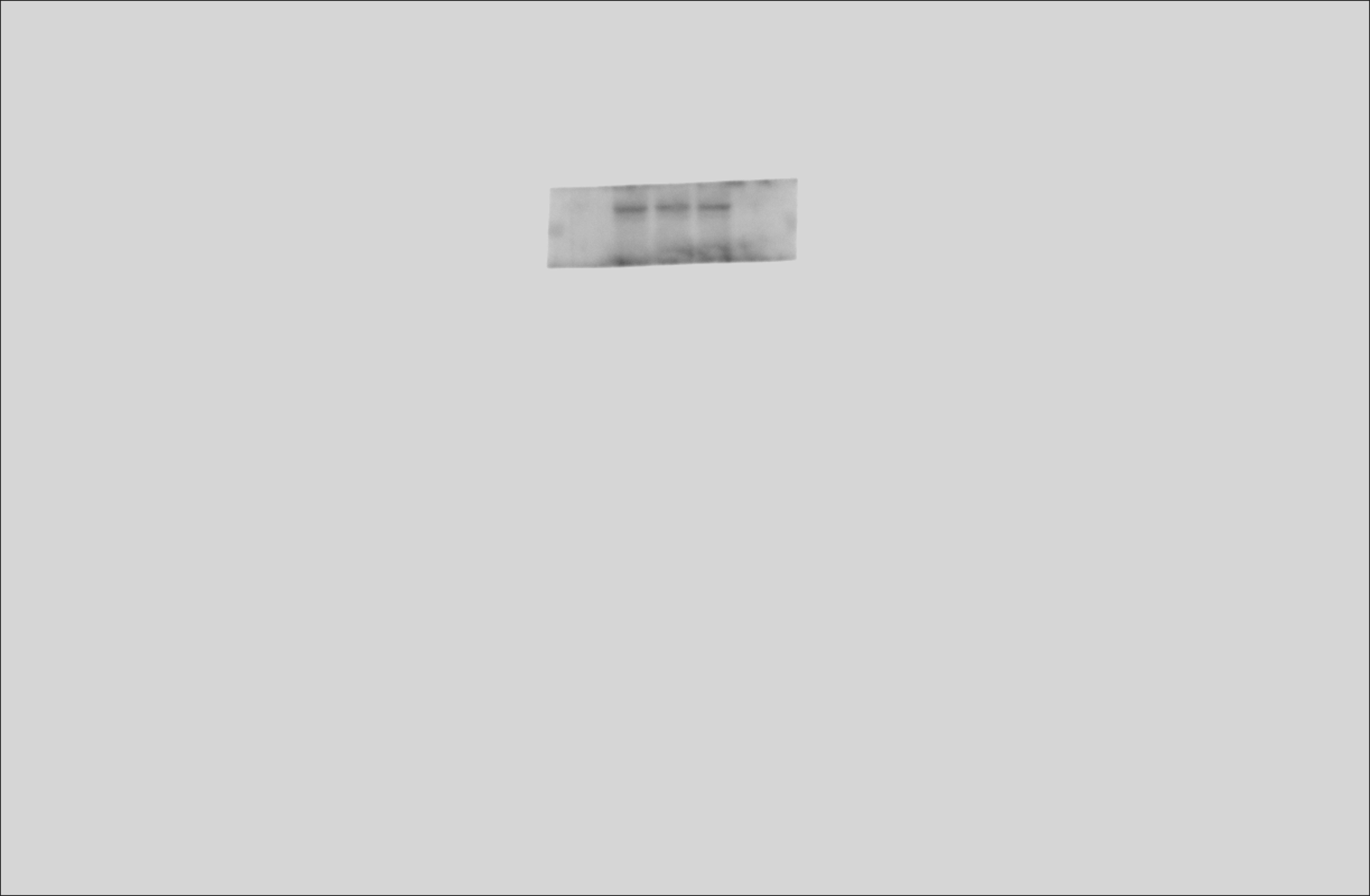


Fig 5D:


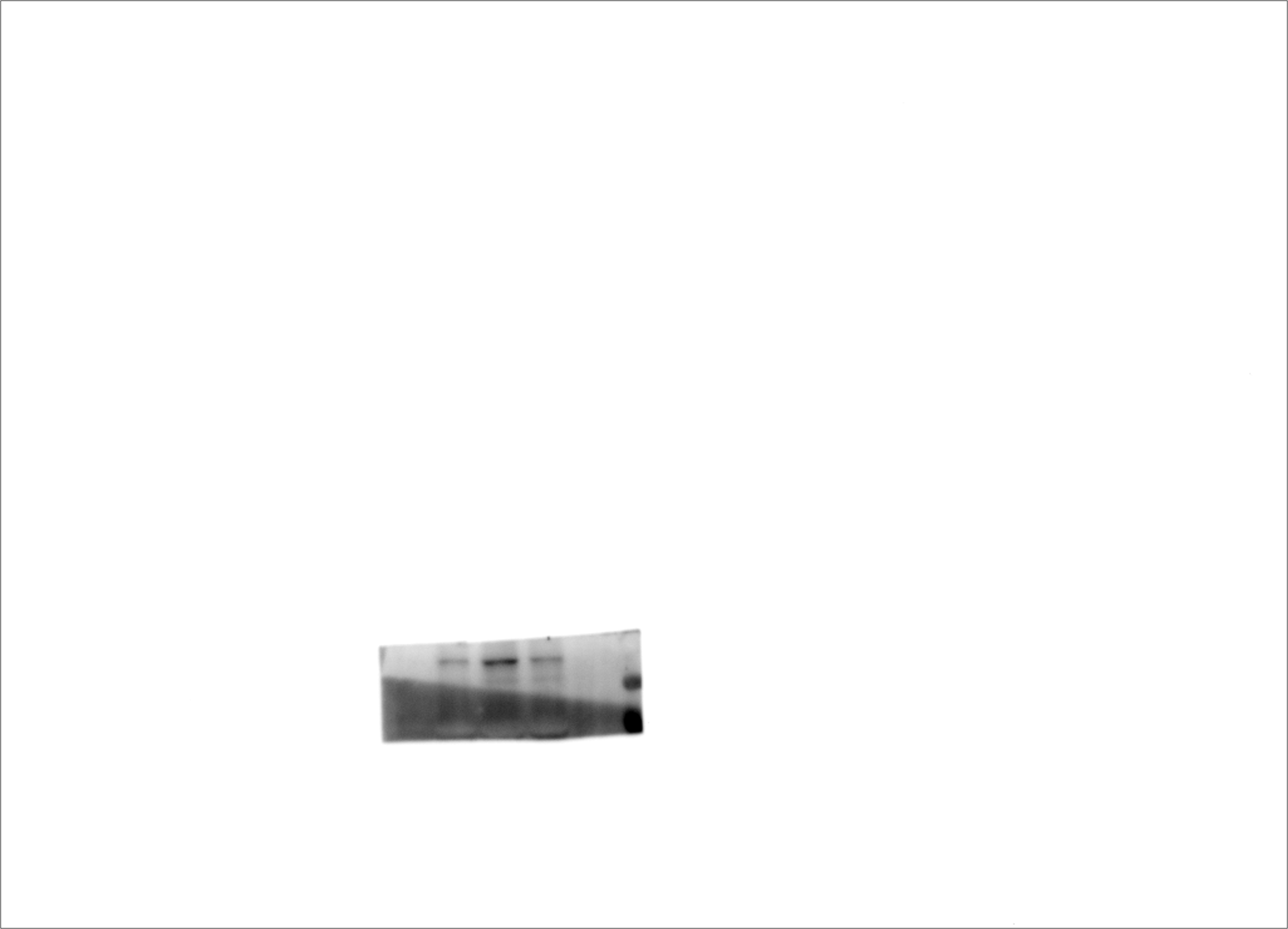

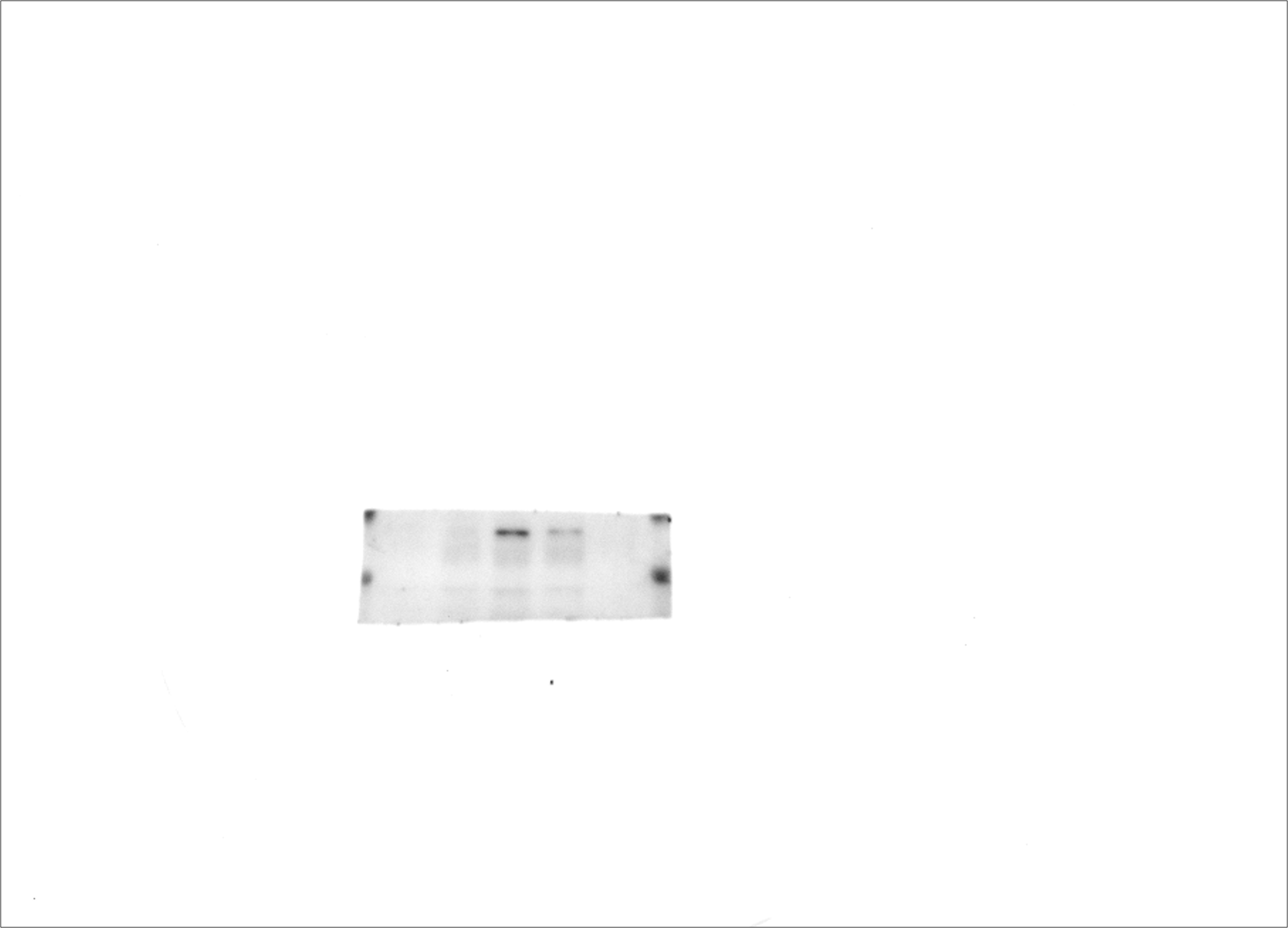

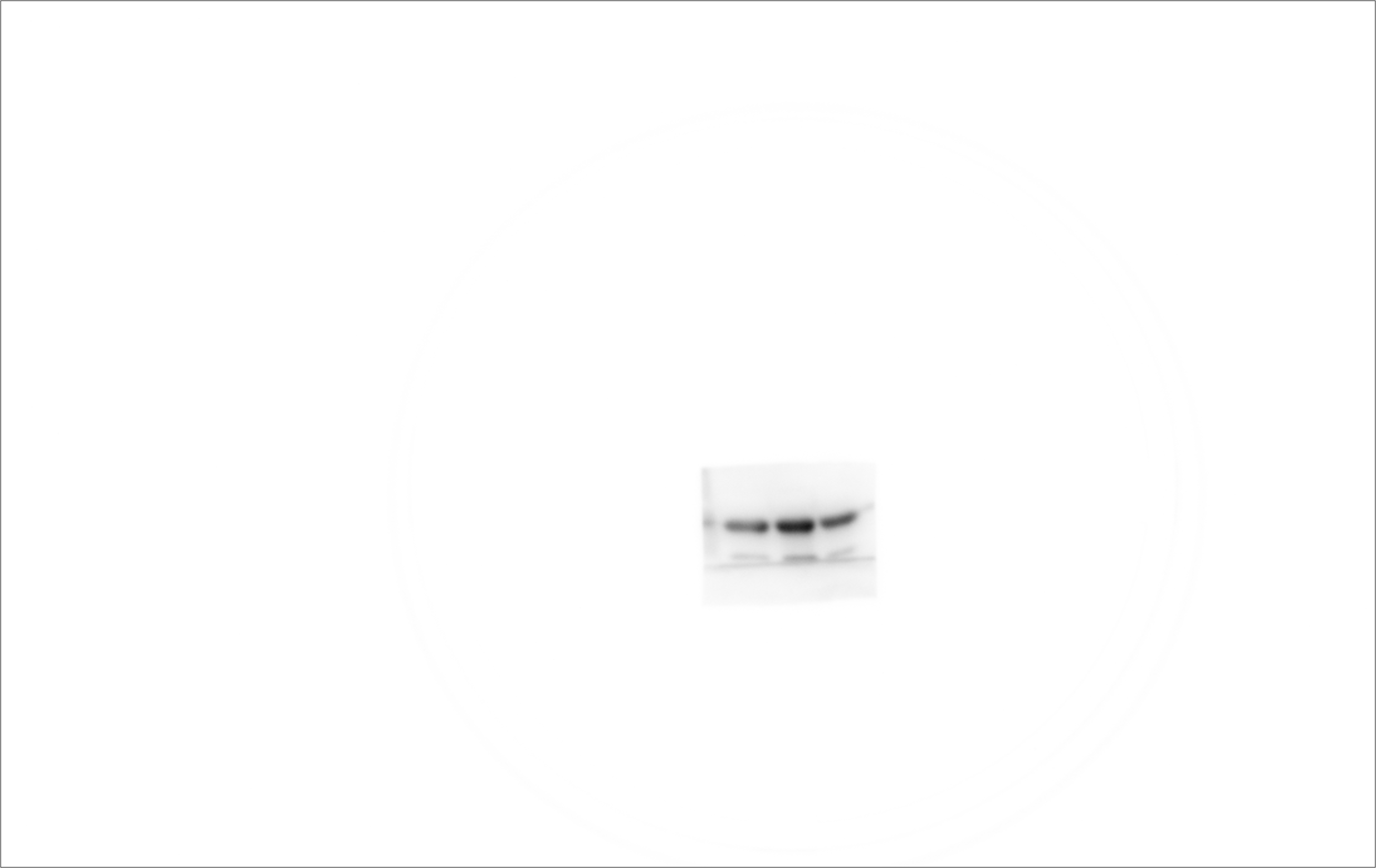

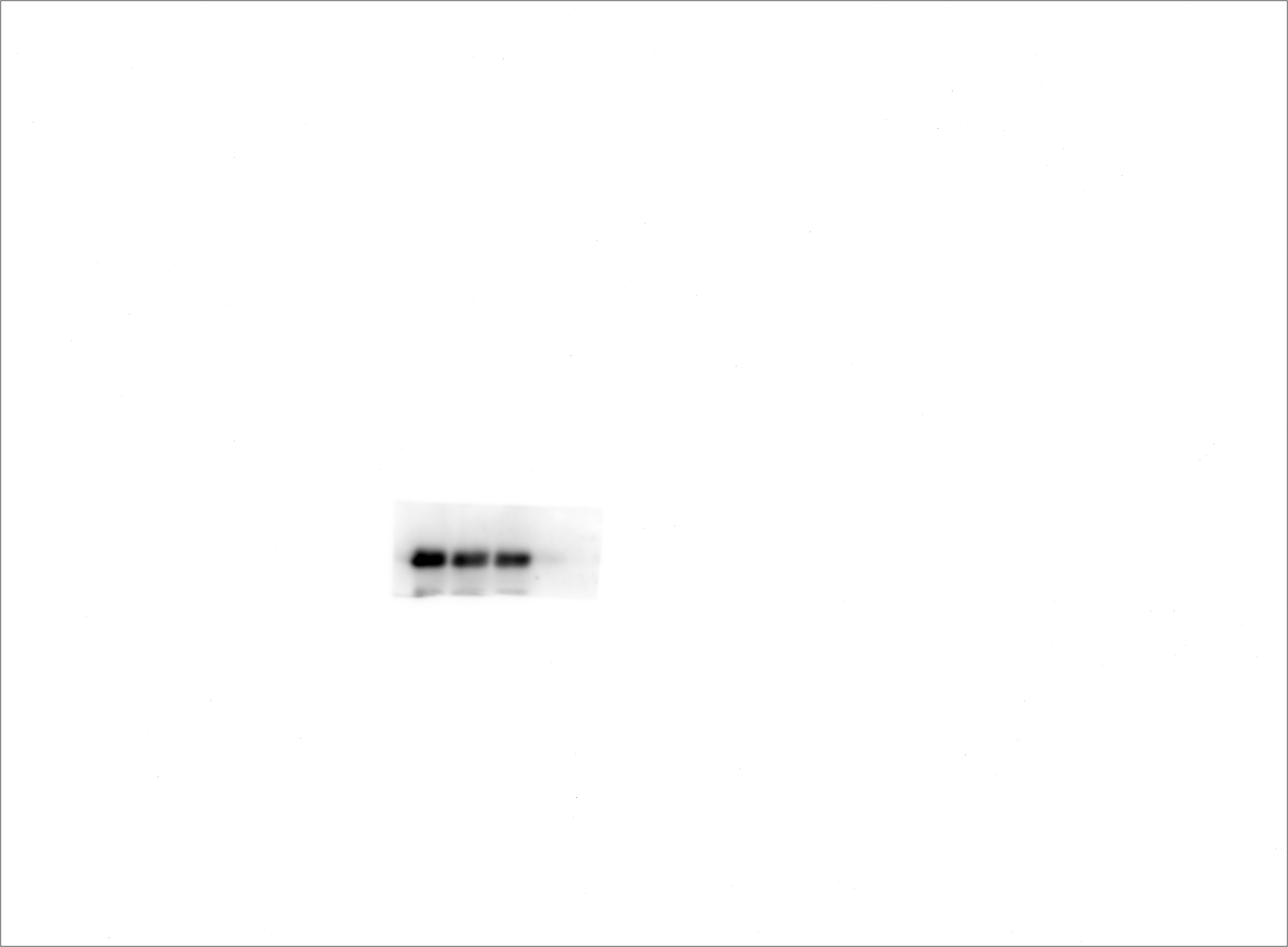

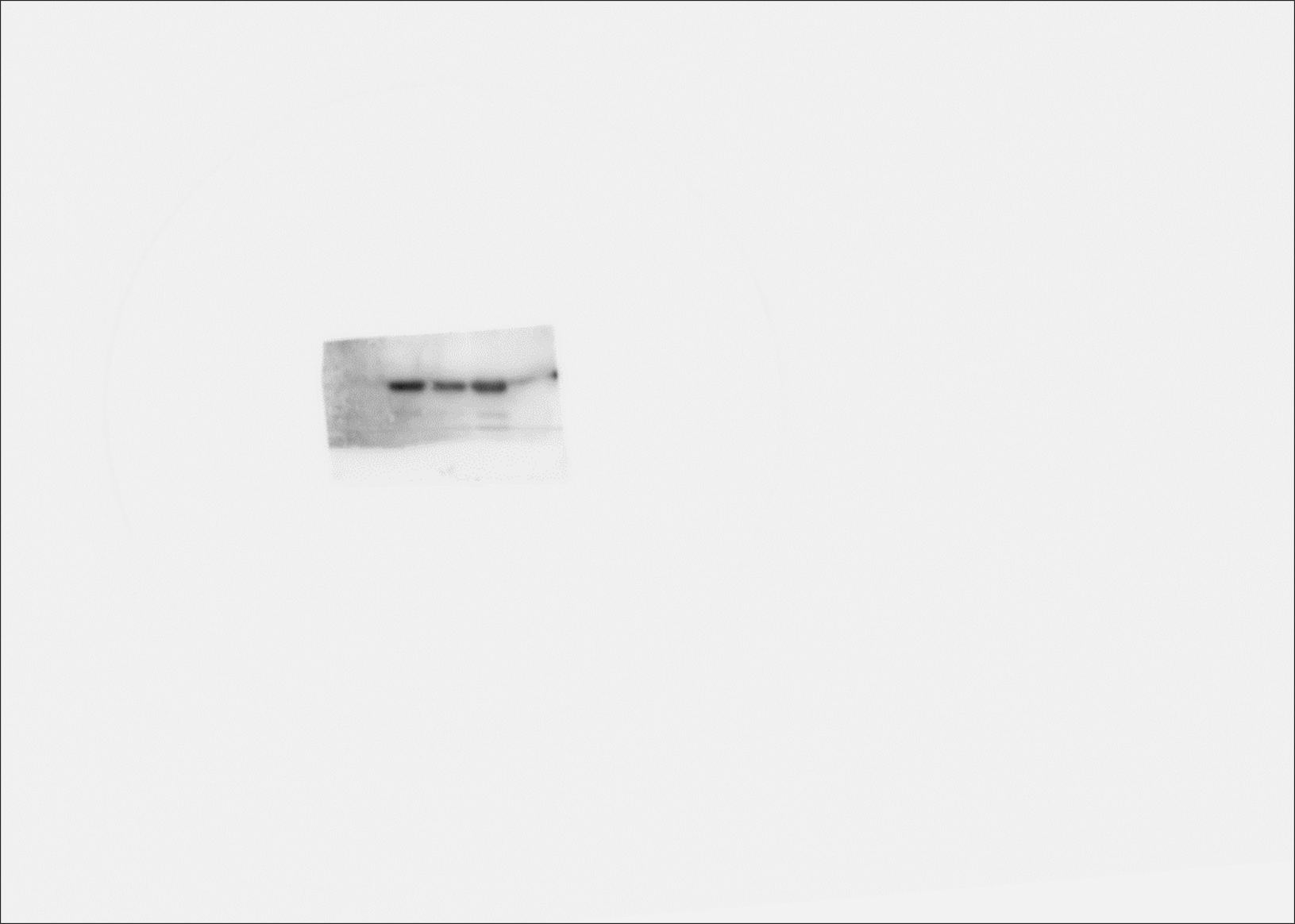


Fig 6B:


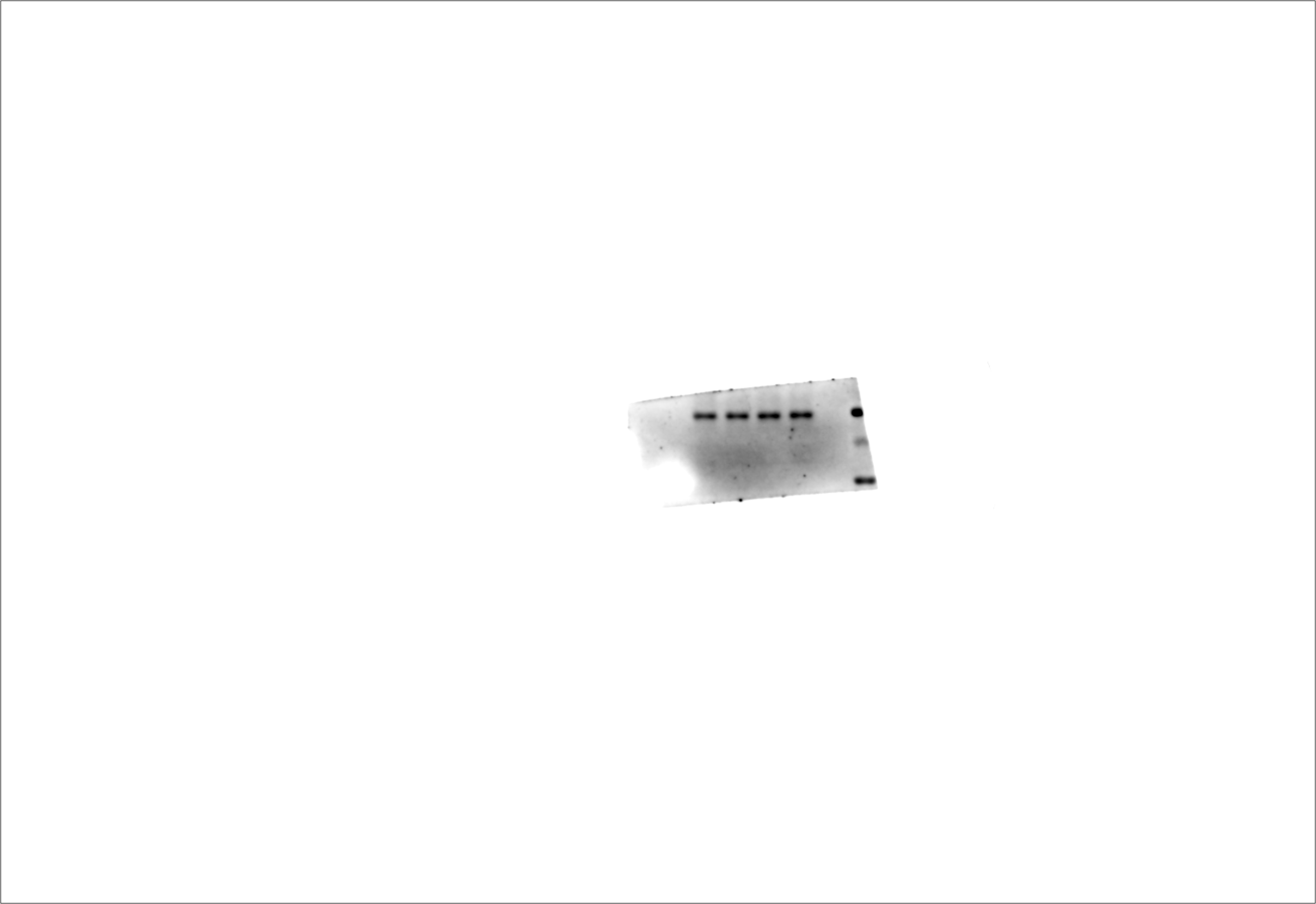

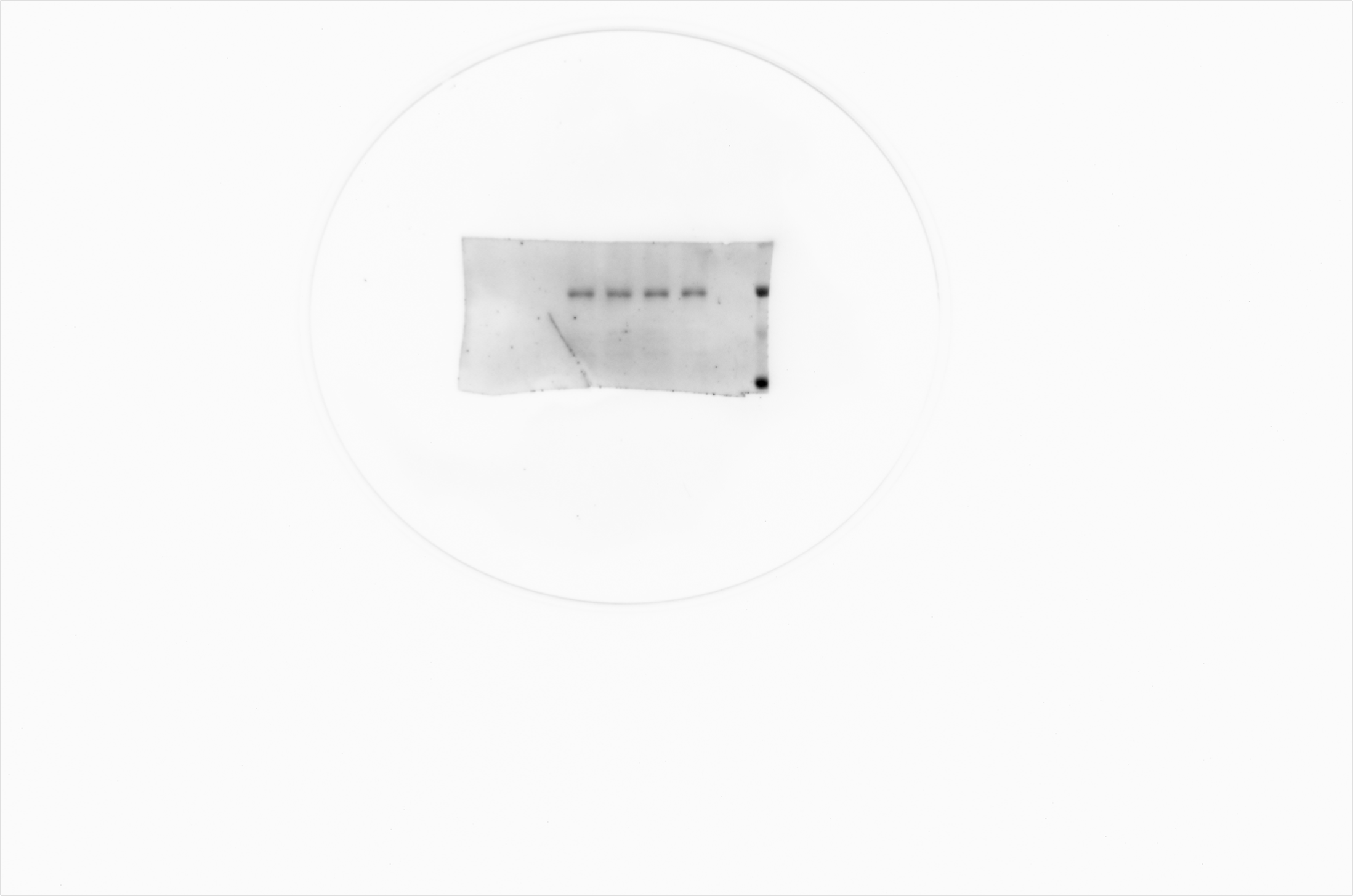

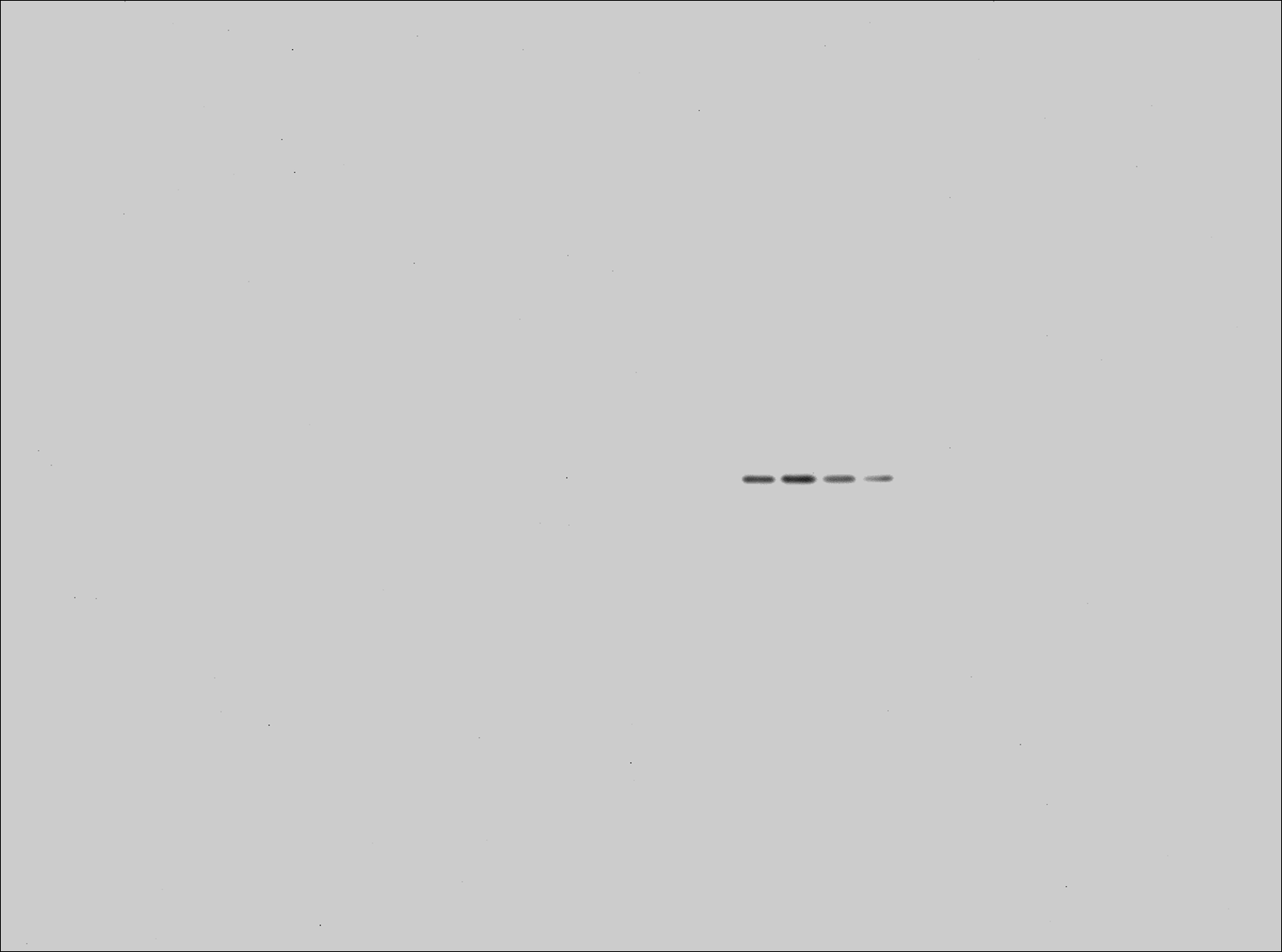

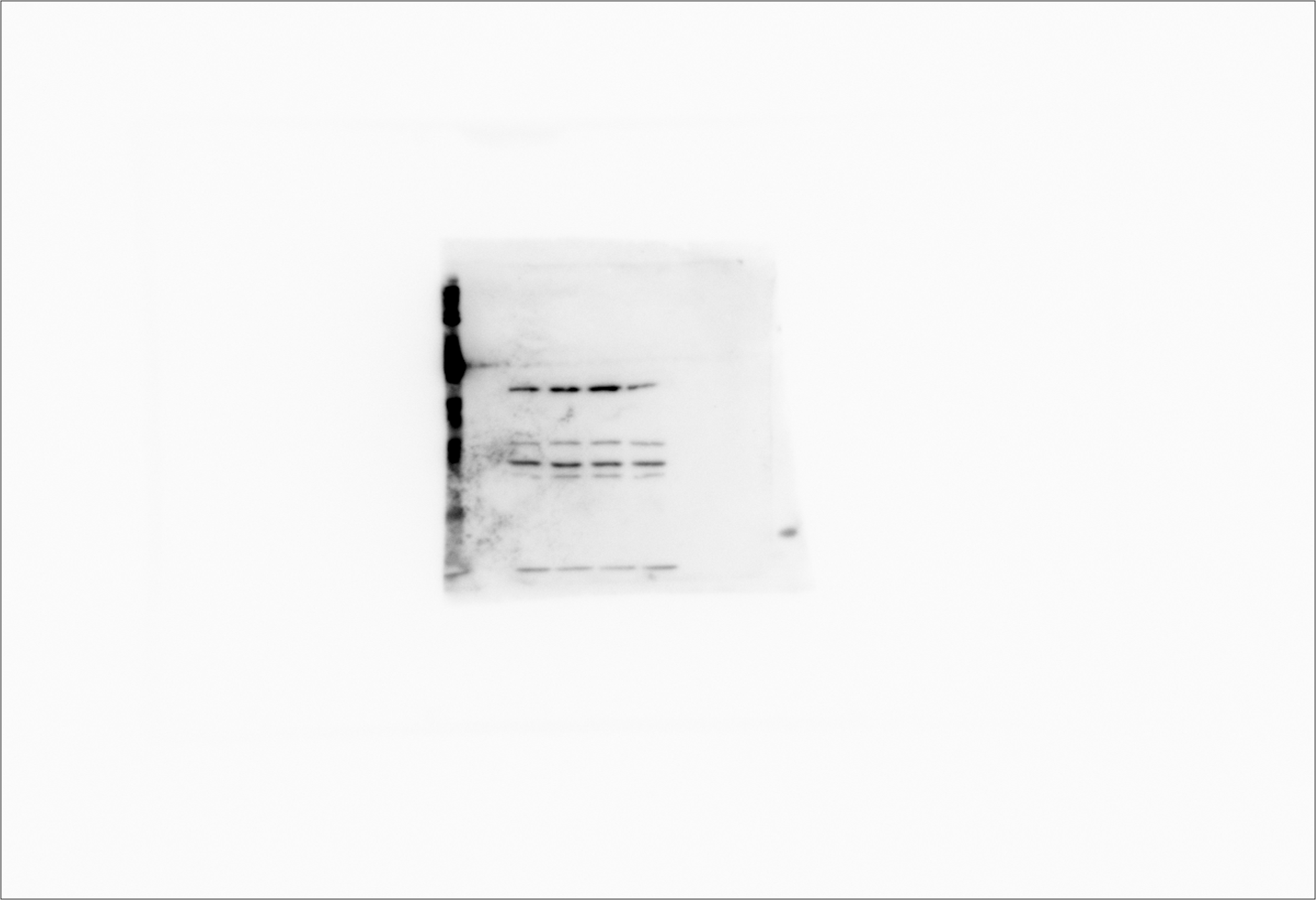

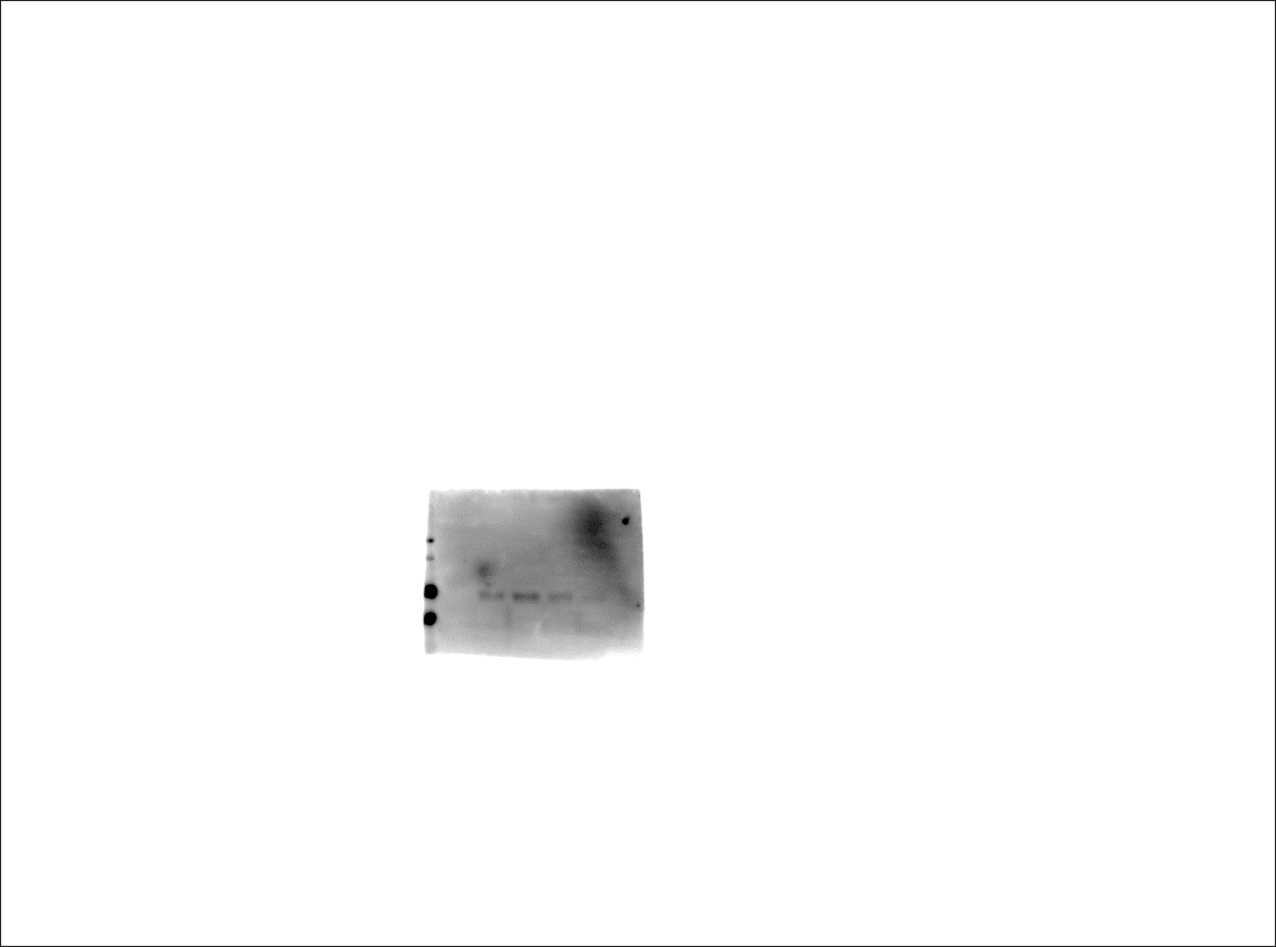

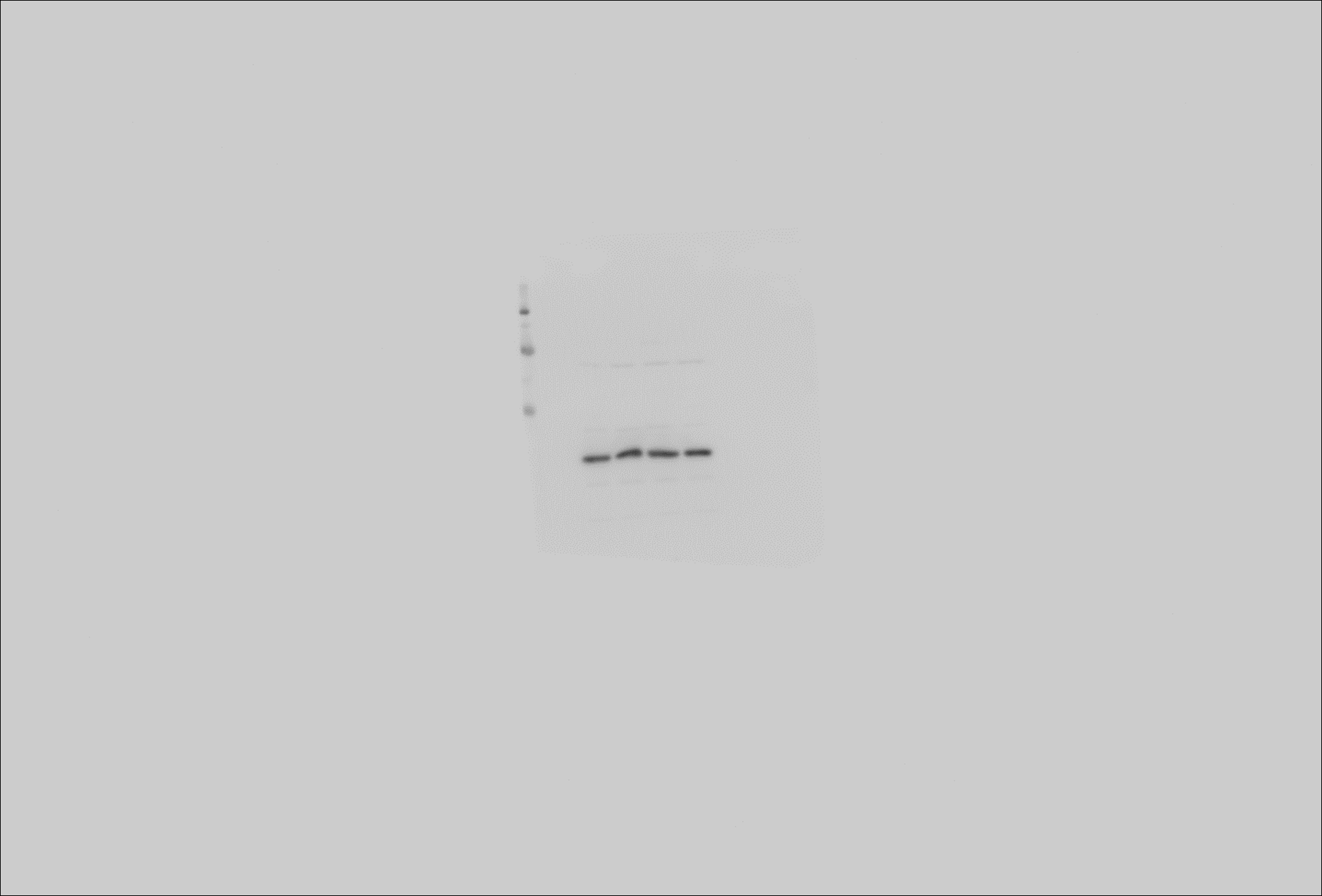

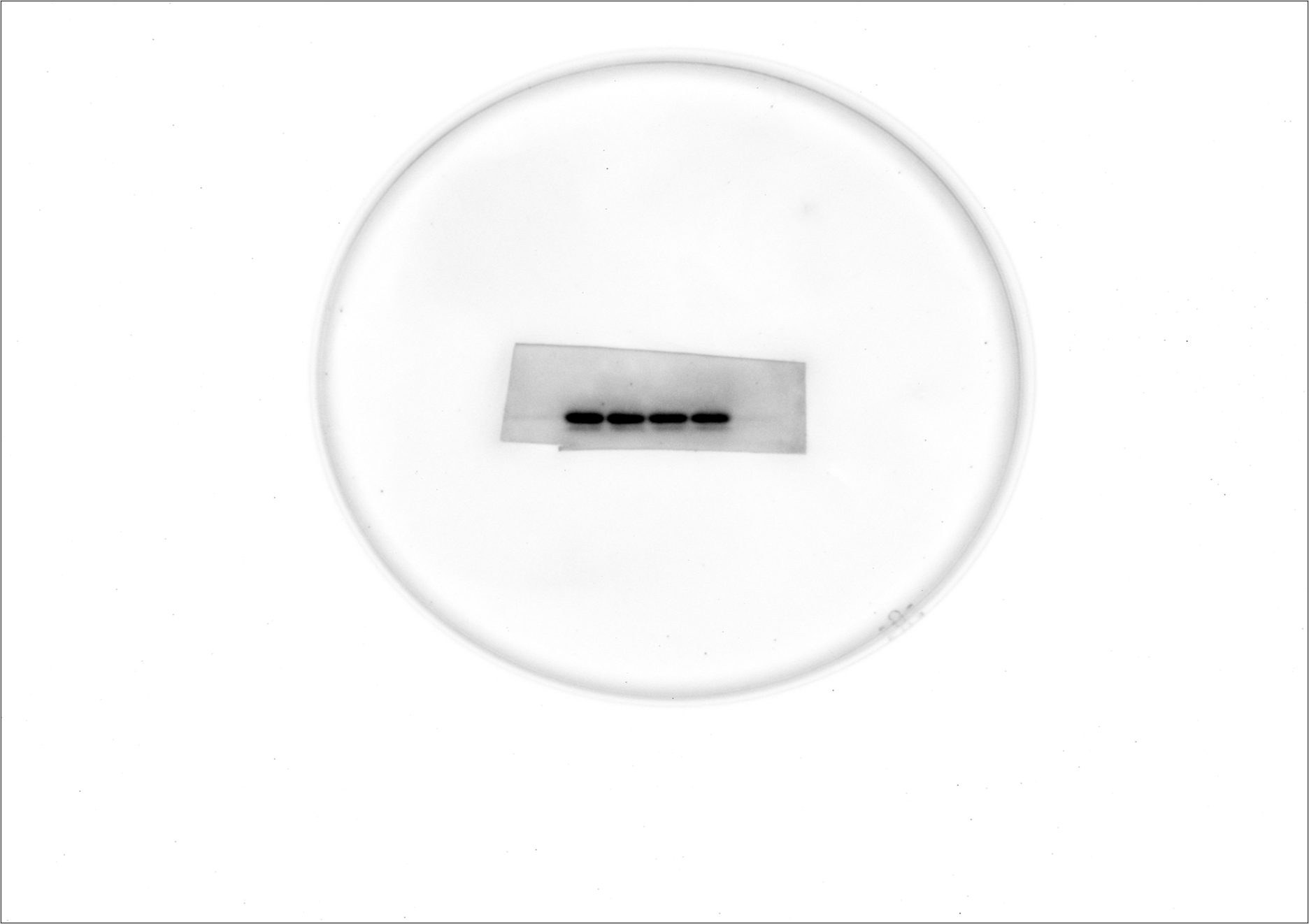

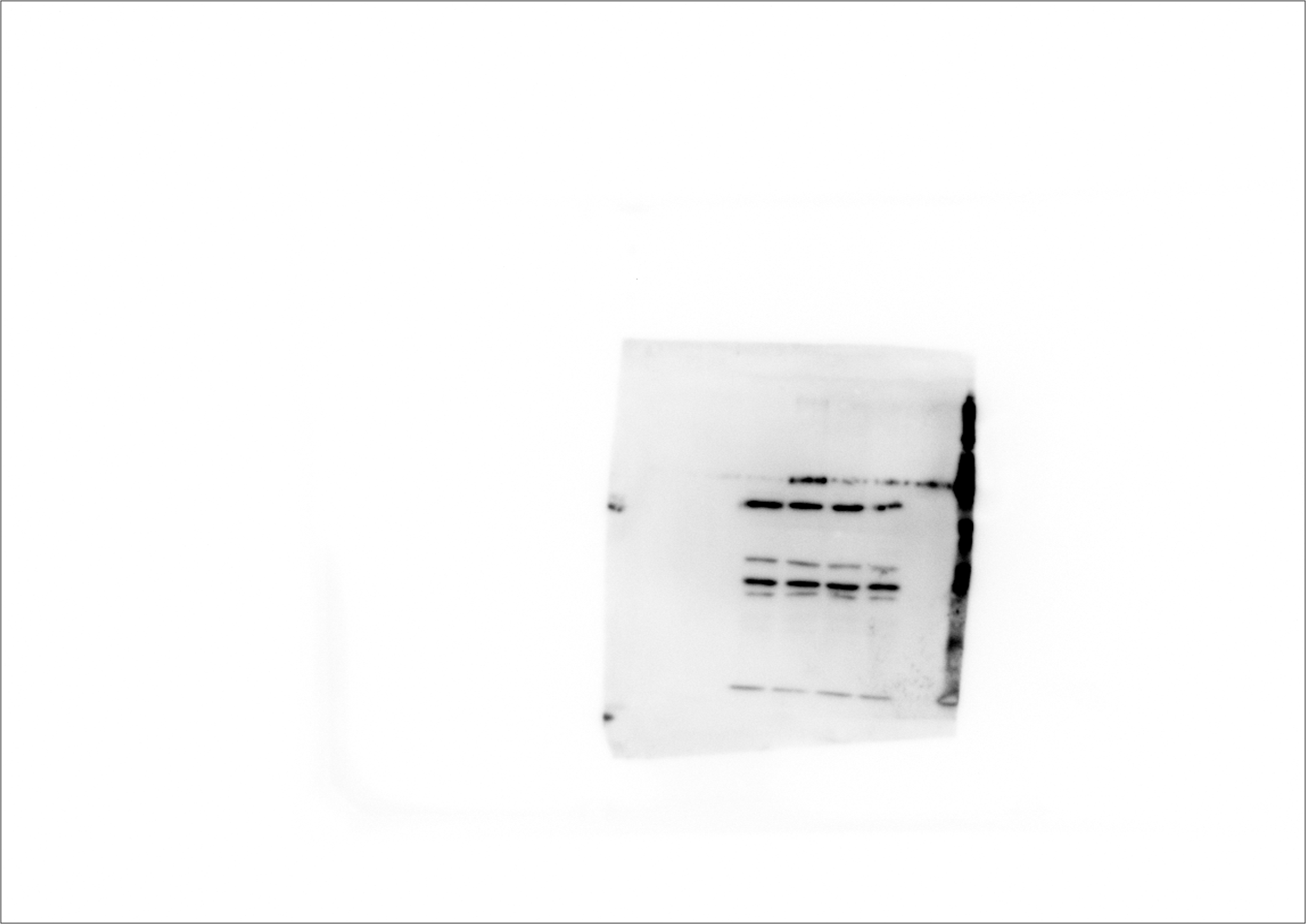

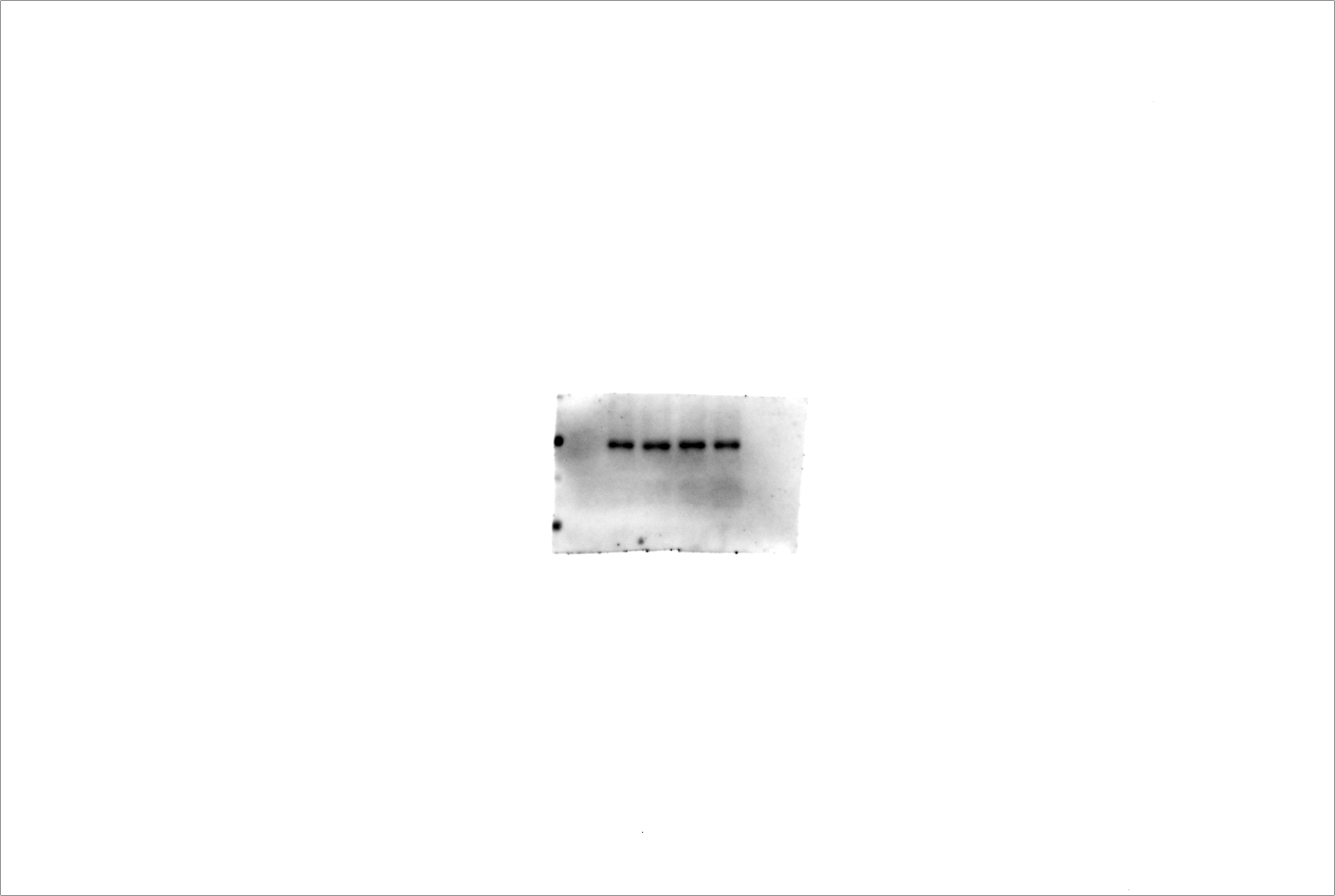


Fig 6E:


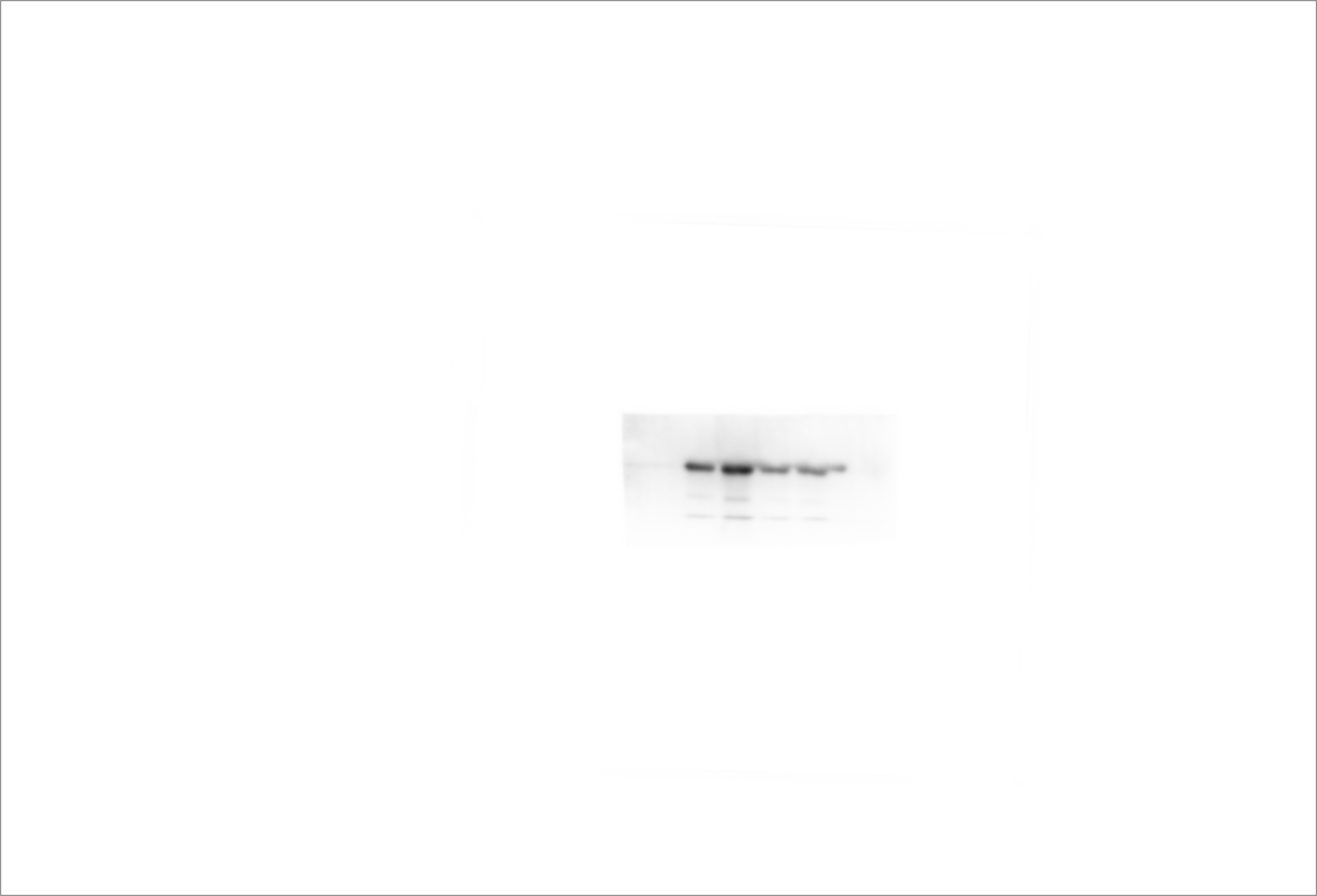

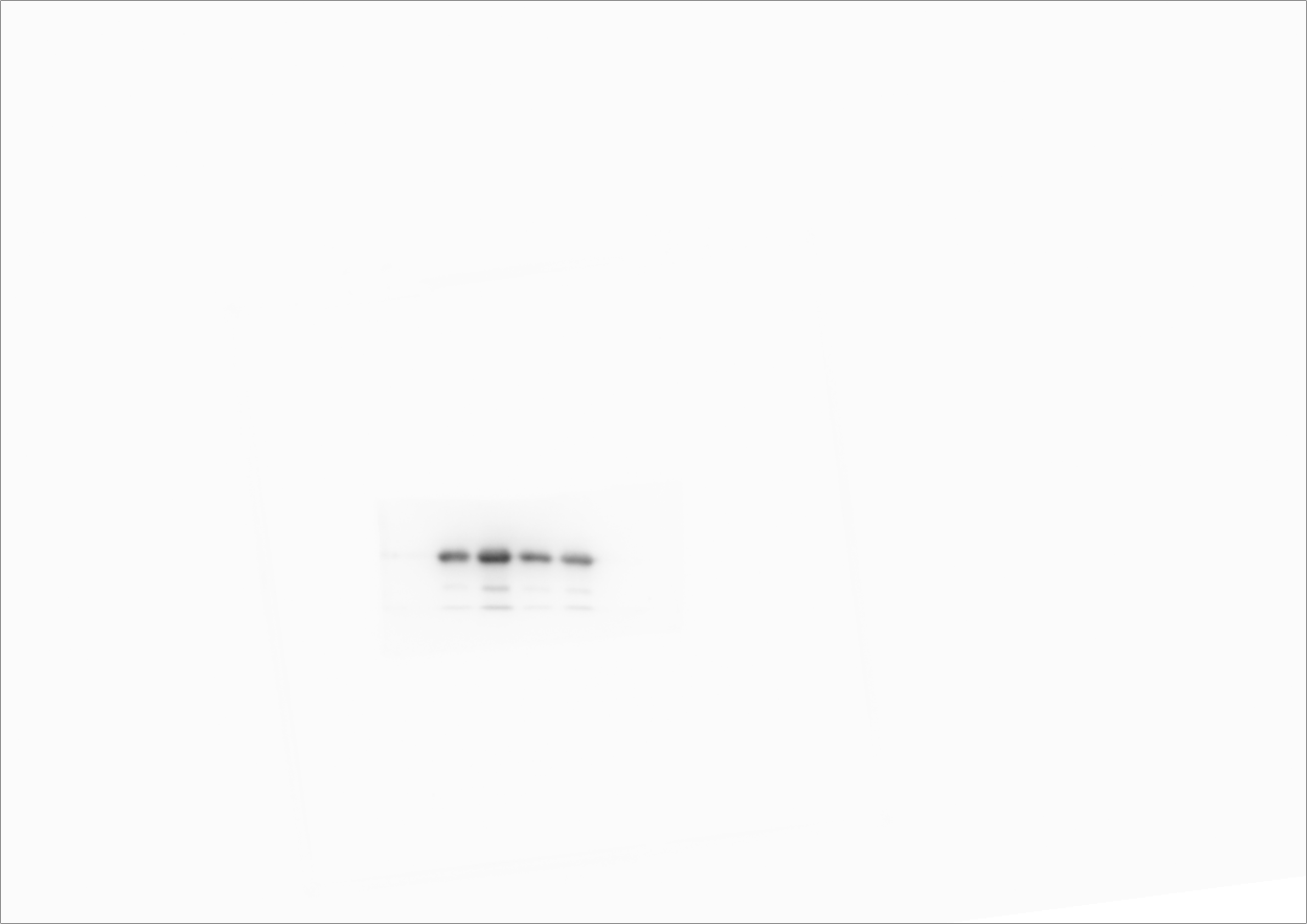

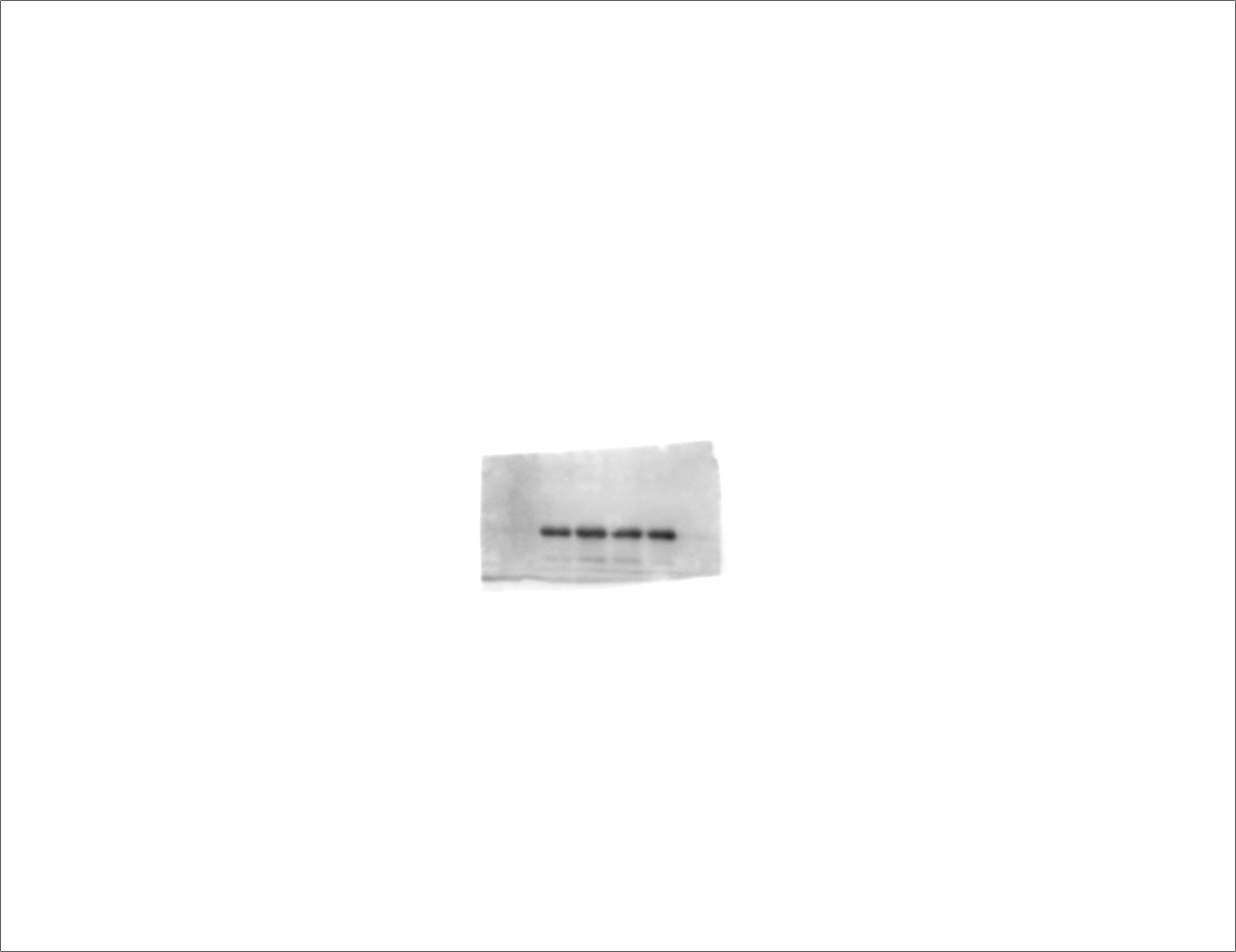

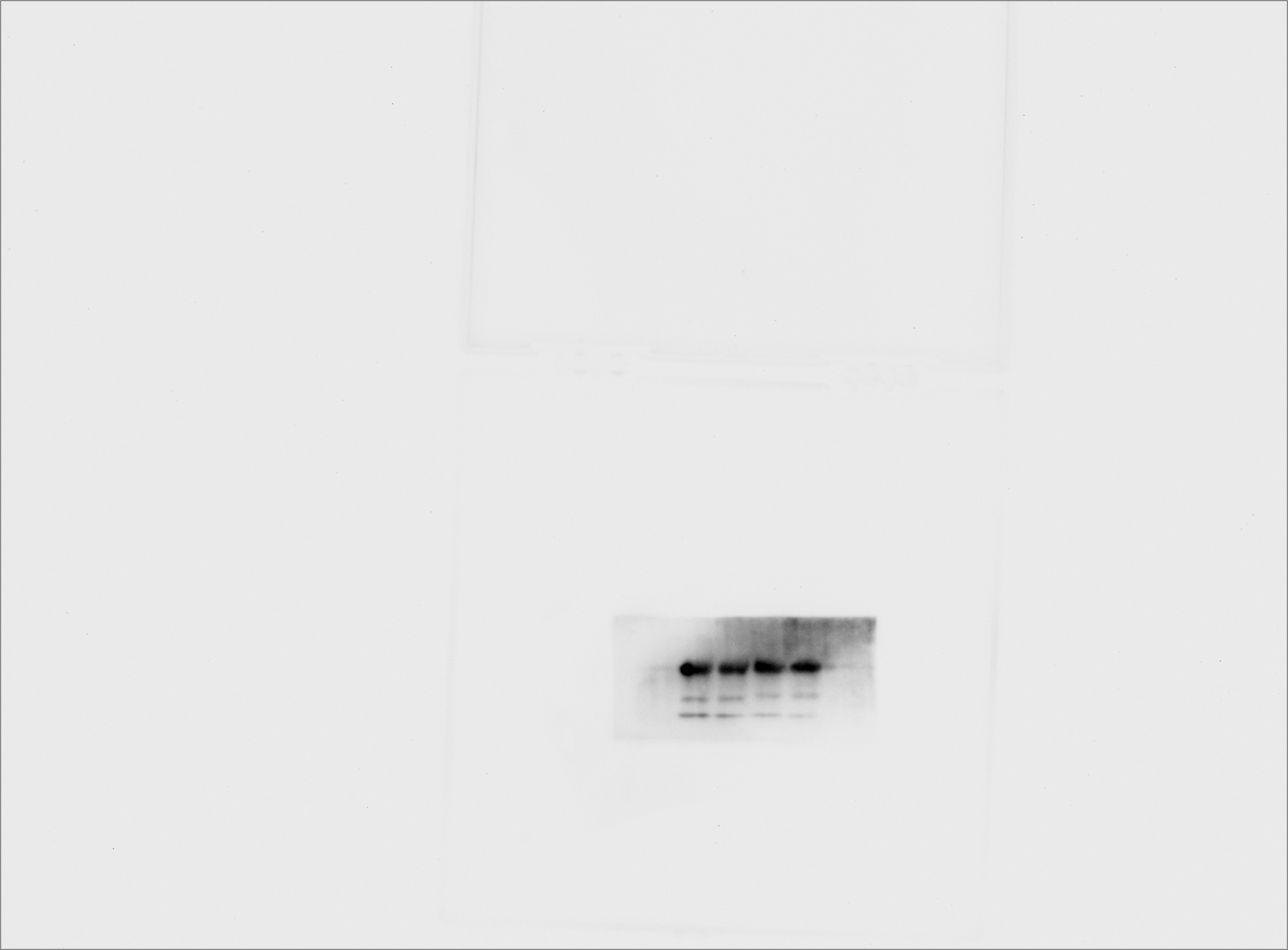

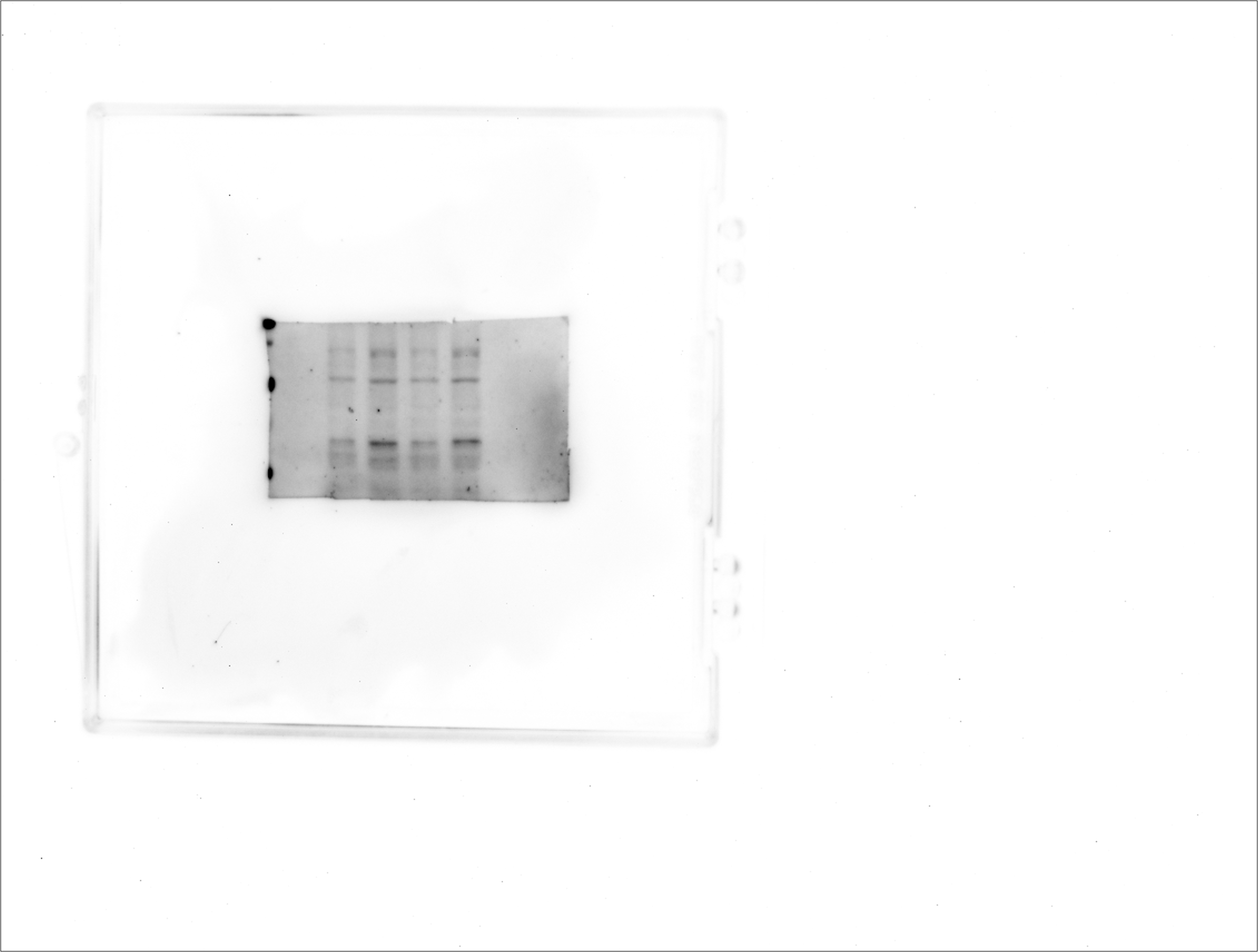

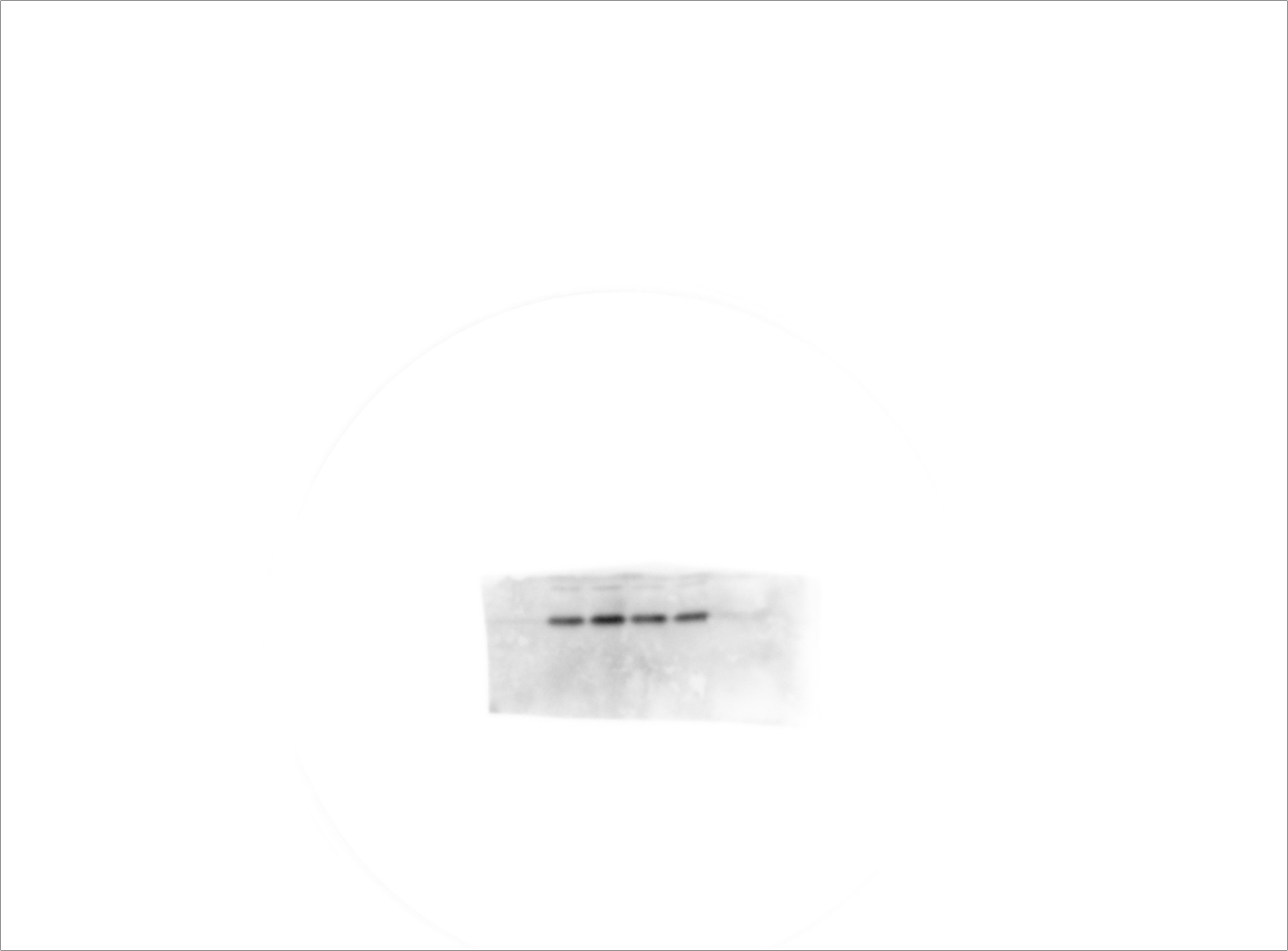

Supplement: Supplementary file 5 — Supplementary Material 5 [file 10020_2023_633_MOESM5_ESM.doc]
