# Supplementary material for: Receptor tyrosine kinase C-kit promotes a destructive phenotype of FLS in osteoarthritis via intracellular EMT signaling
Source: Mol Med. 2023 Mar 23;29:38. doi: 10.1186/s10020-023-00633-6 (PMC10037859; doi:10.1186/s10020-023-00633-6)
Supplement: Supplementary file 2 — Supplementary Material 2 [file 10020_2023_633_MOESM2_ESM.docx]

|  | gender | age | source | Public date | clinical status |
| --- | --- | --- | --- | --- | --- |
| GSM302876 | F | 77 | GSE12021 | Sep 02 2008 | osteoarthritis |
| GSM302880 | F | 71 | GSE12021 | Sep 02 2008 | osteoarthritis |
| GSM302930 | F | 76 | GSE12021 | Sep 02 2008 | osteoarthritis |
| GSM303326 | F | 61 | GSE12021 | Sep 02 2008 | osteoarthritis |
| GSM303341 | F | 75 | GSE12021 | Sep 02 2008 | osteoarthritis |
| GSM303356 | M | 78 | GSE12021 | Sep 02 2008 | osteoarthritis |
| GSM303358 | M | 64 | GSE12021 | Sep 02 2008 | osteoarthritis |
| GSM303360 | F | 71 | GSE12021 | Sep 02 2008 | osteoarthritis |
| GSM303362 | F | 80 | GSE12021 | Sep 02 2008 | osteoarthritis |
| GSM303370 | F | 66 | GSE12021 | Sep 02 2008 | osteoarthritis |
| GSM800602 | M | 61 | GSE32317 | Nov 01 2011 | osteoarthritis |
| GSM800603 | F | 78 | GSE32317 | Nov 01 2011 | osteoarthritis |
| GSM800604 | M | 71 | GSE32317 | Nov 01 2011 | osteoarthritis |
| GSM800605 | F | 66 | GSE32317 | Nov 01 2011 | osteoarthritis |
| GSM800606 | M | 67 | GSE32317 | Nov 01 2011 | osteoarthritis |
| GSM800607 | M | 79 | GSE32317 | Nov 01 2011 | osteoarthritis |
| GSM800608 | F | 72 | GSE32317 | Nov 01 2011 | osteoarthritis |
| GSM800609 | F | 61 | GSE32317 | Nov 01 2011 | osteoarthritis |
| GSM800610 | M | 67 | GSE32317 | Nov 01 2011 | osteoarthritis |
| GSM800611 | M | 77 | GSE32317 | Nov 01 2011 | osteoarthritis |
| GSM800612 | M | 70 | GSE32317 | Nov 01 2011 | osteoarthritis |
| GSM800613 | M | 56 | GSE32317 | Nov 01 2011 | osteoarthritis |
| GSM800614 | M | 71 | GSE32317 | Nov 01 2011 | osteoarthritis |
| GSM800615 | F | 55 | GSE32317 | Nov 01 2011 | osteoarthritis |
| GSM800616 | M | 75 | GSE32317 | Nov 01 2011 | osteoarthritis |
| GSM800617 | F | 49 | GSE32317 | Nov 01 2011 | osteoarthritis |
| GSM800618 | M | 68 | GSE32317 | Nov 01 2011 | osteoarthritis |
| GSM800619 | M | 55 | GSE32317 | Nov 01 2011 | osteoarthritis |
| GSM800620 | M | 47 | GSE32317 | Nov 01 2011 | osteoarthritis |
| GSM1332211 | none | none | GSE55235 | Mar 05 2014 | osteoarthritis |
| GSM1332220 | none | none | GSE55235 | Mar 05 2014 | osteoarthritis |
| GSM1332212 | none | none | GSE55235 | Mar 05 2014 | osteoarthritis |
| GSM1332213 | none | none | GSE55235 | Mar 05 2014 | osteoarthritis |
| GSM1332214 | none | none | GSE55235 | Mar 05 2014 | osteoarthritis |
| GSM1332215 | none | none | GSE55235 | Mar 05 2014 | osteoarthritis |
| GSM1332216 | none | none | GSE55235 | Mar 05 2014 | osteoarthritis |
| GSM1332217 | none | none | GSE55235 | Mar 05 2014 | osteoarthritis |
| GSM1332218 | none | none | GSE55235 | Mar 05 2014 | osteoarthritis |
| GSM1332219 | none | none | GSE55235 | Mar 05 2014 | osteoarthritis |
| GSM1337334 | F | 71 | GSE55457 | Feb 21 2014 | osteoarthritis |
| GSM1337335 | F | 80 | GSE55457 | Feb 21 2014 | osteoarthritis |
| GSM1337336 | F | 66 | GSE55457 | Feb 21 2014 | osteoarthritis |
| GSM1337327 | F | 77 | GSE55457 | Feb 21 2014 | osteoarthritis |
| GSM1337328 | F | 71 | GSE55457 | Feb 21 2014 | osteoarthritis |
| GSM1337332 | M | 78 | GSE55457 | Feb 21 2014 | osteoarthritis |
| GSM1337333 | M | 69 | GSE55457 | Feb 21 2014 | osteoarthritis |
| GSM1337331 | F | 75 | GSE55457 | Feb 21 2014 | osteoarthritis |
| GSM1337329 | F | 76 | GSE55457 | Feb 21 2014 | osteoarthritis |
| GSM1337330 | F | 61 | GSE55457 | Feb 21 2014 | osteoarthritis |
| GSM1339628 | F | 71 | GSE55584 | Mar 05 2014 | osteoarthritis |
| GSM1339629 | F | 71 | GSE55584 | Mar 05 2014 | osteoarthritis |
| GSM1339630 | F | 85 | GSE55584 | Mar 05 2014 | osteoarthritis |
| GSM1339631 | F | 77 | GSE55584 | Mar 05 2014 | osteoarthritis |
| GSM1339632 | F | 59 | GSE55584 | Mar 05 2014 | osteoarthritis |
| GSM1339633 | F | 76 | GSE55584 | Mar 05 2014 | osteoarthritis |
| GSM2183539 | none | none | GSE82107 | Jun 02 2016 | osteoarthritis |
| GSM2183548 | none | none | GSE82107 | Jun 02 2016 | osteoarthritis |
| GSM2183540 | none | none | GSE82107 | Jun 02 2016 | osteoarthritis |
| GSM2183541 | none | none | GSE82107 | Jun 02 2016 | osteoarthritis |
| GSM2183542 | none | none | GSE82107 | Jun 02 2016 | osteoarthritis |
| GSM2183543 | none | none | GSE82107 | Jun 02 2016 | osteoarthritis |
| GSM2183544 | none | none | GSE82107 | Jun 02 2016 | osteoarthritis |
| GSM2183545 | none | none | GSE82107 | Jun 02 2016 | osteoarthritis |
| GSM2183546 | none | none | GSE82107 | Jun 02 2016 | osteoarthritis |
| GSM2183547 | none | none | GSE82107 | Jun 02 2016 | osteoarthritis |
| GSM2460733 | F | 71 | GSE93698 | Jun 30 2017 | osteoarthritis |
| GSM2460734 | F | 73 | GSE93698 | Jun 30 2017 | osteoarthritis |
| GSM2460735 | F | 65 | GSE93698 | Jun 30 2017 | osteoarthritis |
| GSM2460736 | F | 51 | GSE93698 | Jun 30 2017 | osteoarthritis |
| GSM2460737 | M | 56 | GSE93698 | Jun 30 2017 | osteoarthritis |
