# Supplementary material for: Receptor tyrosine kinase C-kit promotes a destructive phenotype of FLS in osteoarthritis via intracellular EMT signaling
Source: Mol Med. 2023 Mar 23;29:38. doi: 10.1186/s10020-023-00633-6 (PMC10037859; doi:10.1186/s10020-023-00633-6)
Supplement: Supplementary file 3 — Supplementary Material 3 [file 10020_2023_633_MOESM3_ESM.docx]

| **Gene Name** | **Classification of Receptors** |
| --- | --- |
| **ITGA1** | Integrin receptors |
| **ITGA10** | Integrin receptors |
| **ITGA11** | Integrin receptors |
| **ITGA2** | Integrin receptors |
| **ITGA2B** | Integrin receptors |
| **ITGA3** | Integrin receptors |
| **ITGA4** | Integrin receptors |
| **ITGA5** | Integrin receptors |
| **ITGA6** | Integrin receptors |
| **ITGA7** | Integrin receptors |
| **ITGA8** | Integrin receptors |
| **ITGA9** | Integrin receptors |
| **ITGAD** | Integrin receptors |
| **ITGAE** | Integrin receptors |
| **ITGAL** | Integrin receptors |
| **ITGAM** | Integrin receptors |
| **ITGAV** | Integrin receptors |
| **ITGAX** | Integrin receptors |
| **ITGB1** | Integrin receptors |
| **ITGB2** | Integrin receptors |
| **ITGB3** | Integrin receptors |
| **ITGB4** | Integrin receptors |
| **ITGB5** | Integrin receptors |
| **ITGB6** | Integrin receptors |
| **ITGB7** | Integrin receptors |
| **ITGB8** | Integrin receptors |
| **ACVR1** | TGFβ receptors |
| **ACVR1B** | TGFβ receptors |
| **ACVR1C** | TGFβ receptors |
| **ACVR2A** | TGFβ receptors |
| **ACVR2B** | TGFβ receptors |
| **ACVRL1** | TGFβ receptors |
| **AMHR2** | TGFβ receptors |
| **BMPR1A** | TGFβ receptors |
| **BMPR1B** | TGFβ receptors |
| **BMPR2** | TGFβ receptors |
| **TGFBR1** | TGFβ receptors |
| **TGFBR2** | TGFβ receptors |
| **WNT2B** | WNT receptors |
| **WNT5B** | WNT receptors |
| **DLK1** | Delta-like receptors |
| **DLK2** | Delta-like receptors |
| **DLL1** | Delta-like receptors |
| **DLL3** | Delta-like receptors |
| **DLL4** | Delta-like receptors |
| **IL6R** | IL-6 receptors |
| **IL6ST** | IL-7 receptors |
| **SMO** | SMO receptors |
| **JAG1** | JAG1 receptors |
| **ALK** | RTKs |
| **LTK** | RTKs |
| **AXL** | RTKs |
| **MER** | RTKs |
| **TYRO3** | RTKs |
| **DDR1** | RTKs |
| **DDR2** | RTKs |
| **EGFR** | RTKs |
| **ERBB2** | RTKs |
| **ERBB3** | RTKs |
| **ERBB4** | RTKs |
| **EPHA1** | RTKs |
| **EPHA2** | RTKs |
| **EPHA3** | RTKs |
| **EPHA4** | RTKs |
| **EPHA5** | RTKs |
| **EPHA6** | RTKs |
| **EPHA7** | RTKs |
| **EPHA8** | RTKs |
| **EPHB1** | RTKs |
| **EPHB2** | RTKs |
| **EPHB3** | RTKs |
| **EPHB4** | RTKs |
| **EPHB6** | RTKs |
| **EPHX** | RTKs |
| **FGFR1** | RTKs |
| **FGFR2** | RTKs |
| **FGFR3** | RTKs |
| **FGFR4** | RTKs |
| **IGF1R** | RTKs |
| **INSR** | RTKs |
| **INSRR** | RTKs |
| **MET** | RTKs |
| **RON** | RTKs |
| **MUSK** | RTKs |
| **CSF1R** | RTKs |
| **FLT3** | RTKs |
| **KIT** | RTKs |
| **PDGFRA** | RTKs |
| **PDGFRB** | RTKs |
| **PTK7** | RTKs |
| **RET** | RTKs |
| **ROR1** | RTKs |
| **ROR2** | RTKs |
| **ROS1** | RTKs |
| **RYK** | RTKs |
| **TEK** | RTKs |
| **TIE** | RTKs |
| **NTRK1** | RTKs |
| **NTRK2** | RTKs |
| **NTRK3** | RTKs |
| **VEGFR1** | RTKs |
| **VEGFR2** | RTKs |
| **VEGFR3** | RTKs |
| **AATYK** | RTKs |
| **AATYK2** | RTKs |
| **AATYK3** | RTKs |
| **DKFZp761P1010** | RTKs |
