# Supplementary material for: Receptor tyrosine kinase C-kit promotes a destructive phenotype of FLS in osteoarthritis via intracellular EMT signaling
Source: Mol Med. 2023 Mar 23;29:38. doi: 10.1186/s10020-023-00633-6 (PMC10037859; doi:10.1186/s10020-023-00633-6)
Supplement: Supplementary file 4 — Visualization for supplementary table 3?K/L grade: Kellgren & Lawrence grade (0-4 grade). [file 10020_2023_633_MOESM4_ESM.docx]

| Non-OA | | | |  |  |
| --- | --- | --- | --- | --- | --- |
| ID | Gender | Age | K/L grade |  |  |
| 1 | Female | 37 | 0 |  |  |
| 2 | Female | 38 | 0 |  |  |
| 3 | Female | 48 | 0 |  |  |
| 4 | Female | 53 | 0 |  |  |
| 5 | Male | 57 | 0 |  |  |
| 6 | Male | 41 | 0 |  |  |
| 7 | Male | 38 | 0 |  |  |
| 8 | Male | 49 | 0 |  |  |
| 9 | Male | 36 | 0 |  |  |
| 10 | Male | 44 | 0 |  |  |
| OA | | | |  |  |
| ID | Gender | Age | K/L grade |  |  |
| 11 | Female | 62 | 4 |  |  |
| 12 | Female | 56 | 4 |  |  |
| 13 | Female | 61 | 4 |  |  |
| 14 | Female | 58 | 4 |  |  |
| 15 | Female | 66 | 4 |  |  |
| 16 | Female | 67 | 4 |  |  |
| 17 | Female | 55 | 4 |  |  |
| 18 | Female | 64 | 4 |  |  |
| 19 | Male | 59 | 4 |  |  |
| 20 | Male | 66 | 4 |  |  |
|  |  |  |  |  |  |
| Visualization for supplementary table 3：K/L grade: Kellgren & Lawrence grade (0-4 grade). | | | | |  |
|  |  |  |  |  |  |
