# Supplementary material for: Receptor tyrosine kinase C-kit promotes a destructive phenotype of FLS in osteoarthritis via intracellular EMT signaling
Source: Mol Med. 2023 Mar 23;29:38. doi: 10.1186/s10020-023-00633-6 (PMC10037859; doi:10.1186/s10020-023-00633-6)
Supplement: Supplementary file 6 — Supplementary Figure legendsSupplementary Figure S1. (A) Safranin O/fast green staining of cartilage (up), MRI images (bottom) and OARSI semi-quantitative scores of 3 weeks after operation (right). Supplementary Table legendsSupplementary Table S1. Basic information (gender, age, source, public date and clinical status) and data sources of 70 OA patients Supplementary Table S2. 107 EMT-related receptors. Supplementary Table S3. Basic information (gender, age, K/L grade) of 10 non-OA and 10 OA patient. K/L grade: Kellgren & Lawrence grade. Supplementary Table S4. The human and rat primer sequence for qPCR and knocking down. [file 10020_2023_633_MOESM6_ESM.docx]

**Supplementary Figure legends**

**Supplementary Figure S1.**

1. Safranin O/fast green staining of cartilage (up), MRI images (bottom) and OARSI semi-quantitative scores of 3 weeks after operation (right).

**Supplementary Table legends**

**Supplementary Table S1.** Basic information (gender, age, source, public date and clinical status) and data sources of 70 OA patients

**Supplementary Table S2.** 107 EMT-related receptors.

**Supplementary Table S3.** Basic information (gender, age, K/L grade) of 10 non-OA and 10 OA patient. K/L grade: Kellgren & Lawrence grade.

**Supplementary Table S4.** The human and rat primer sequence for qPCR and knocking down.
