# Supplementary material for: Receptor tyrosine kinase C-kit promotes a destructive phenotype of FLS in osteoarthritis via intracellular EMT signaling
Source: Mol Med. 2023 Mar 23;29:38. doi: 10.1186/s10020-023-00633-6 (PMC10037859; doi:10.1186/s10020-023-00633-6)
Supplement: Supplementary file 7 — Supplementary Table S4. The human and rat primer sequence. [file 10020_2023_633_MOESM7_ESM.docx]

| **Human Primers** | | |
| --- | --- | --- |
| ***GAPDH*** | Forward | GGAGCGAGATCCCTCCAAAAT |
|  | Reverse | GGCTGTTGTCATACTTCTCATGG |
| ***CDH2 (N-cadherin)*** | Forward | CAGTATCCGGTCCGATCTGC |
|  | Reverse | GAGCTGTGGGGTCATTGTCA |
| ***MMP1*** | Forward | TGTGGTGTCTCACAGCTTCC |
|  | Reverse | CGCTTTTCAACTTGCCTCCC |
| ***MMP3*** | Forward | CAGCCAACTGTGATCCTGCT |
|  | Reverse | GATTTGCGCCAAAAGTGCCT |
| ***MMP13*** | Forward | GCACTTCCCACAGTGCCTAT |
|  | Reverse | AGTTCTTCCCTTGATGGCCG |
| ***IL-1β*** | Forward | TCGCCAGTGAAATGATGGCT |
|  | Reverse | GGTCGGAGATTCGTAGCTGG |
| ***IL-6*** | Forward | CCACCGGGAACGAAAGAGAA |
|  | Reverse | GAGAAGGCAACTGGACCGAA |
| ***IL-8*** | Forward | CACTGCGCCAACACAGAAAT |
|  | Reverse | TTCTCAGCCCTCTTCAAAAACTTC |
| ***IL-32*** | Forward | CTCTCTCGGCTGAGTATTTGTG |
|  | Reverse | ACATGGCGGCCAAAAGTTC |
| ***CDK1*** | Forward | CTTGGCTTCAAAGCTGGCTC |
|  | Reverse | GGGTATGGTAGATCCCGGCT |
| ***TIMP1*** | Forward | GCGGATACTTCCACAGGTCC |
|  | Reverse | GCTAAGCTCAGGCTGTTCCA |
| ***TNFa*** | Forward | GGAGAAGGGTGACCGACTCA |
|  | Reverse | CCTCACAGGGCAATGATCCC |
| ***SAA1*** | Forward | AGATCAGGTGAGGAGCACAC |
|  | Reverse | GCTGTATGCCCCAGGATAACT |
| ***S100A8*** | Forward | TGGCCAAGCCTAACCGCTAT |
|  | Reverse | TTTCTCCAGCTCGGTCAACAT |
| ***S100A9*** | Forward | TCGGCTTTGACAGAGTGCAA |
|  | Reverse | GCCCCAGCTTCACAGAGTAT |
| **Rat Primers** | | |
| Gene Name | Sequence | |
| ***β-actin*** | Forward | CCCGCGAGTACAACCTTCTT |
|  | Reverse | CGCAGCGATATCGTCATCCA |
| ***Cdh2 (N-cadherin)*** | Forward | CTGGGAACAGGGAAAGGGAC |
|  | Reverse | CAAAGCTTCCGGGGGTAGAG |
| ***Cdh1 (E-cadherin)*** | Forward | ATCCTGGCCCTCCTGATTCT |
|  | Reverse | CGGGTATCGTCATCTGGTGG |
| ***Vimentin*** | Forward | TCCTTCGAAGCCATGTCCAC |
|  | Reverse | GTGGTCACATAGCTCCGGTT |
| **sh C-kit Primers** | | |
| ***sh C-kit #1*** | | 5’-CCGGCCCTGGTCATTACAGAATATTCTCGAGAATATTCTGTAATGACCAGGGTTTTTG-3’ |
| ***sh C-kit #2*** | | 5’-CCGGCCTTAATGATGGGAGATATATCTCGAGATATATCTCCCATCATTAAGGTTTTTG-3’ |
| ***sh C-kit #3*** | | 5’-CCGGCGGATCACAAAGATTTGCGATCTCGAGATCGCAAATCTTTGTGATCCGTTTTTG-3’ |
| Supplementary Table S4. The human and rat primer sequence. | | |
